# CDK4/6 inhibitors combined with fulvestrant for the treatment of HR+/HER2–advanced or metastatic breast cancer: a Bayesian network meta-analysis

**DOI:** 10.1186/s12885-026-15713-z

**Published:** 2026-02-17

**Authors:** Yanmin Deng, Wenrui Huang, Tao Zeng, Yuan Gao

**Affiliations:** 1 School of Pharmaceutical Sciences, Fudan University, Shanghai, 201206 China; 2 Obstetrics & Gynecology Hospital of Fudan University,Shanghai Key Lab of Reproduction and Development, Shanghai Key Lab of Female Reproductive Endocrine Related Diseases, Shanghai, 200433 China; 3 Shenzhen Traditional Chinese Medicine Hospital, Shenzhen, Guangdong China; 4 State Key Laboratory of Advanced Drug Formulations for Overcoming Delivery Barriers,Fudan University,Shanghai 201206,China, shanghai, China

**Keywords:** CDK4/6 inhibitors, Fulvestrant, HR+/HER2–advanced or metastatic breast cancer, Network meta-analysis

## Abstract

**Background:**

This study aimed to assess the efficacy and safety of different CDK4/6 inhibitors combined with fulvestrant in treating HR+/HER2– advanced or metastatic breast cancer, using a Bayesian network meta-analysis.

**Methods:**

A comprehensive search was conducted across major medical databases, including PubMed, Web of Science, Cochrane Library, and Embase, to identify randomized controlled trials (RCTs) investigating the combination of CDK4/6 inhibitors and fulvestrant for HR+/HER2– advanced or metastatic breast cancer. The search encompassed studies published from database inception to Sep 10, 2025. Relevant studies were screened, data extracted, and risk of bias evaluated. A Bayesian network meta-analysis was performed using R software (version 4.5.1).

**Results:**

Ten randomized controlled trials involving seven CDK4/6 inhibitors combined with fulvestrant were included. All regimens significantly improved progression-free survival (PFS) versus placebo + fulvestrant, and tibremciclib + fulvestrant ranked first among all regimens (HR = 0.37, 95% CrI 0.27–0.52; SUCRA = 89.26%), followed by dalpiciclib (HR = 0.42) and lerociclib (HR = 0.45). Abemaciclib + fulvestrant and palbociclib + fulvestrant demonstrated favorable overall survival (OS) improvements (HR = 0.76 and 0.79, respectively), consistent with pivotal trial evidence. For objective response rate (ORR), tibremciclib + fulvestrant ranked highest (RR = 4.0, 95% CrI 1.2–15.0), while clinical benefit rate (CBR) and quality of life (QoL) showed no statistically significant improvement compared with placebo. In safety analyses, abemaciclib + fulvestrant was associated with higher overall adverse events (RR = 1.16, 95% CrI 1.02–1.34), and bireociclib, dalpiciclib, and lerociclib showed increased risks of grade 3–4 events. Bireociclib and abemaciclib regimens also had higher rates of serious adverse events and treatment discontinuation.

**Conclusions:**

All CDK4/6 inhibitors combined with fulvestrant significantly improve progression-free survival in HR+/HER2 − advanced breast cancer, with abemaciclib and palbociclib also showing overall survival benefits. Tibremciclib plus fulvestrant showed relatively higher efficacy, demonstrating moderately superior performance to other regimens, while abemaciclib plus fulvestrant was associated with a higher incidence of treatment-related adverse events. These findings confirm the class effect of CDK4/6 inhibition and highlight the need to balance efficacy with tolerability. Future biomarker-informed and real-world studies are warranted to optimize treatment sequencing and support personalized therapeutic decisions.

**Supplementary Information:**

The online version contains supplementary material available at 10.1186/s12885-026-15713-z.

## Background

Breast cancer is the second most common cancer worldwide, with incidence and mortality rates continuing to rise according to the 2022 Global Cancer Statistics [1]. Beyond its physical burden, many patients experience emotional and social challenges that diminish quality of life [2]. Despite advances in screening and treatment, 30–40% of early-stage cases eventually progress to advanced disease, and hormone receptor-positive, human epidermal growth factor receptor 2-negative (HR+/HER2−) tumors represent about 60–65% of these cases [3, 4].

For patients with HR+/HER2 − breast cancer, endocrine therapy (ET) remains the foundation of systemic treatment. However, resistance inevitably develops through genetic and molecular alterations, limiting long-term efficacy [5]. Both intrinsic and acquired resistance mechanisms—such as loss of pRb function, TP53 mutations, and activation of alternative signaling pathways like PI3K/AKT/mTOR—play critical roles in therapy failure. Aromatase inhibitor (AI) plus CDK4/6 inhibitor is the first-line standard for advanced disease, delaying tumor progression via estrogen synthesis inhibition and cell cycle regulation, indicated for postmenopausal patients without AI contraindications. CDK4/6 inhibitor + fulvestrant targets AI-contraindicated patients (e.g., poor AI tolerability, pre-/peri-menopause, severe osteoporosis) or those with progression after AI-based endocrine therapy. It overcomes AI resistance by downregulating estrogen receptors and blocking signaling pathways, exerting synergistic anti-tumor effects. Liquid biopsy offers a promising approach to monitor these resistance biomarkers dynamically, providing insights into tumor evolution and supporting treatment adaptation. Understanding these processes is essential for optimizing subsequent therapeutic strategies [6]. Clinical studies and RCTs confirm superior response rates, progression-free survival (PFS), and clinical outcomes compared with fulvestrant monotherapy [7–9].

Beyond controlled trial settings, emerging translational and real-world studies have provided new insights into treatment heterogeneity and resistance. Real-world analyses, such as the GIM14/BIOMETA study, indicate that endocrine-resistant tumors show poorer outcomes with CDK4/6 inhibitor–based therapy, regardless of HER2-low status [10]. Molecular profiling and circulating tumor DNA (ctDNA) studies reveal heterogeneous resistance mechanisms—such as ESR1, TP53, and RB1 alterations—that shape disease progression and therapeutic response [6, 11].

Despite these advances, the comparative efficacy and safety among different CDK4/6 inhibitors remain uncertain. Existing meta-analyses provide inconsistent results and often lack comprehensive head-to-head comparisons [12]. To address these limitations, we conducted a network meta-analysis to evaluate the relative efficacy and safety of CDK4/6 inhibitors combined with fulvestrant in HR+/HER2 − advanced or metastatic breast cancer, aiming to generate evidence to inform clinical decision-making.

## Methods

This systematic review is registered in the PROSPERO International Prospective Register of Systematic Reviews (Registration Number: CRD42024626397). The study follows the guidelines outlined in the Preferred Reporting Items for Systematic Reviews and Meta-Analyses for Network Meta-Analyses (PRISMA-NMA) [13, 14] to ensure methodological rigor and transparency, as shown in Appendix 1 in Supplementary Material.

### Search strategy

A comprehensive search was conducted across major medical databases, including PubMed, Web of Science, Cochrane Library, and Embase, covering literature from inception to September 10, 2025. The search strategy combined controlled vocabulary and free-text keywords. Key terms included “Breast Neoplasms,” “CDK4/6 Inhibitors,” “Abemaciclib,” “Ribociclib,” “Palbociclib,” “Dalpiciclib,” “Lerociclib,” “Bireociclib,” “Tibremciclib,” “Fulvestrant,” and “Randomized Controlled Trial,” with Boolean operators applied to refine and structure the queries. The detailed search strategy is outlined in Appendix 2 in Supplementary Material.

### Inclusion criteria

This network meta-analysis included randomized controlled trials (RCTs) evaluating the efficacy and safety of CDK4/6 inhibitors combined with fulvestrant for treating HR+/HER2– advanced breast cancer, focusing on Phase II and III clinical trials. Eligible participants were patients with histologically or cytologically confirmed HR+/HER2– advanced breast cancer. The intervention group received one of the approved CDK4/6 inhibitors—palbociclib, ribociclib, abemaciclib, dalpiciclib, lerociclib, bireociclib or tibremciclib—in combination with fulvestrant. Control groups were categorized as fulvestrant plus placebo. Studies were required to report at least one outcome, including efficacy measures such as PFS, Overall Survival (OS), objective response rate (ORR), clinical benefit rate (CBR), or health-related quality of life (QoL), and safety measures such as the incidence of all-grade adverse events (AEs), grade 3–4 AEs, serious adverse events (SAEs), or treatment discontinuation due to AEs.

### Exclusion criteria

The network meta-analysis excluded studies that met any of the following criteria: studies not involving CDK4/6 inhibitors in combination with fulvestrant, duplicate publications, inaccessible full texts, non-original research (e.g., reviews, preclinical studies, case reports), or non-English publications. For multiple reports from the same RCT, only the most complete and recent version was included.

### Study selection

Two researchers independently screened the literature using NoteExpress software, following the established inclusion and exclusion criteria. Duplicate publications were identified and removed using the software’s duplicate-checking feature. The titles and abstracts of the remaining studies were then reviewed to exclude those that did not meet the eligibility criteria. Full-text assessments were subsequently conducted on studies that initially appeared eligible to confirm their inclusion in the network meta-analysis. Disagreements between the researchers were resolved through discussion, and if consensus could not be reached, a third researcher was consulted to achieve a final decision.

### Data extraction

Data extraction was also performed independently by the two researchers. The extracted data included: (1) basic study information such as the title, first author, clinical trial registration number, and publication year; (2) participant characteristics, including sample size and median age; (3) details of the intervention and control group treatment regimens; and (4) outcome measures, such as PFS, OS, ORR, CBR, QoL, incidence of AEs, grade 3–4 AEs, SAEs, and treatment discontinuation due to AEs.

### Methodological quality assessment

The risk of bias for the included RCTs was independently evaluated by two researchers using the Cochrane Handbook’s Risk of Bias (RoB) tool [15], focusing on five domains: the randomization process, including sequence generation and allocation concealment; deviations from intended interventions, assessing adherence and blinding; missing outcome data, evaluating the impact of incomplete data and handling methods; selective outcome reporting, identifying reporting bias; and outcome measurement, examining the validity and consistency of measurement methods. Each study was rated as having “low,” “some concerns,” or “high” risk of bias for each domain.

### Statistical analysis

Network meta-analysis was conducted using the gemtc package in Rstudio, which applies a Bayesian framework based on Markov Chain Monte Carlo (MCMC) simulation. Four Markov chains were run simultaneously, each with 50,000 iterations, a burn-in period of 20,000, and a thinning interval of 10, ensuring model convergence and stability. For time-to-event outcomes, such as OS and PFS, hazard ratios (HRs) with 95% credible intervals (CrIs) were used. Relative risks (RRs) were applied for binary outcomes, and mean differences (MDs) for continuous outcomes, all with 95% CrIs. Treatment efficacy and safety were ranked using the surface under the cumulative ranking curve (SUCRA), where higher values indicate greater probability of being the most effective or safest option [16]. Between-study heterogeneity was assessed using the between-trial variance τ², interpreted as follows: low (< 0.04), low–moderate (0.04–0.16), moderate–high (0.16–0.36), and high (> 0.36).

## Results

### Literature selection and study characteristics

**Fig. 1:**
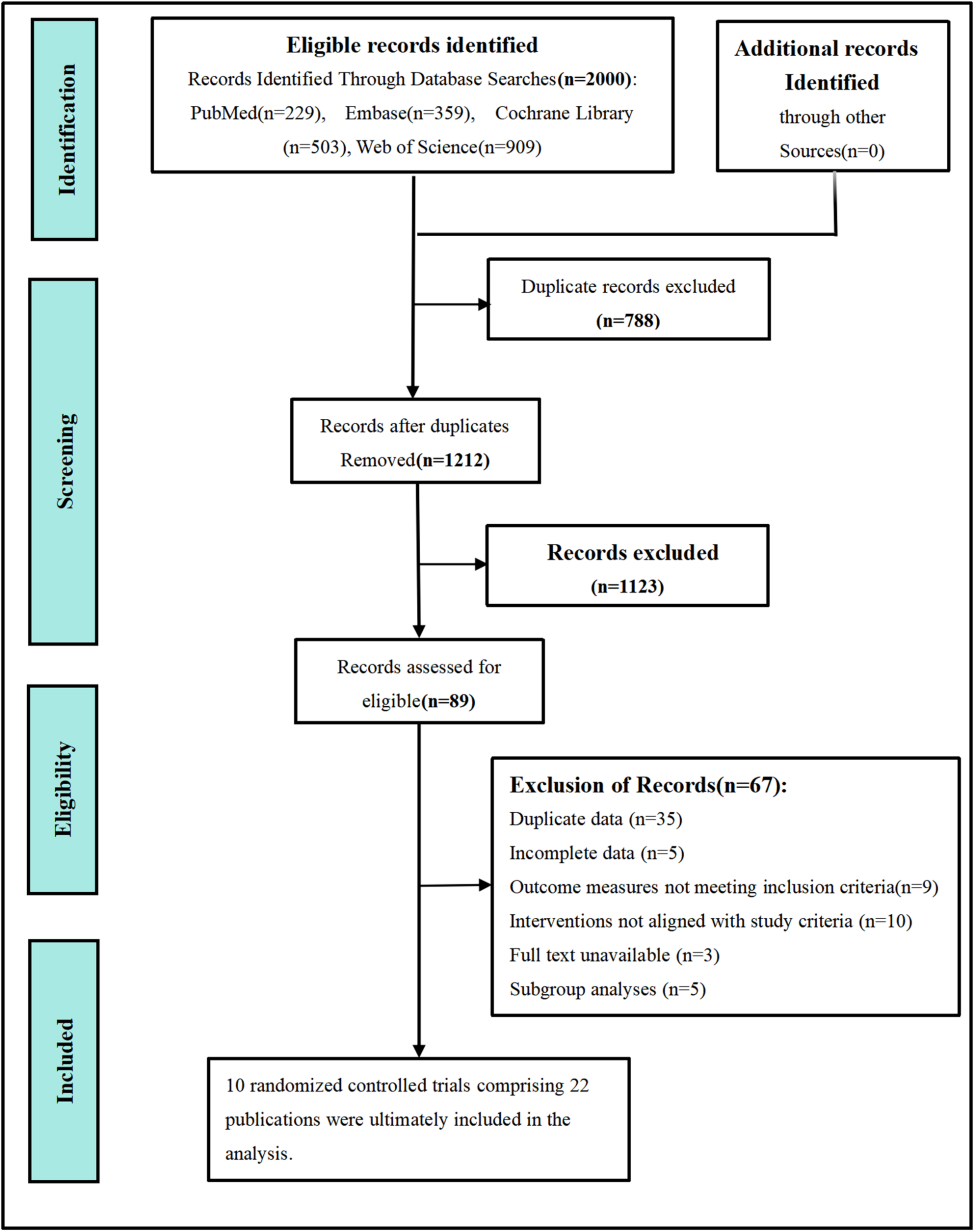
Flow diagram of preferred reporting items identified, included, and excluded for systematic reviews and meta-analyses (PRISMA)

A total of 2,000 studies were identified through the predefined search strategy. After removing 788 duplicate records using NoteExpress software, 1,123 studies were excluded based on title and abstract screening for eligibility. Full-text reviews were conducted on the remaining 89 studies, ultimately including 10 RCTs comprising 22 publications [17–38] in this network meta-analysis. These studies involved a total of 3,465 patients with HR+/HER2– advanced breast cancer. The literature screening process is detailed in Fig. 1, and the key characteristics of the included studies are summarized in Table 1.

**Table 1:** Baseline characteristics of included studies

Study	Study Design	Menstrual status	Sample Size		Age		Treatment Regimen		Outcome
			T	C	T	C	T	C	
PALOMA-3 [17–22] NCT01942135	Phase III, Double-Blind	both	347	174	57	56	PAL + FUL	PLA + FUL	1,2,3,4,5,6,7,8,9
MONARCH-2 [23–25] NCT02107703	Phase III, Double-Blind	both	446	223	59	62	ABE + FUL	PLA + FUL	1,2,3,4,5,6,7,8,9
MONALEESA-3 [26–30] NCT02422615	Phase III, Double-Blind	postmenopausal	237	109	63	63	RIB + FUL	PLA + FUL	1,2,3,4,5,8,9
DAWNA-1 [31] NCT03927456	Phase III, Double-Blind	both	241	120	51	52	DAL + FUL	PLA + FUL	1,3,4,7,8,9
MONARCH plus /Cohort B [32] NCT02763566	Phase III, Double-Blind	postmenopausal	104	53	60	60	ABE + FUL	PLA + FUL	1,3,4,6,7,8
FLIPPER [33, 34] NCT02690480	Phase II, Double-Blind	postmenopausal	94	95	64	64	PAL + FUL	PLA + FUL	1,3,4,5,6,7,8
BRIGHT-2 [35] NCT05077449	Phase III, Double-Blind	both	204	101	55	55	BIR + FUL	PLA + FUL	1,3,4,6,7,8
postMONARCH [36] NCT05169567	Phase III, Double-Blind	both	182	186	58	61	ABE + FUL	PLA + FUL	1,3,4,6,7,8,9
LEONARDA-1 [37] NCT05054751	Phase III, Double-Blind	both	137	138	NA	NA	LERO + FUL	PLA + FUL	1,3,4,6,7,8,9
TIFFANY [38] NCT05433480	Phase III, Double-Blind	both	184	90	NA	NA	TIB + FUL	PLA + FUL	1,3,4,6,7,8,9

(1) (*PFS*) Progression-free survival, (2) (*OS*) Overall survival, (3) (*ORR*) Objective response rate, (4) (*CBR*) Clinical benefit rate, (5) (*HRQoL*) Health-related quality of life, (6) (*AEs*) Incidence of all-grade adverse events, (7) Incidence of grade 3–4 AEs; (8) (*SAEs*) Serious adverse events , (9) Treatment discontinuation due to AEs. *T* Treatment group, *C* Control group, *PAL* Palbociclib, *RIB* Ribociclib, *ABE* Abemaciclib, *DAL* Dalpiciclib, *BIR* Bireociclib, *LERO* Lerociclib, *TIB* Tibremciclib, *PLA* Placebo, *FUL* Fulvestrant

### Quality assessment of included studies and consistency

**Fig. 2:**
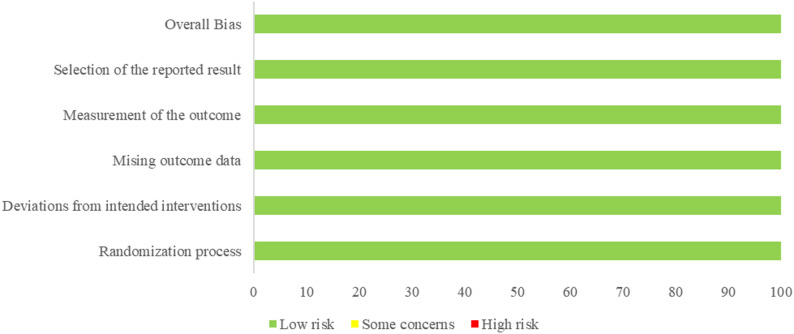
Risk of bias assessment for included studies

**Fig. 3:**
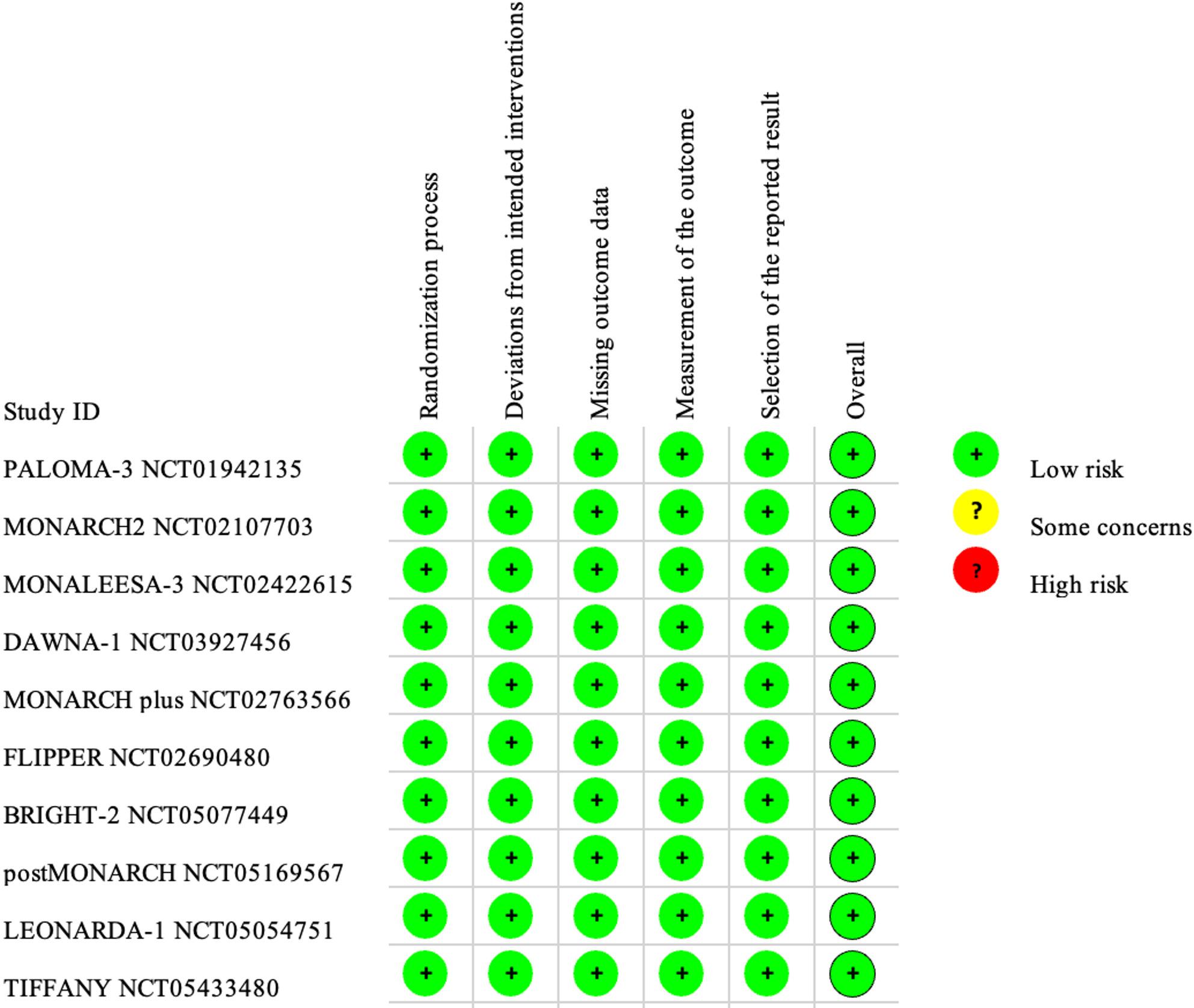
Risk of bias summary for included studies

The quality of the included studies was evaluated using the Cochrane Risk of Bias Assessment Tool, focusing on five domains: randomization process, deviations from intended interventions, missing outcome data, selective outcome reporting, and outcome measurement. The results of this assessment are summarized in Figs. 2 and 3. All studies documented random sequence generation and allocation concealment, ensuring minimal bias in these areas. Notably, no bias was detected across studies regarding outcome data completeness, selective reporting, or the measurement of outcomes. Our evaluation of the alignment between direct and indirect evidence showed strong consistency across all comparisons. Density plots, trace plots, and convergence diagnostic plots further supported this observation (Appendix 4 & 5 in Supplementary Material). The τ² results showed a few instances of significant heterogeneity within the network, though most comparisons exhibited low to moderate levels of heterogeneity (Appendix 3 in Supplementary Material).

### Meta-analysis of progression-free survival

**Fig. 4:**
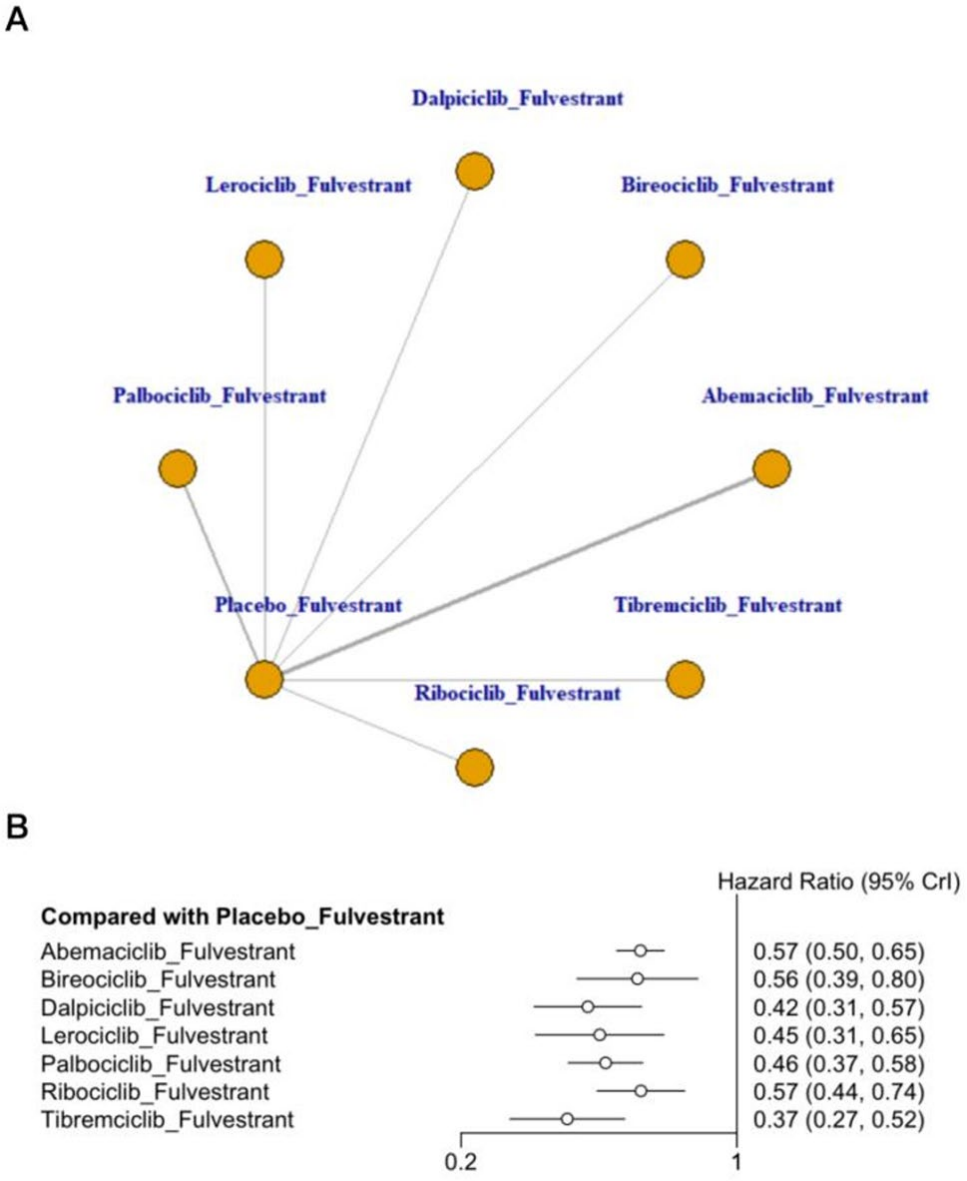
Network plot and forest plot of PFS. (**A**) Network plot. (**B**) Forest plot

This network meta-analysis synthesized data from ten RCTs evaluating seven fulvestrant-based treatment strategies in combination with CDK4/6 inhibitors. As shown in Fig. 4, all CDK4/6 inhibitors combined with fulvestrant demonstrated significant improvement in PFS compared with the placebo + fulvestrant group in patients with HR+/HER2- advanced or metastatic breast cancer. Tibremciclib + fulvestrant showed the most favorable effect estimate versus placebo + fulvestrant and ranked highest by SUCRA (HR = 0.37, 95% CrI 0.27–0.52; SUCRA = 89.26%), followed by dalpiciclib + fulvestrant (HR = 0.42, 95% CrI 0.31–0.57; SUCRA = 77.10%) and lerociclib + fulvestrant (HR = 0.45, 95% CrI 0.31–0.65; SUCRA = 67.26%)(Appendix 7, Fig. 7.1 in Supplementary Material). League table comparisons suggested that tibremciclib + fulvestrant was associated with longer PFS than ribociclib + fulvestrant and abemaciclib + fulvestrant, with the corresponding comparisons reported as HRs for ribociclib vs. tibremciclib (HR = 1.54, 95% CrI 1.01–2.34) and abemaciclib vs. tibremciclib (HR = 1.53, 95% CrI 1.07–2.20), while no other pairwise differences reached statistical significance (Appendix 8, Table S8.1 in Supplementary Material). Overall, these findings consistently indicate that CDK4/6 inhibitor—based combinations with fulvestrant provide superior PFS outcomes compared with placebo + fulvestrant.

### Meta-analysis of overall survival

**Fig. 5:**
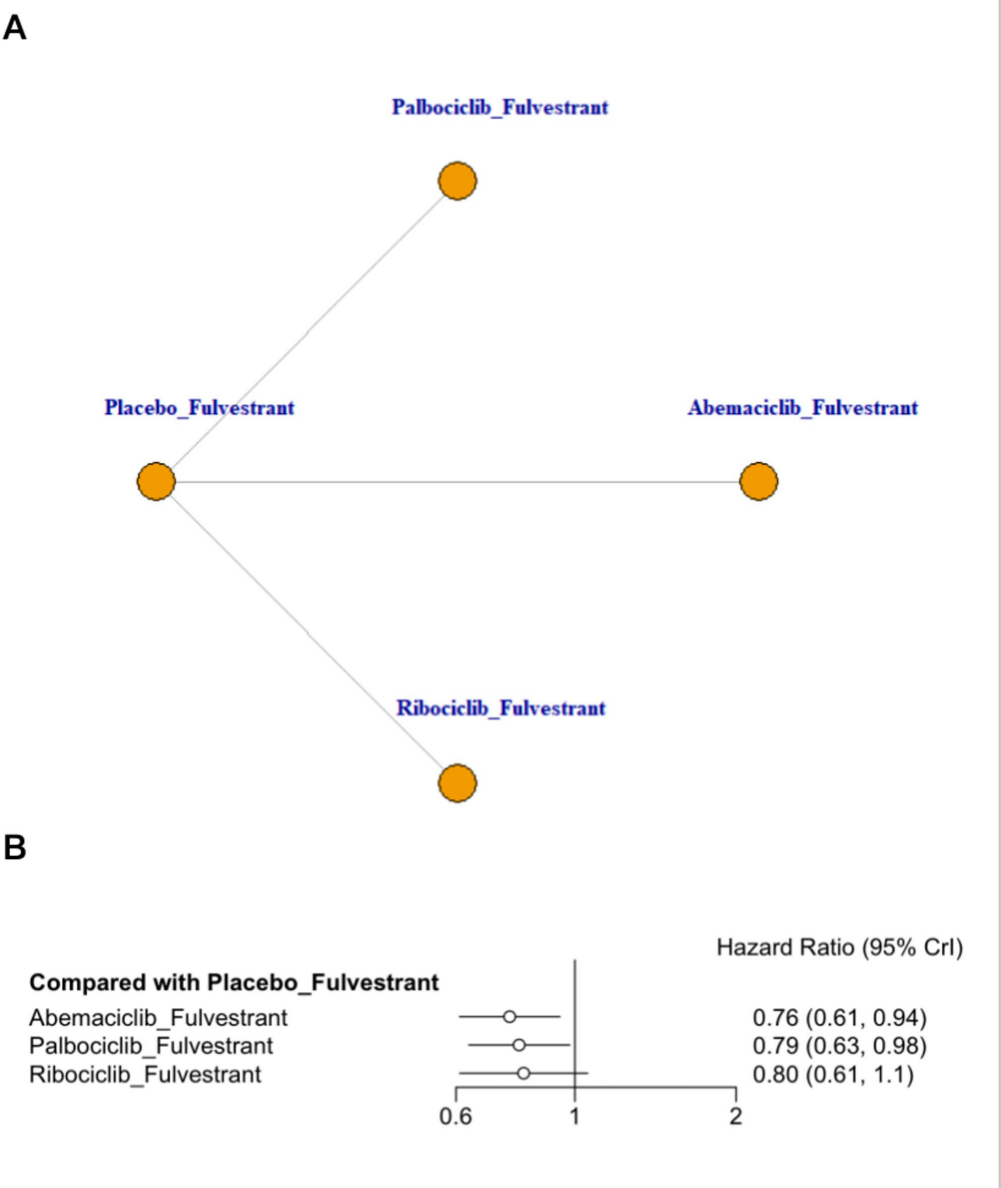
Network plot and forest plot of OS. (**A**) Network plot. (**B**) Forest plot

This network meta-analysis synthesized data from three RCTs evaluating fulvestrant-based backbones in combination with CDK4/6 inhibitors. As shown in Fig. 5B, all CDK4/6 inhibitor plus fulvestrant regimens yielded numerically favorable OS estimates versus placebo + fulvestrant. Abemaciclib + fulvestrant showed the most favorable point estimate and a statistically supported OS benefit within the network model (HR = 0.76, 95% CrI 0.61–0.94; SUCRA = 74.24%), followed by palbociclib + fulvestrant (HR = 0.79, 95% CrI 0.63–0.98; SUCRA = 64.26%). Ribociclib + fulvestrant showed a numerically favorable effect but did not reach statistical significance in the network model (SUCRA = 58.87%). Given that MONALEESA-3 reported a statistically significant OS benefit for ribociclib versus placebo, the network estimate should be interpreted cautiously and in conjunction with the corresponding direct trial evidence. Indirect comparisons from the league table revealed no statistically significant differences among the CDK4/6 inhibitor regimens (Appendix 8, Table S8.2 in Supplementary Material).

### Meta-analysis of objective response rate and clinical benefit rate

**Fig. 6:**
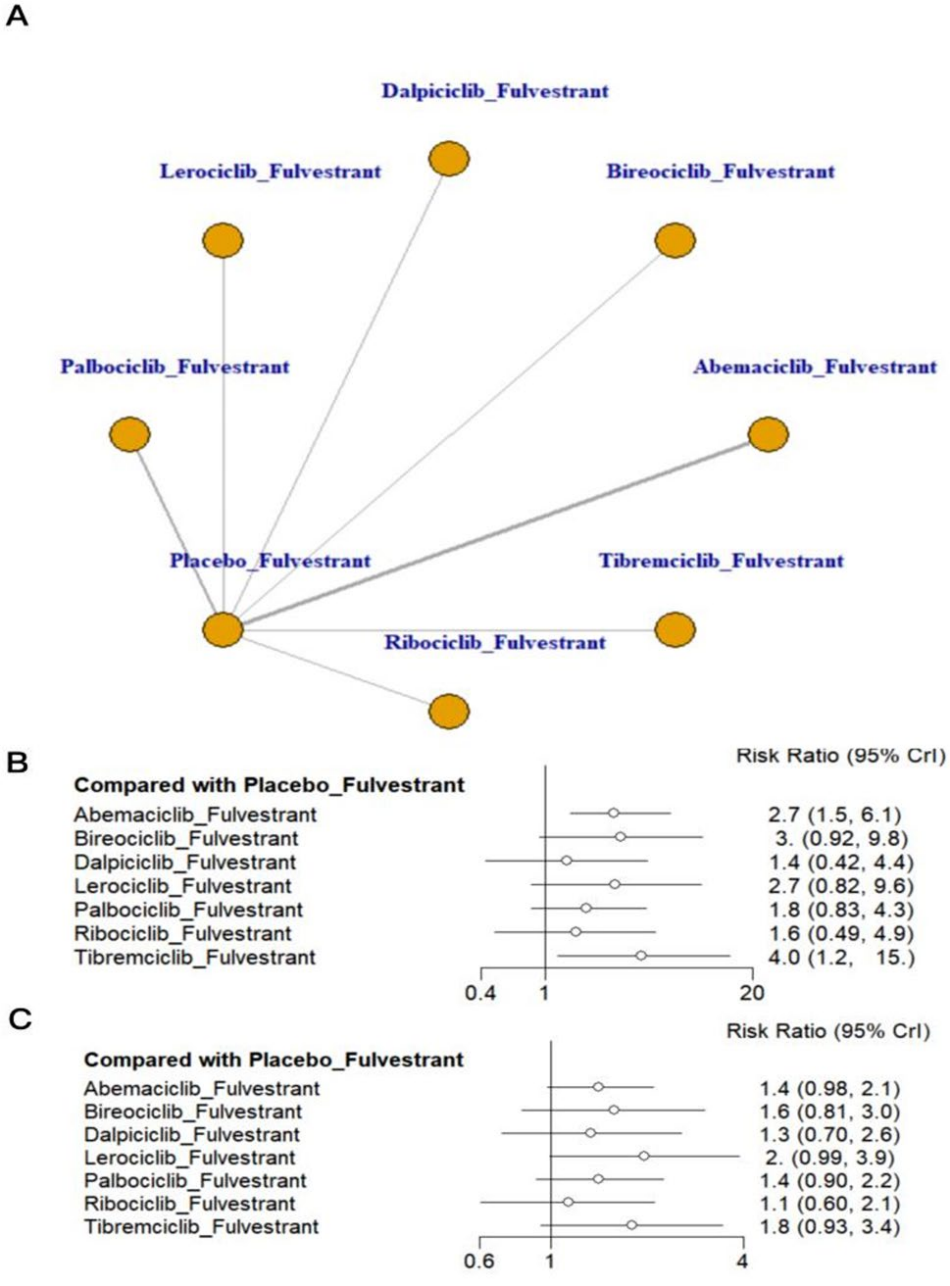
Network plot and forest plot of ORR and CBR. (**A**) Network plot. (**B**) Forest plot of ORR. (**C**) Forest plot of CBR

This network meta-analysis synthesized data from 10 RCTs evaluating seven CDK4/6 inhibitor–based treatment strategies combined with fulvestrant. Figure 6 presents the network diagram and forest plots for ORR and CBR comparisons. Figure 6B shows the forest plot for ORR. All treatment regimens yielded numerically higher ORR estimates compared with placebo + fulvestrant. Tibremciclib + fulvestrant ranked highest for ORR and was associated with a statistically supported improvement versus placebo within the network model (RR = 4.0, 95% CrI 1.2–15.0; SUCRA = 84.27%), followed by abemaciclib + fulvestrant (RR = 2.7, 95% CrI 1.5–6.1; SUCRA = 68.13%)(Appendix 7, Fig. 7.3 in Supplementary Material). Figure 6C shows the forest plot for CBR. All regimens showed numerically favorable CBR estimates versus placebo + fulvestrant, but none of the comparisons reached statistical significance. Additionally, league table comparisons for both ORR and CBR did not reveal any statistically significant differences among treatment regimens (Appendix 8, Tables S8.3 and S8.4 in Supplementary Material).

### Meta-analysis of quality of life

**Fig. 7:**
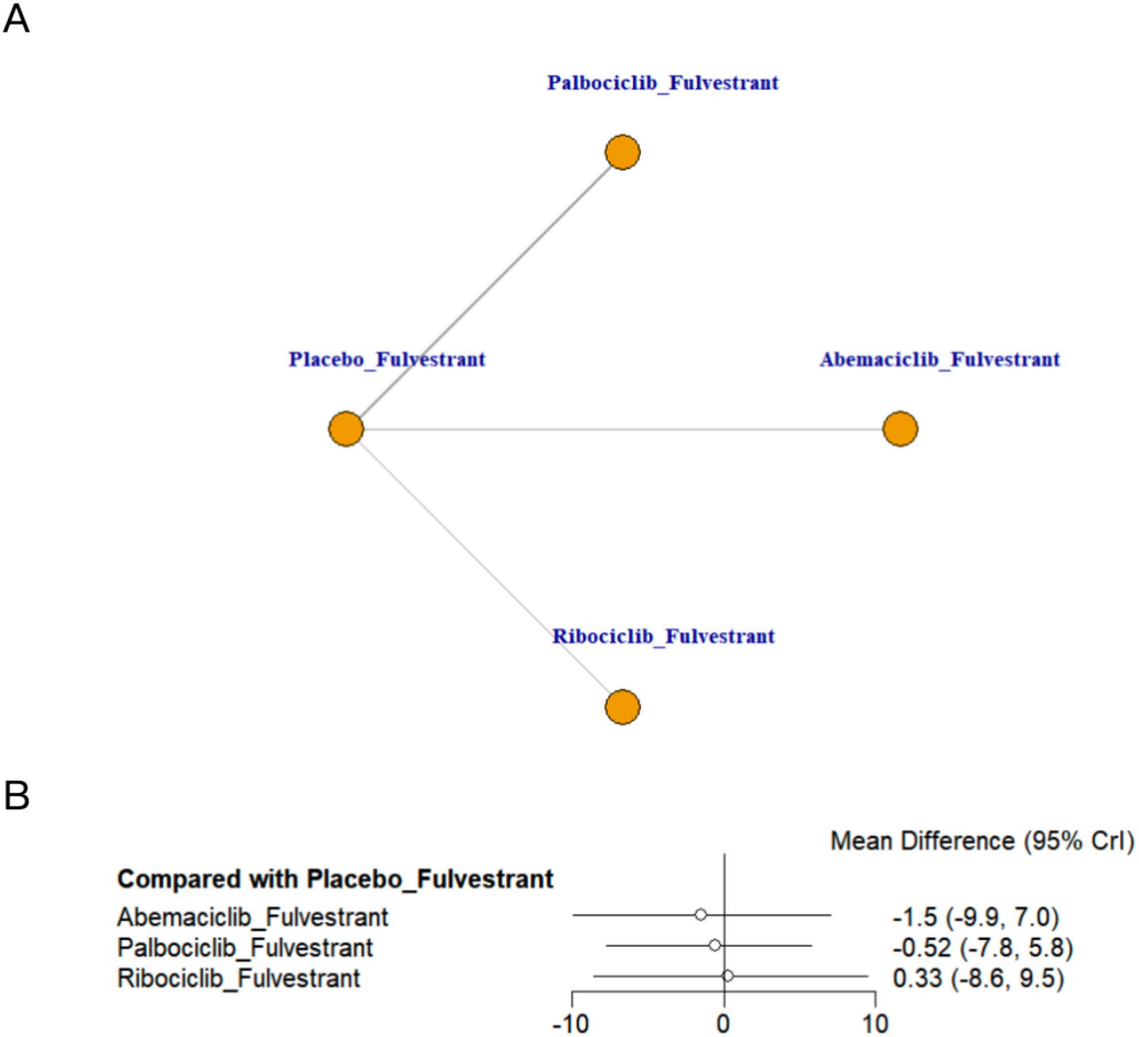
Network plot and forest plot of QoL. (**A**) Network plot. (**B**) Forest plot

This study included data from four randomized controlled trials evaluating four different treatment strategies for QoL. Figure 7 presents the network diagram and forest plot for these comparisons. According to the forest plot, none of the treatment regimens showed a statistically significant improvement in QoL compared with placebo + fulvestrant. Although ribociclib + fulvestrant ranked highest based on SUCRA, the QoL findings should be interpreted cautiously given the lack of statistical significance and the limited evidence base (Appendix 7, Fig. 7.5 in Supplementary Material). Indirect comparisons from the league table revealed no statistically significant differences among the various CDK4/6 inhibitor + fulvestrant regimens (Appendix 8, Table S8.5 in Supplementary Material).

### Meta-analysis of adverse events

**Fig. 8:**
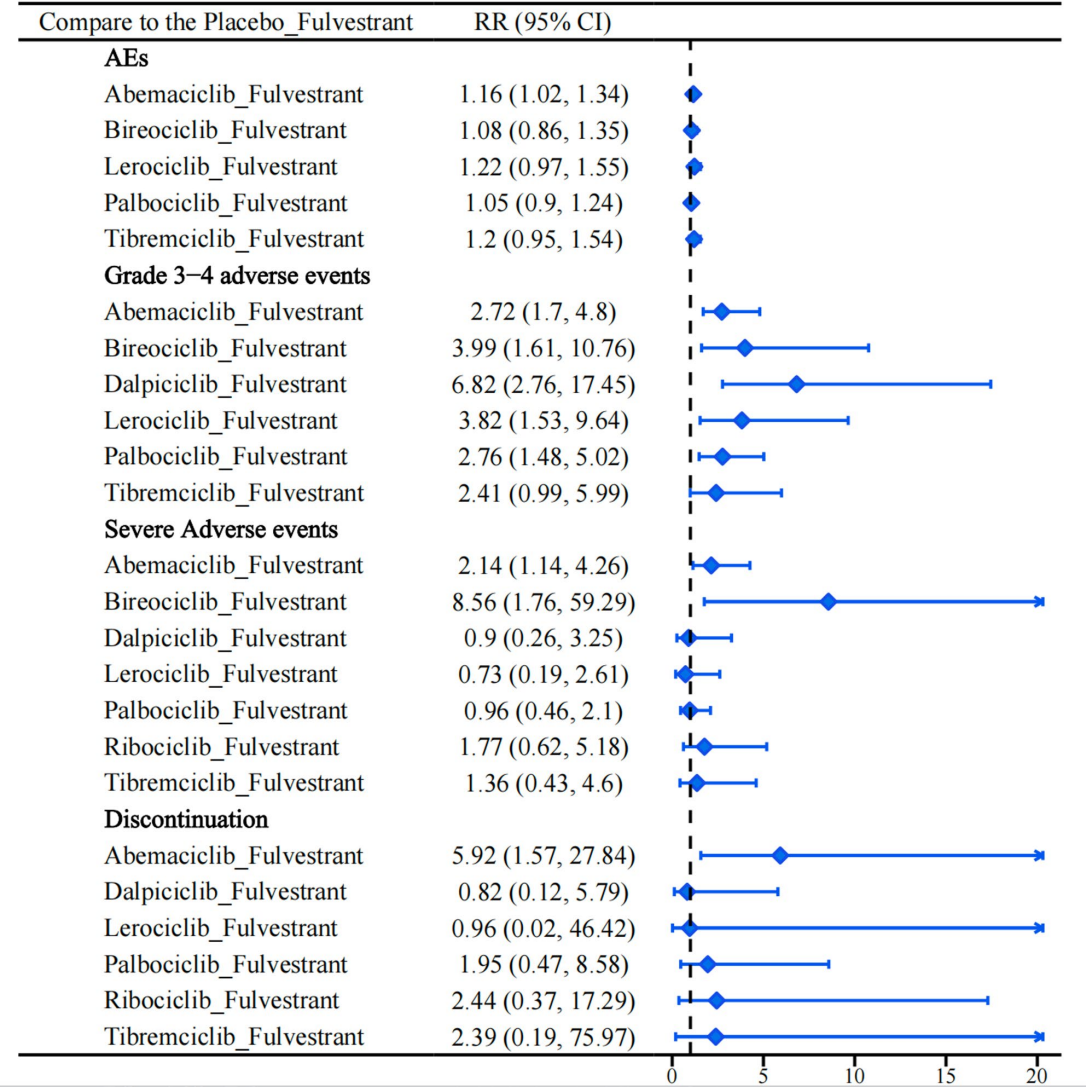
Forest plot of Adverse Events (AEs), grade 3–4 adverse events, Severe Adverse Events (SAEs), and treatment discontinuation

All treatment regimens showed numerically higher risks of AEs compared with placebo + fulvestrant, with abemaciclib + fulvestrant (RR = 1.16, 95% CrI 1.02–1.34) showing a statistically significant increase (Fig. 8). For grade 3–4 AEs, dalpiciclib + fulvestrant (RR = 6.82, 95% CrI 2.76–17.45), bireociclib + fulvestrant (RR = 3.99, 95% CrI 1.61–10.76), and lerociclib + fulvestrant (RR = 3.82, 95% CrI 1.53–6.94) were associated with higher estimated risks (Fig. 8). Regarding SAEs, bireociclib + fulvestrant (RR = 8.56, 95% CrI 1.76–59.29) and abemaciclib + fulvestrant (RR = 2.14, 95% CrI 1.14–4.26) showed higher estimated risks, although the estimates were imprecise for some comparisons (Fig. 8). Treatment discontinuation due to AEs was higher with abemaciclib + fulvestrant (RR = 5.92, 95% CrI 1.57–27.84) (Fig. 8). However, statistically significant between-regimen differences were observed only in the league table for SAEs, where bireociclib + fulvestrant showed higher risks than dalpiciclib + fulvestrant, lerociclib + fulvestrant, and palbociclib + fulvestrant (RR = 9.58 [1.19–92.14], 11.92 [1.48–121.85], and 8.95 [1.55–70.12], respectively) (Appendix 8, Table S8.8 in Supplementary Material). No statistically significant differences were found among regimens for other adverse event outcomes(Appendix 8, Tables S8.6, S8.7, and S8.9 in Supplementary Material).

## Discussion

### Principal findings

This network meta-analysis assessed the efficacy and safety of seven CDK4/6 inhibitors combined with fulvestrant for HR+/HER2 − advanced breast cancer. All regimens were associated with statistically significant improvements in PFS versus placebo + fulvestrant, with tibremciclib + fulvestrant showing the most favorable point estimate and ranking highest by SUCRA. For OS, the network model yielded numerically favorable estimates across regimens; abemaciclib + fulvestrant and palbociclib + fulvestrant showed statistically supported improvements versus placebo within the network model, while indirect comparisons did not reveal statistically significant differences among CDK4/6 inhibitors. For ORR, tibremciclib + fulvestrant ranked highest, whereas CBR and QoL did not show statistically significant improvements versus placebo + fulvestrant. In safety analyses, abemaciclib + fulvestrant was the only regimen associated with a statistically significant increase in overall AEs. Dalpiciclib + fulvestrant, bireociclib + fulvestrant, and lerociclib + fulvestrant showed higher estimated risks of grade 3–4 AEs and SAEs, and treatment discontinuation due to AEs was higher with abemaciclib + fulvestrant.

In summary, while all CDK4/6 inhibitors combined with fulvestrant showed efficacy in improving PFS and some demonstrated favorable OS, safety concerns, particularly with abemaciclib + fulvestrant, warrant careful consideration. Further research is needed to refine treatment choices based on both efficacy and safety profiles.

### Comparison with previous studies

#### Confirmation of class-wide survival benefits

The primary finding of this study is that all seven CDK4/6 inhibitors combined with fulvestrant significantly improved PFS compared with fulvestrant monotherapy. This result aligns with the existing body of evidence, confirming that the substantial clinical benefits observed with earlier CDK4/6 inhibitors extend to newer agents within this class [39]. Several previous meta-analyses have firmly established this principle. For instance, Li et al. and another independent analysis both demonstrated that adding a CDK4/6 inhibitor to endocrine therapy significantly prolonged PFS (HR = 0.55 and 0.54, respectively) and OS (HR = 0.75 and 0.77, respectively) [39]. Similarly, a comprehensive systematic review concluded that these combinations improve OS, PFS, PFS2 (time to progression on subsequent therapy or death), and TTC (time to chemotherapy) [40]. Mechanistically, CDK4/6 inhibitors prevent phosphorylation of the retinoblastoma (Rb) protein, a critical downstream effector of estrogen receptor signaling, thereby inducing G1-phase cell-cycle arrest [41]. Fulvestrant, a selective estrogen receptor degrader (SERD), complements this mechanism by disrupting estrogen receptor–mediated transcription, delaying endocrine resistance [42]. The consistency of our findings across multiple agents underscores that this synergistic mechanism remains the cornerstone of therapeutic benefit within the CDK4/6 inhibitor class.

#### New insights into comparative efficacy among agents

While the overall benefit of CDK4/6 inhibitors is well established, a key contribution of this analysis is to provide an updated comparative profile that incorporates newer agents that were largely absent from prior network meta-analyses. In our network estimates, tibremciclib + fulvestrant showed the most favorable effect estimates versus placebo + fulvestrant for both PFS and ORR and ranked highest by SUCRA for these outcomes. Importantly, these findings are consistent with direct phase III evidence from the TIFFANY trial, in which tibremciclib + fulvestrant improved median PFS (16.5 vs. 5.6 months; HR 0.37; 95% CI, 0.27–0.52) and increased ORR (45.6% vs. 12.9%) compared with placebo + fulvestrant [38]. Overall, the effect size observed with tibremciclib + fulvestrant appears large within this evidence base.

This finding contrasts with earlier network meta-analyses that, due to the absence of lerociclib, bireociclib, and tibremciclib data, proposed different efficacy hierarchies. For instance, some analyses suggested abemaciclib + AI as the most effective regimen for PFS [43], while others found dalpiciclib + ET superior for PFS and ribociclib + ET for OS [44]. These shifts in ranking are not contradictory but reflect the field’s maturation and the evolution of available evidence. Early analyses were restricted to three agents—palbociclib, ribociclib, and abemaciclib—and often combined AI- and fulvestrant-based backbones, limiting clinical specificity. The later inclusion of dalpiciclib changed the relative rankings [44]. Our study is the first to integrate lerociclib, bireociclib, and tibremciclib within a fulvestrant-specific network, providing a more contemporary and clinically focused comparison. This underscores a central principle of network meta-analysis: the findings depend heavily on the included evidence base and the precise clinical question being addressed.

Our analysis also contextualizes the efficacy of other emerging CDK4/6 inhibitors. In the DAWNA-1 trial, dalpiciclib demonstrated robust efficacy with a median PFS of 15.7 months (HR 0.42) [31]. Similarly, the LEONARDA-1 trial confirmed the activity of lerociclib, though its median PFS (11.07 months; HR 0.45) was comparatively moderate [37]. By integrating these newer agents, our study provides clinicians with a more complete and nuanced comparative framework when considering treatment options beyond the three most established CDK4/6 inhibitors.

#### Analysis of divergent findings: clinical benefit rate and quality of life

In the present study, CDK4/6 inhibitor combinations showed no statistically significant improvements in CBR or QoL versus placebo plus fulvestrant, conflicting with results of several prior meta-analyses. A plausible explanation for the CBR discrepancy is the inclusion of more agents and a broader toxicity profile in our study; a high incidence of grade ≥ 3 adverse events may have caused treatment interruptions, dose reductions, or early discontinuation, preventing patients from meeting the CBR threshold of stable disease for at least 24 weeks and thus statistically reducing the detectability of overall clinical benefit.

In contrast, QoL analysis only included three widely used CDK4/6 inhibitors, making methodological differences the more likely cause: inconsistent QoL scales and assessment time points across trials, high missing data rates with variable handling (e.g., different imputation strategies), and imprecise estimates from limited evidence collectively drove the pooled effect toward neutrality. Collectively, PFS is a more objective and consistent endpoint that demonstrated robust benefits, whereas CBR and QoL were more sensitive to toxicity burden, endpoint definitions and measurement methodologies. This indicates that treatment selection requires a more nuanced trade-off between efficacy and tolerability.

#### Comparative assessment of safety and tolerability

The safety outcomes in our network meta-analysis largely align with the known toxicity profiles reported in individual clinical trials, while also emphasizing meaningful differences across agents. Abemaciclib plus fulvestrant was the only regimen significantly associated with an increase in overall adverse events (AEs) and was associated with a higher rate of treatment discontinuation due to toxicity. This pattern reflects its distinct pharmacologic profile—unlike other agents typically given intermittently, abemaciclib is administered continuously [41], which may contribute to persistent low-grade toxicities such as diarrhea. Although these events are generally manageable, their cumulative burden over time likely explains the higher discontinuation rate.

Our results also identified strong toxicity signals for several newer agents. Dalpiciclib, bireociclib, and lerociclib were associated with higher risks of grade 3–4 AEs and/or serious adverse events (SAEs), findings consistent with their pivotal trials. In the DAWNA-1 trial, dalpiciclib caused grade 3–4 neutropenia in 84.2% and leukopenia in 62.1% of patients [31], confirming its pronounced hematologic toxicity. Similarly, the phase III BRIGHT-2 trial showed that bireociclib plus fulvestrant led to grade ≥ 3 AEs in 64.7% of patients—substantially higher than 18.8% in the placebo group—with neutropenia, leukopenia, and anemia being the most common events. These data validate the increased SAEs risk observed in our analysis and define the hematologic nature of bireociclib’s toxicity. In the LEONARDA-1 trial, 57.7% of patients receiving lerociclib experienced grade ≥ 3 AEs [37], supporting our findings. Interestingly, some reports have described lerociclib as potentially more tolerable, given its lower incidence of grade 4 neutropenia and gastrointestinal events—attributes that may enable a continuous dosing schedule without drug holidays [45]. Such nuances suggest that while the overall frequency of high-grade AEs may be comparable, the specific toxicity patterns differ among agents, warranting further investigation in head-to-head trials. The safety profiles of palbociclib and ribociclib are well established, characterized primarily by manageable neutropenia, with ribociclib additionally requiring QTc monitoring. Our analysis confirmed that these agents occupy a distinct position in the safety spectrum compared with newer drugs that exhibit greater myelosuppression or unique toxicities.

In summary, this network meta-analysis reaffirms the central role of CDK4/6 inhibitors in improving PFS among patients with HR+/HER2 − advanced breast cancer, while providing an updated perspective on efficacy ranking and safety differentiation. Tibremciclib emerged as a highly efficacious option, but the findings also highlight the delicate balance between efficacy and tolerability. The pronounced toxicities observed with some newer agents may attenuate class-level benefits in CBR and QoL, underscoring the importance of individualized treatment selection based on both therapeutic potential and safety profile.

### Strengths and limitations

This study is, to our knowledge, among the most comprehensive Bayesian network meta-analyses focused specifically on fulvestrant-based combinations with all seven approved CDK4/6 inhibitors in HR+/HER2 − advanced breast cancer. By integrating direct and indirect trial-level evidence, it provides a quantitative synthesis that complements, rather than replaces, translational, biomarker-informed, and real-world perspectives. The inclusion of newer agents (e.g., lerociclib, bireociclib, tibremciclib) extends the comparative landscape beyond earlier meta-analyses and helps contextualize class-level benefits with regimen-specific safety profiles.

Several limitations warrant consideration. First, variability in trial populations (line of therapy, prior endocrine exposure, geographic mix) and assessment schedules introduces clinical and methodological heterogeneity that may influence relative effects. Second, the network is confined to fulvestrant-based backbones; while this improves biological homogeneity, it limits generalizability to AI-based combinations and precludes cross-backbone inferences. Third, although tibremciclib is now represented by a peer-reviewed randomized trial, current estimates still rely on a single pivotal study with its own follow-up maturity and design features; additional independent trials may shift rankings. Fourth, overall survival estimates from the network should be interpreted cautiously, as they did not fully mirror statistically significant OS benefits reported in some key trials (e.g., MONARCH-2 and MONALEESA-3). Notably, for MONALEESA-3 we included the latest peer-reviewed OS analysis; thus, the discrepancy is unlikely to stem from outdated cut-offs or insufficient follow-up for that trial. More plausible contributors include the evidence structure and modelling features of the NMA (e.g., sparse geometry with limited head-to-head evidence, reliance on indirect comparisons, and aggregate-level time-to-event summaries), together with key assumptions such as exchangeability and proportional hazards, which may attenuate trial-level signals. Accordingly, OS results from the network should be interpreted cautiously and in conjunction with the corresponding trial evidence. Fifth, safety comparisons were constrained by incomplete exposure-adjusted reporting and differences in dose intensity/interruptions across trials, which may bias between-study contrasts. Sixth, QoL instruments, timepoints, and missing-data handling varied, and CBR definitions and assessment frequency were not fully uniform, limiting cross-trial comparability for these endpoints. Finally, the network structure provided limited closed loops, restricting formal consistency checks; Bayesian inferences remain sensitive to model specification (fixed vs. random effects), prior choices, MCMC convergence, and ranking uncertainty.

In summary, this network meta-analysis consolidates trial-based efficacy and safety evidence for fulvestrant-based CDK4/6 inhibitor combinations and should be interpreted as complementary to mechanistic, biomarker-guided, and real-world investigations. Further head-to-head trials, longer follow-up, standardized exposure-adjusted safety reporting, and integration of biomarker data are needed to refine treatment sequencing and improve clinical applicability.

### Clinical implications, challenges, and future directions

This network meta-analysis provides one of the most comprehensive evidence bases to guide clinicians in selecting among various CDK4/6 inhibitors combined with fulvestrant. Our findings clarify the relative efficacy and safety of available regimens, highlighting the promising potential of newer agents such as tibremciclib. However, real-world decision-making remains more complex than controlled trial settings, particularly regarding treatment sequencing after progression on CDK4/6 inhibitors—a key clinical challenge.

The primary value of this study lies in optimizing initial or subsequent therapeutic choices, underscoring that outcomes after CDK4/6 inhibitor failure are generally poor. Our ranking analysis helps identify the regimens most likely to extend PFS, while the real-world study by Molinelli et al. [10] complements these findings. In 701 patients from the GIM14/BIOMETA study, post-CDK4/6 inhibitor treatments—such as capecitabine, taxanes, or everolimus plus exemestane—showed similar median treatment durations of only 5–6 months. Notably, patients with shorter CDK4/6 inhibitor exposure were more likely to receive chemotherapy as subsequent therapy. Together, these data suggest that maximizing benefit from the first CDK4/6 inhibitor regimen is crucial, as options after progression remain limited and less effective.

In the post-CDK4/6 inhibitor era, biomarker-guided strategies underpin resistance management and sequential therapy optimization for HR+/HER2– advanced breast cancer. Our study’s focus on regimens’ “average” efficacy (excluding patient-specific responses) is offset by circulating tumor DNA (ctDNA)-based monitoring, an essential tool for dynamic resistance profiling and precise stratification beyond static trial/meta-data. Gerratana et al. [11] verified ctDNA-guided therapy: sustained CDK4/6 inhibition with a novel endocrine partner improved PFS/OS in ESR1-mutated patients (endocrine-driven resistance, supporting AI-to-fulvestrant switching). RB1-mutated patients had no benefit, reflecting bona fide CDK4/6 resistance and ctDNA’s mechanistic differentiation value. ESR1-m, the leading acquired resistance to first-line AI + CDK4/6i via constitutive ER activation, is ctDNA-detectable during first-line therapy. This was validated by the phase 3 SERENA-6 trial [46], the first global ctDNA-guided pre-progression switching study. Among 3,256 monitored patients, 315 ESR1-m-positive/progression-free patients randomized to camizestrant (next-generation SERD) + continued CDK4/6i achieved significant PFS benefit (HR = 0.44, 95% CrI 0.31–0.60, *p* < 0.00001; median 16.0 vs. 9.2 months) with favorable tolerability (AEs discontinuation: 1.3% vs. 1.9%). Foffano et al. [47] expanded ctDNA applications via ddPCR-based fragmentomics; elevated ACTB_short fragments correlate with adverse prognosis and inferior OS. This cost-effective approach supplements meta-analyses with continuous monitoring, and integrating longitudinal ctDNA tools with efficacy data forms a precision loop for tailored therapy. Unifying static efficacy data and dynamic monitoring, ctDNA serves as a core component of personalized advanced breast cancer management, optimizing first-line and sequential therapy in the post-CDK4/6 inhibitor era.

## Conclusions

This Bayesian network meta-analysis demonstrates that all CDK4/6 inhibitors combined with fulvestrant significantly improve progression-free survival in patients with HR+/HER2 − advanced breast cancer, with some regimens also showing overall survival benefits.Among these, tibremciclib plus fulvestrant demonstrated relatively pronounced efficacy, while abemaciclib plus fulvestrant was associated with a higher incidence of treatment-related adverse events. These findings confirm the class effect of CDK4/6 inhibition and highlight clinically meaningful differences among agents. Careful balance between efficacy and tolerability should guide therapeutic selection. Further prospective and biomarker-informed studies are warranted to optimize treatment sequencing and enhance personalized management strategies in this population.

## Supplementary Information

Supplementary Material 1.

## Data Availability

Enquiries regarding data availability should be directed to the corresponding authors. The data are not publicly available by default but can be shared upon reasonable request. Most of the data used are publicly accessible from previously published studies included in the systematic review.
