# Supplementary material for: CDK4/6 inhibitors combined with fulvestrant for the treatment of HR+/HER2–advanced or metastatic breast cancer: a Bayesian network meta-analysis
Source: BMC Cancer. 2026 Feb 17;26:402. doi: 10.1186/s12885-026-15713-z (PMC13020245; doi:10.1186/s12885-026-15713-z)
Supplement: Supplementary file 1 — Supplementary Material 1. [file 12885_2026_15713_MOESM1_ESM.docx]

**Supplementary Appendix**

**CDK4/6 Inhibitors Combined with Fulvestrant for the Treatment of HR+/HER2– Advanced or Metastatic Breast Cancer: A Bayesian Network Meta-Analysis**

**Table of contents**

*Appendix 1: PRISMA-NMA checklist................................................................................................................2*

*Appendix 2: Search strategy................................................................................................................................5*

*Appendix 3: Evaluation of inconsistency and heterogeneity ............................................................................9*

*Appendix 4: Density plots and Trajectory plots of comparisons of each outcome...........................................10*

*Appendix 5:Convergence Diagnostic Plot of comparisons of each outcome..................................................19*

*Appendix 6: Network maps of safety outcomes................................................................................................24*

*Appendix 7: SUCRA and cumulative probability plots....................................................................................28*

*Appendix 8: league table of Summary Estimates for CDK4/6 inhibitors of 10 Trials.....................................37*

**Appendix 1: PRISMA NMA Checklist**

| **Section/Topic** | **Item #** | **Checklist Item** | **Reported on Page #** |
| --- | --- | --- | --- |
| **TITLE** |  |  |  |
| Title | 1 | Identify the report as a systematic review *incorporating a network meta-analysis (or related form of meta-analysis).* | 1 |
|  |  |  |  |
| **ABSTRACT** |  |  |  |
| Structured summary | 2 | Provide a structured summary including, as applicable:  **Background:** main objectives  **Methods:** data sources; study eligibility criteria, participants, and interventions; study appraisal; and *synthesis methods, such as network meta-analysis.*  **Results:** number of studies and participants identified; summary estimates with corresponding confidence/credible intervals; *treatment rankings may also be discussed. Authors may choose to summarize pairwise comparisons against a chosen treatment included in their analyses for brevity.*  **Discussion/Conclusions:** limitations; conclusions and implications of findings.  **Other:** primary source of funding; systematic review registration number with registry name. | 2 |
|  |  |  |  |
| **INTRODUCTION** |  |  |  |
| Rationale | 3 | Describe the rationale for the review in the context of what is already known*, including mention of why a network meta-analysis has been conducted.* | ***3*** |
| Objectives | 4 | Provide an explicit statement of questions being addressed, with reference to participants, interventions, comparisons, outcomes, and study design (PICOS). | 4 |
|  |  |  |  |
| **METHODS** |  |  |  |
| Protocol and registration | 5 | Indicate whether a review protocol exists and if and where it can be accessed (e.g., Web address); and, if available, provide registration information, including registration number. | 4 |
| Eligibility criteria | 6 | Specify study characteristics (e.g., PICOS, length of follow-up) and report characteristics (e.g., years considered, language, publication status) used as criteria for eligibility, giving rationale. *Clearly describe eligible treatments included in the treatment network, and note whether any have been clustered or merged into the same node (with justification).* | ***5*** |
| Information sources | 7 | Describe all information sources (e.g., databases with dates of coverage, contact with study authors to identify additional studies) in the search and date last searched. | 4 |
| Search | 8 | Present full electronic search strategy for at least one database, including any limits used, such that it could be repeated. | 4 |
| Study selection | 9 | State the process for selecting studies (i.e., screening, eligibility, included in systematic review, and, if applicable, included in the meta-analysis). | 5 |
| Data collection process | 10 | Describe method of data extraction from reports (e.g., piloted forms, independently, in duplicate) and any processes for obtaining and confirming data from investigators. | 6 |
| Data items | 11 | List and define all variables for which data were sought (e.g., PICOS, funding sources) and any assumptions and simplifications made. | 6 |
| **Geometry of the network** | **S1** | Describe methods used to explore the geometry of the treatment network under study and potential biases related to it. This should include how the evidence base has been graphically summarized for presentation, and what characteristics were compiled and used to describe the evidence base to readers. | ***6*** |
| Risk of bias within individual studies | 12 | Describe methods used for assessing risk of bias of individual studies (including specification of whether this was done at the study or outcome level), and how this information is to be used in any data synthesis. | 6 |
| Summary measures | 13 | State the principal summary measures (e.g., risk ratio, difference in means). *Also describe the use of additional summary measures assessed, such as treatment rankings and surface under the cumulative ranking curve (SUCRA) values, as well as modified approaches used to present summary findings from meta-analyses.* | 7 |
| Planned methods of analysis | 14 | Describe the methods of handling data and combining results of studies for each network meta-analysis. This should include, but not be limited to:   - *Handling of multi-arm trials;* - *Selection of variance structure;* - *Selection of prior distributions in Bayesian analyses; and* - *Assessment of model fit.* | 7 |
| **Assessment of Inconsistency** | **S2** | Describe the statistical methods used to evaluate the agreement of direct and indirect evidence in the treatment network(s) studied. Describe efforts taken to address its presence when found. | 7 |
| Risk of bias across studies | 15 | Specify any assessment of risk of bias that may affect the cumulative evidence (e.g., publication bias, selective reporting within studies). | **7** |
| Additional analyses | 16 | Describe methods of additional analyses if done, indicating which were pre-specified. This may include, but not be limited to, the following:   - Sensitivity or subgroup analyses; - Meta-regression analyses; - *Alternative formulations of the treatment network; and* - *Use of alternative prior distributions for Bayesian analyses (if applicable).* | ***7*** |
| **RESULTS†** |  |  |  |
| Study selection | 17 | Give numbers of studies screened, assessed for eligibility, and included in the review, with reasons for exclusions at each stage, ideally with a flow diagram. | 7 |
| **Presentation of network structure** | **S3** | Provide a network graph of the included studies to enable visualization of the geometry of the treatment network. | ***10*** |
| **Summary of network geometry** | **S4** | Provide a brief overview of characteristics of the treatment network. This may include commentary on the abundance of trials and randomized patients for the different interventions and pairwise comparisons in the network, gaps of evidence in the treatment network, and potential biases reflected by the network structure. | ***10*** |
| Study characteristics | 18 | For each study, present characteristics for which data were extracted (e.g., study size, PICOS, follow-up period) and provide the citations. | 9 |
| Risk of bias within studies | 19 | Present data on risk of bias of each study and, if available, any outcome level assessment. | 9 |
| Results of individual studies | 20 | For all outcomes considered (benefits or harms), present, for each study: 1) simple summary data for each intervention group, and 2) effect estimates and confidence intervals. *Modified approaches may be needed to deal with information from larger networks.* | ***8*** |
| Synthesis of results | 21 | Present results of each meta-analysis done, including confidence/credible intervals. *In larger networks, authors may focus on comparisons versus a particular comparator (e.g. placebo or standard care), with full findings presented in an appendix. League tables and forest plots may be considered to summarize pairwise comparisons.* If additional summary measures were explored (such as treatment rankings), these should also be presented. | 10 |
| **Exploration for inconsistency** | **S5** | Describe results from investigations of inconsistency. This may include such information as measures of model fit to compare consistency and inconsistency models, *P* values from statistical tests, or summary of inconsistency estimates from different parts of the treatment network. | Appendix 3 |
| Risk of bias across studies | 22 | Present results of any assessment of risk of bias across studies for the evidence base being studied. | 10 |
| Results of additional analyses | 23 | Give results of additional analyses, if done (e.g., sensitivity or subgroup analyses, meta-regression analyses*, alternative network geometries studied, alternative choice of prior distributions for Bayesian analyses,* and so forth). | none |
| **DISCUSSION** |  |  |  |
| Summary of evidence | 24 | Summarize the main findings, including the strength of evidence for each main outcome; consider their relevance to key groups (e.g., healthcare providers, users, and policy-makers). | 15 |
| Limitations | 25 | Discuss limitations at study and outcome level (e.g., risk of bias), and at review level (e.g., incomplete retrieval of identified research, reporting bias). *Comment on the validity of the assumptions, such as transitivity and consistency. Comment on any concerns regarding network geometry (e.g., avoidance of certain comparisons).* | 17 |
| Conclusions | 26 | Provide a general interpretation of the results in the context of other evidence, and implications for future research. | 18 |
| **FUNDING** |  |  |  |
| Funding | 27 | Describe sources of funding for the systematic review and other support (e.g., supply of data); role of funders for the systematic review. This should also include information regarding whether funding has been received from manufacturers of treatments in the network and/or whether some of the authors are content experts with professional conflicts of interest that could affect use of treatments in the network. | ***none*** |

**Appendix 2: Search strategy**

**Table S1.** Search strategy of PubMed

| **#** | **Searches** |
| --- | --- |
| 1 | "Breast Neoplasms"[Mesh] |
| 2 | (((((((((((((((((((((((((((((((((((((Breast Neoplasms[Title/Abstract]) OR (Breast Neoplasm[Title/Abstract])) OR (Neoplasm, Breast[Title/Abstract])) OR (Breast Tumors[Title/Abstract])) OR (Breast Tumor[Title/Abstract])) OR (Tumor, Breast[Title/Abstract])) OR (Tumors, Breast[Title/Abstract])) OR (Neoplasms, Breast[Title/Abstract])) OR (Breast Cancer[Title/Abstract])) OR (Cancer, Breast[Title/Abstract])) OR (Mammary Cancer[Title/Abstract])) OR (Cancer, Mammary[Title/Abstract])) OR (Cancers, Mammary[Title/Abstract])) OR (Mammary Cancers[Title/Abstract])) OR (Mammary Cancers[Title/Abstract])) OR (Breast Malignant Neoplasm[Title/Abstract])) OR (Breast Malignant Neoplasms[Title/Abstract])) OR (Malignant Tumor of Breast[Title/Abstract])) OR (Breast Malignant Tumor[Title/Abstract])) OR (Breast Malignant Tumors[Title/Abstract])) OR (Cancer of Breast[Title/Abstract])) OR (Cancer of the Breast[Title/Abstract])) OR (Mammary Carcinoma, Human[Title/Abstract])) OR (Carcinoma, Human Mammary[Title/Abstract])) OR (Carcinomas, Human Mammary[Title/Abstract])) OR (Human Mammary Carcinomas[Title/Abstract])) OR (Mammary Carcinomas, Human[Title/Abstract])) OR (Human Mammary Carcinoma[Title/Abstract])) OR (Mammary Neoplasms, Human[Title/Abstract])) OR (Human Mammary Neoplasm[Title/Abstract])) OR (Human Mammary Neoplasms[Title/Abstract])) OR (Neoplasm, Human Mammary[Title/Abstract])) OR (Neoplasms, Human Mammary[Title/Abstract])) OR (Mammary Neoplasm, Human[Title/Abstract])) OR (Breast Carcinoma[Title/Abstract])) OR (Breast Carcinomas[Title/Abstract])) OR (Carcinoma, Breast[Title/Abstract])) OR (Carcinomas, Breast[Title/Abstract]) |
| 3 | #1 OR #2 |
| 4 | ((((((CDK4/6 inhibitors[Title/Abstract]) OR (CDK4/6 inhibitor*[Title/Abstract])) OR (cyclin-dependent kinase 4/6 inhibitor*[Title/Abstract])) OR (cyclin-dependent kinase 4[Title/Abstract] AND 6 inhibitor*[Title/Abstract])) OR (CDK4[Title/Abstract] AND CDK6 inhibitor*[Title/Abstract])) OR (CDK inhibitor*[Title/Abstract])) OR (CDKi[Title/Abstract]) |
| 5 | (((abemaciclib[Title/Abstract]) OR (palbociclib[Title/Abstract])) OR (dalpiciclib[Title/Abstract])) OR (ribociclib[Title/Abstract]))OR (bireociclib[Title/Abstract])) OR (lerociclib[Title/Abstract]))OR (tibremciclib [Title/Abstract]) |
| 6 | #4 OR #5 |
| 7 | "Fulvestrant"[Mesh] |
| 8 | (Fulvestrant[Title/Abstract]) OR (Faslodex[Title/Abstract]) |
| 9 | #7 OR #8 |
| 10 | "Randomized Controlled Trial" [Publication Type] |
| 11 | (((((((((Randomized Controlled Trial[Title/Abstract]) OR (controlled clinical trial[Title/Abstract])) OR (randomized controlled trials[Title/Abstract])) OR (random allocation[Title/Abstract])) OR (clinical trial[Title/Abstract])) OR (placebo[Title/Abstract])) OR (placebo*[Title/Abstract])) OR (random*[Title/Abstract])) OR (double-blind method[Title/Abstract])) OR (clinical trials[Title/Abstract]) |
| 12 | #10 OR #11 |
| 13 | #3 AND #6 AND #9 AND #12 |

**Table S2.** Search strategy of Web of Science

| **#** | **Searches** |
| --- | --- |
| 1 | Breast Neoplasms (Topic) OR Breast Neoplasm (Topic) OR Neoplasm, Breast (Topic) OR Breast Tumors (Topic) OR Breast Tumor (Topic) OR Tumor, Breast (Topic) OR Tumors, Breast (Topic) OR Neoplasms, Breast (Topic) OR Breast Cancer (Topic) OR Cancer, Breast (Topic) OR Mammary Cancer (Topic) OR Cancer, Mammary (Topic) OR Cancers, Mammary (Topic) OR Mammary Cancers (Topic) OR Malignant Neoplasm of Breast (Topic) OR Breast Malignant Neoplasm (Topic) OR Breast Malignant Neoplasms (Topic) OR Malignant Tumor of Breast (Topic) OR Breast Malignant Tumor (Topic) OR Breast Malignant Tumors (Topic) OR Cancer of Breast (Topic) OR Cancer of the Breast (Topic) OR Mammary Carcinoma, Human (Topic) OR Carcinoma, Human Mammary (Topic) OR Carcinomas, Human Mammary (Topic) OR Human Mammary Carcinomas (Topic) OR Mammary Carcinomas, Human (Topic) OR Human Mammary Carcinoma (Topic) OR Mammary Neoplasms, Human (Topic) OR Human Mammary Neoplasm (Topic) OR Human Mammary Neoplasms (Topic) OR Neoplasm, Human Mammary (Topic) OR Neoplasms, Human Mammary (Topic) OR Mammary Neoplasm, Human (Topic) OR Breast Carcinoma (Topic) OR Breast Carcinomas (Topic) OR Carcinoma, Breast (Topic) OR Carcinomas, Breast (Topic) and Preprint Citation Index (Exclude – Database) |
| 2 | CDK4/6 inhibitors (Topic) OR CDK4/6 inhibitor* (Topic) OR cyclin-dependent kinase 4/6 inhibitor* (Topic) OR cyclin-dependent kinase 4 and 6 inhibitor* (Topic) OR CDK4 and CDK6 inhibitor* (Topic) OR CDK inhibitor* (Topic) OR CDKi (Topic) OR abemaciclib (Topic) OR palbociclib (Topic) OR dalpiciclib (Topic) OR ribociclib (Topic) OR bireociclib (Topic) OR lerociclib (Topic) OR tibremciclib (Topic)and Preprint Citation Index (Exclude – Database) |
| 3 | Fulvestrant (Topic) OR Faslodex (Topic) and Preprint Citation Index (Exclude – Database) |
| 4 | Randomized Controlled Trial (Topic) OR controlled clinical trial (Topic) OR randomized controlled trials (Topic) OR clinical trial (Topic) OR placebo (Topic) OR placebo* (Topic) OR random* (Topic) OR clinical trials (Topic) OR random allocation (Topic) OR double-blind method (Topic) and Preprint Citation Index (Exclude – Database) |
| 5 | #1 AND #2 AND #3 AND #4 and Preprint Citation Index (Exclude – Database) |

**Table S3.** Search strategy of Cochrane Central Register of Controlled Trials

| **#** | **Searches** |
| --- | --- |
| 1 | MeSH descriptor: [Breast Neoplasms] explode all trees |
| 2 | (Breast Neoplasms):ti,ab,kw OR (Breast Neoplasm):ti,ab,kw OR (Neoplasm, Breast):ti,ab,kw OR (Breast Tumors):ti,ab,kw OR (Breast Tumor):ti,ab,kw OR (Tumor, Breast):ti,ab,kw OR (Tumors, Breast):ti,ab,kw OR (Neoplasms, Breast):ti,ab,kw OR (Breast Cancer):ti,ab,kw OR (Cancer, Breast):ti,ab,kw OR (Mammary Cancer):ti,ab,kw OR (Cancer, Mammary):ti,ab,kw OR (Cancers, Mammary):ti,ab,kw OR (Mammary Cancers):ti,ab,kw OR (Malignant Neoplasm of Breast):ti,ab,kw OR (Breast Malignant Neoplasm):ti,ab,kw OR (Breast Malignant Neoplasms):ti,ab,kw OR (Malignant Tumor of Breast):ti,ab,kw OR (Breast Malignant Tumor):ti,ab,kw OR (Breast Malignant Tumors):ti,ab,kw OR (Cancer of Breast):ti,ab,kw OR (Cancer of the Breast):ti,ab,kw OR (Mammary Carcinoma, Human):ti,ab,kw OR (Carcinoma, Human Mammary):ti,ab,kw OR (Carcinomas, Human Mammary):ti,ab,kw OR (Human Mammary Carcinomas):ti,ab,kw OR (Mammary Carcinomas, Human):ti,ab,kw OR (Human Mammary Carcinoma):ti,ab,kw OR (Mammary Neoplasms, Human):ti,ab,kw OR (Human Mammary Neoplasm):ti,ab,kw OR (Human Mammary Neoplasms):ti,ab,kw OR (Neoplasm, Human Mammary):ti,ab,kw OR (Neoplasms, Human Mammary):ti,ab,kw OR (Mammary Neoplasm, Human):ti,ab,kw OR (Breast Carcinoma):ti,ab,kw OR (Breast Carcinomas):ti,ab,kw OR (Carcinoma, Breast):ti,ab,kw OR (Carcinomas, Breast):ti,ab,kw OR |
| 3 | #1 or #2 |
| 4 | (CDK4/6 inhibitors):ti,ab,kw OR (CDk4/6 inhibitor*):ti.ab,kw OR (cyclin-dependent kinase 4/6 inhibitor*):ti,ab,kw OR (cycin-dependent kinase 4 and 6 inhibitor*):ti,ab,kw OR (CDK4 and CDK6 inhibitor*):ti, ab,kw OR (CDK inhibitor*):ti,ab, kw OR (CDKi):ti,ab,kw |
| 5 | (abemacicib);ti,ab,kw OR (palbociclib);ti,ab,kw OR (dalpiciclib);ti,ab,kw OR (ribociclib);ti,ab,kw OR (bireociclib);ti,ab,kw OR (lerociclib);ti,ab,kwOR (tibremciclib);ti,ab,kw |
| 6 | #4 or #5 |
| 7 | MeSH descriptor: [Fulvestrant] explode all trees |
| 8 | (Fulvestrant):ti,ab,kw OR (Faslodex):ti,ab,kw |
| 9 | #7 or #8 |
| 10 | MeSH descriptor: [Randomized Controlled Trial] explode all trees |
| 11 | (Randomized Controled Trial):ti.ab,kw OR (controled clinical trial;ti,ab,.kw OR (randomized controled tials):t;ab,kw OR (random alocation.:i,ab,kw oR (clinicaltrial):ti,ab,kw oR (placebo):ti,ab,kaw oR (placebo* ):ti,ab,kw oR (random*):ti,ab,kw oR (double-blind method):t,ab,kw OR (cinical trials);ti,ab,kw |
| 12 | #10 or #11 |
| 13 | #3 AND #6 AND #9 AND #12 |

**Table S4.** Search strategy of Embase

| **#** | **Searches** |
| --- | --- |
| 1 | 'bilateral breast neoplasm':ab,ti OR 'bilateral breast tumor':ab,ti OR 'bilateral breast tumour':ab,ti OR 'breast gland tumor':ab,ti OR 'breast gland tumour':ab,ti OR 'breast mass':ab,ti OR 'breast neoplasia':ab,ti OR 'breast neoplasm':ab,ti OR 'breast neoplasms':ab,ti OR 'breast neoplasms, male':ab,ti OR 'breast tumorigenesis':ab,ti OR 'breast tumour':ab,ti OR 'female breast neoplasm':ab,ti OR 'female breast tumor':ab,ti OR 'female breast tumour':ab,ti OR 'male breast neoplasm':ab,ti OR 'male breast tumor':ab,ti OR 'male breast tumour':ab,ti OR 'mamma tumor':ab,ti OR 'mamma tumour':ab,ti OR 'mammary gland neoplasia':ab,ti OR 'mammary gland neoplasm':ab,ti OR 'mammary gland tumor':ab,ti OR 'mammary gland tumorigenesis':ab,ti OR 'mammary gland tumour':ab,ti OR 'mammary neoplasia':ab,ti OR 'mammary neoplasm':ab,ti OR 'mammary neoplasms':ab,ti OR 'mammary tumor':ab,ti OR 'mammary tumor cell':ab,ti OR 'mammary tumorigenesis':ab,ti OR 'mammary tumour':ab,ti OR 'mammary tumour cell':ab,ti OR 'mass in the breast':ab,ti OR 'masses in the breast':ab,ti OR 'neoplasia of the breast':ab,ti OR 'neoplasm of the breast':ab,ti OR 'neoplasm of the mammary gland':ab,ti OR 'neoplastic breast':ab,ti OR 'neoplastic mammary':ab,ti OR 'neoplastic mammary gland':ab,ti OR 'tumor of the breast':ab,ti OR 'tumor of the female breast':ab,ti OR 'tumor of the male breast':ab,ti OR 'tumor of the mammary gland':ab,ti OR 'tumorigenesis of the breast':ab,ti OR 'tumorigenesis of the mammary gland':ab,ti OR 'tumour of the male breast':ab,ti OR 'unilateral breast neoplasm':ab,ti OR 'unilateral breast neoplasms':ab,ti OR 'unilateral breast tumor':ab,ti OR 'breast tumor':ab,ti |
| 2 | breast tumor'/exp |
| 3 | #1 OR #2 |
| 4 | 'cdk4/6 inhibitors':ab,ti OR 'cdk4/6 inhibitor*':ab,ti OR 'cyclin-dependent kinase 4/6 inhibitor*':ab,ti OR ('cyclin-dependent kinase 4':ab,ti AND '6 inhibitor*':ab,ti) OR (cdk4:ab,ti AND 'cdk6 inhibitor*':ab,ti) OR 'cdk inhibitor*':ab,ti OR cdki:ab,ti |
| 5 | abemaciclib:ab,ti OR palbociclib:ab,ti OR dalpiciclib:ab,ti OR ribociclib:ab,ti OR bireociclib:ab,ti OR lerociclib:ab,ti OR tibremciclib:ab,ti |
| 6 | #4 OR #5 |
| 7 | 'fulvestrant'/exp |
| 8 | fulvestrant:ab,ti OR faslodex:ab,ti |
| 9 | #7 OR #8 |
| 10 | 'randomized controlled trial'/exp |
| 11 | 'controlled trial, randomized':ab,ti OR 'randomized controlled trial':ab,ti OR 'randomised controlled study':ab,ti OR 'randomised controlled trial':ab,ti OR 'randomized controlled study':ab,ti OR 'trial, randomized controlled':ab,ti |
| 12 | #10 OR #11 |
| 13 | #3 AND #6 AND #9 AND #12 |

**Appendix 3: Evaluation of consistency and heterogeneity**

| **Clinical Outcome** | **Consistency** | | | **Heterogeneity** |
| --- | --- | --- | --- | --- |
|  | **Dbar** | **pD** | **DIC** | **τ²** |
| **PFS** | 14.520188 | 7.003433 | 21.523621 | 0.175226 |
| **OS** | 2.999338 | 2.99933 | 5.998668 | 0.02010724 |
| **ORR** | 20.16792 | 19.45298 | 39.62090 | 0.3019502 |
| **CBR** | 20.02586 | 19.35406 | 39.37992 | 0.05130678 |
| **QoL** | 8.043101 | 8.042625 | 16.085726 | 6.416596 |
| **AEs** | 14.26284 | 13.48816 | 27.75100 | 0.009639312 |
| **Grade3-4 AEs** | 17.50925 | 17.07346 | 34.58271 | 0.1160765 |
| **SAEs** | 19.17052 | 19.14170 | 38.31222 | 0.1478403 |
| **Treatment discontinuation** | 16.1885 | 16.1322 | 32.3207 | 0.4933658 |

**Note:** PFS, Progression-Free Survival; OS, Overall Survival; ORR, Objective Response Rate; CBR, Clinical Benefit Rate; QoL, Quality of Life; AEs, Adverse Events; Grade 3–4 AEs, Grade 3–4 Adverse Events; SAEs, Serious Adverse Events;

**Appendix 4: Density plots and Trajectory plots of comparisons of each outcome**

**Figure S4.1:** Density plots and Trajectory plots of **PFS**

**
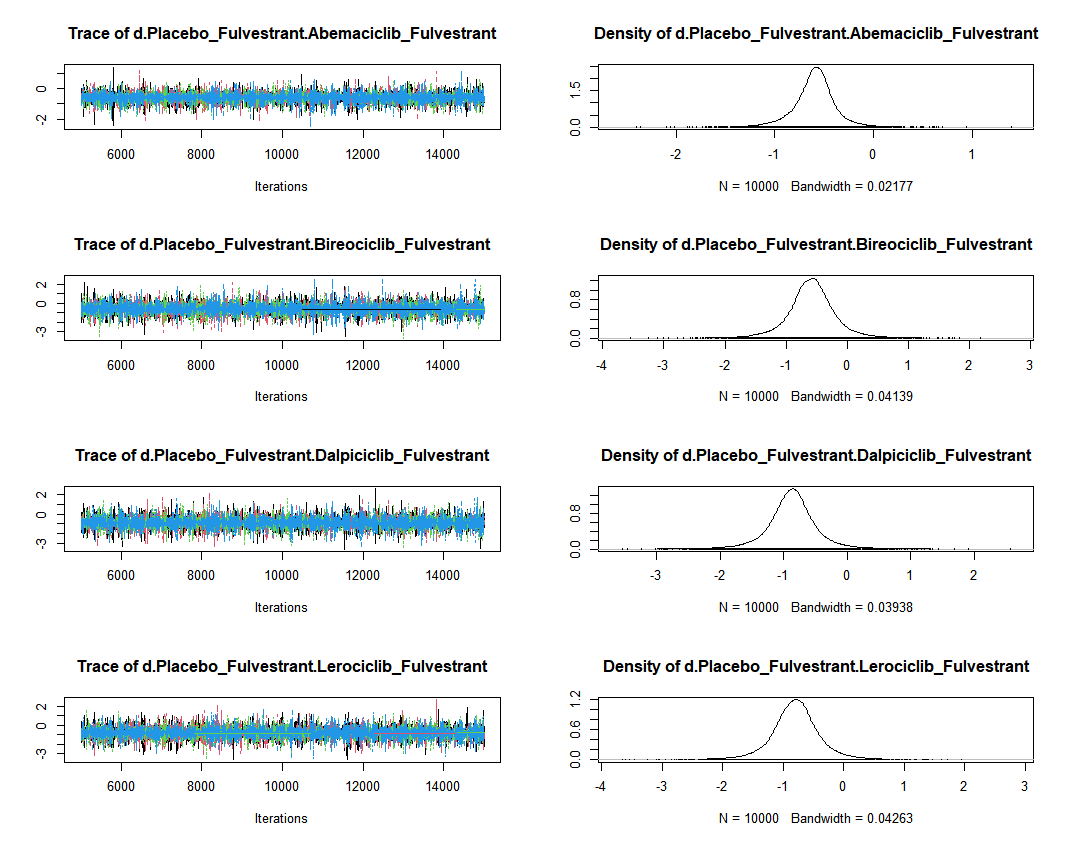
**

**
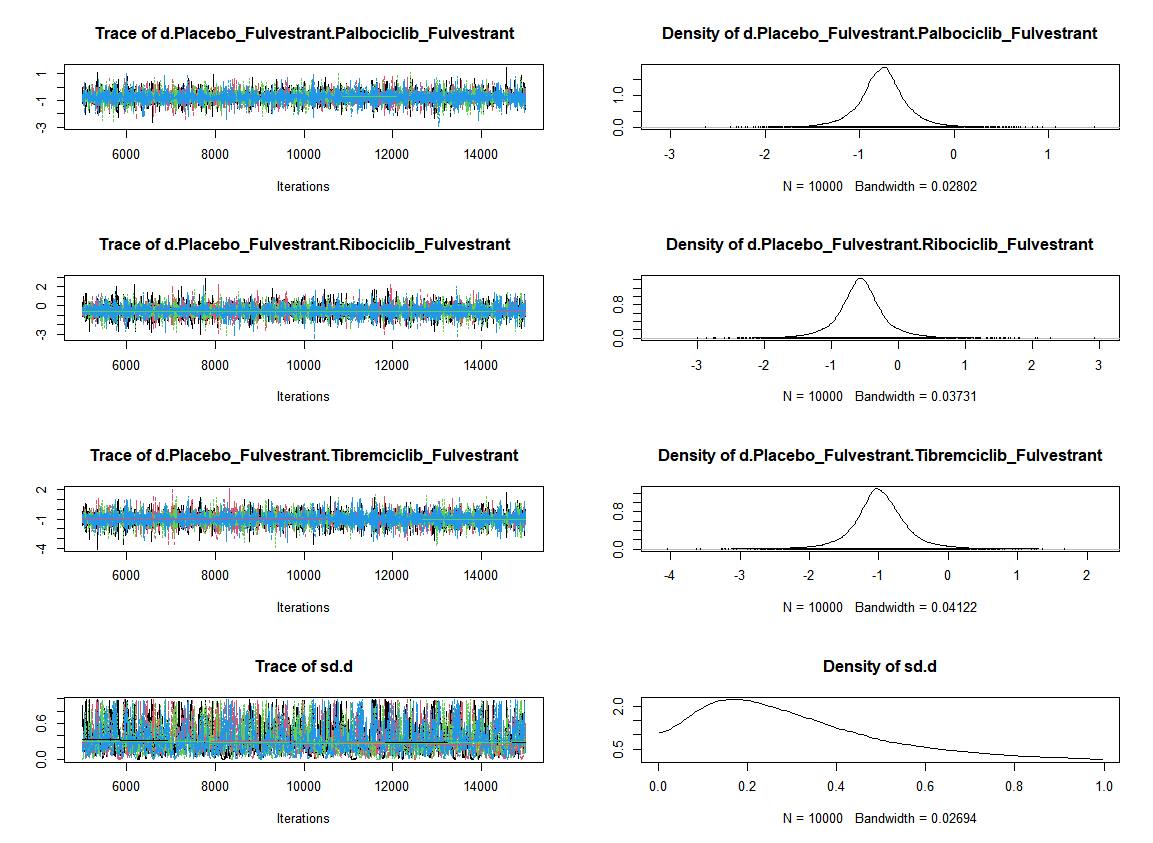
**

**Figure S4.2:** Density plots and Trajectory plots of **OS**

**
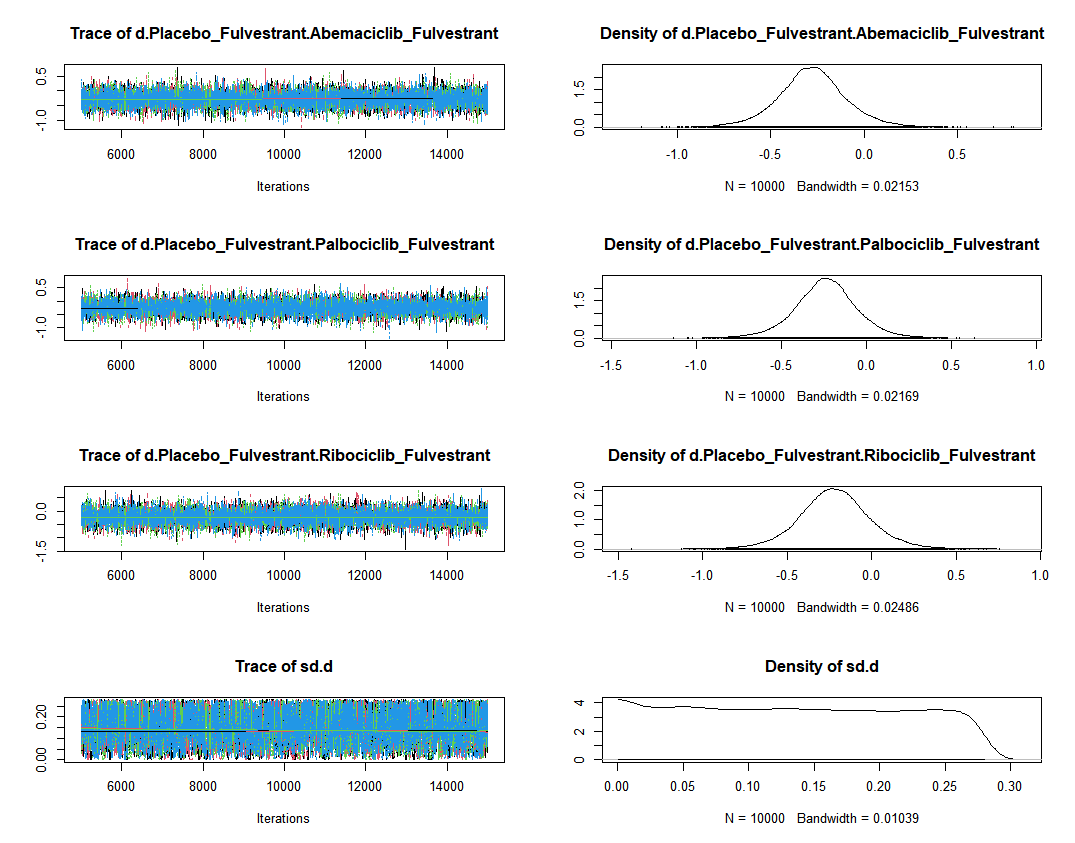
**

**Figure S4.3:** Density plots and Trajectory plots of **ORR**

**
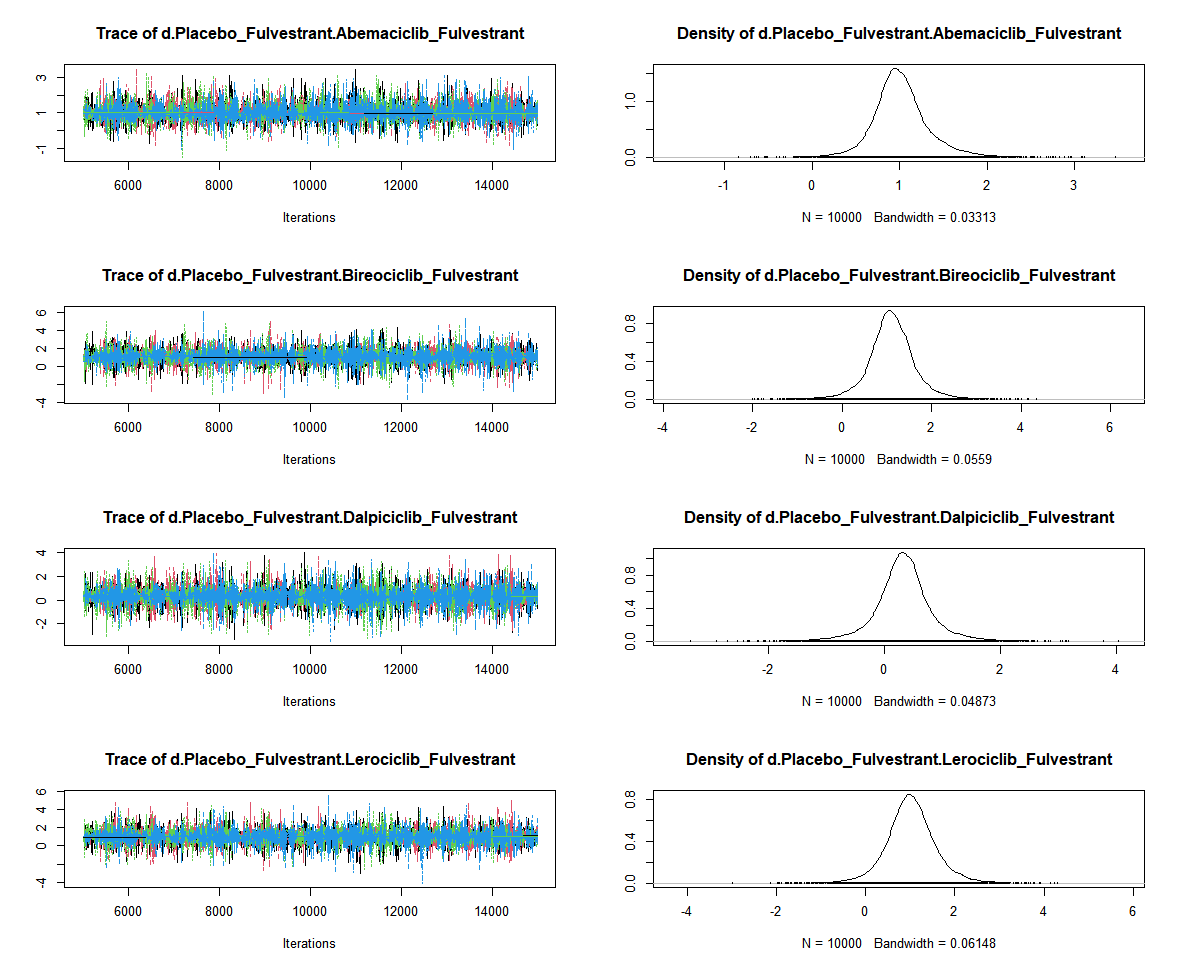
**

**
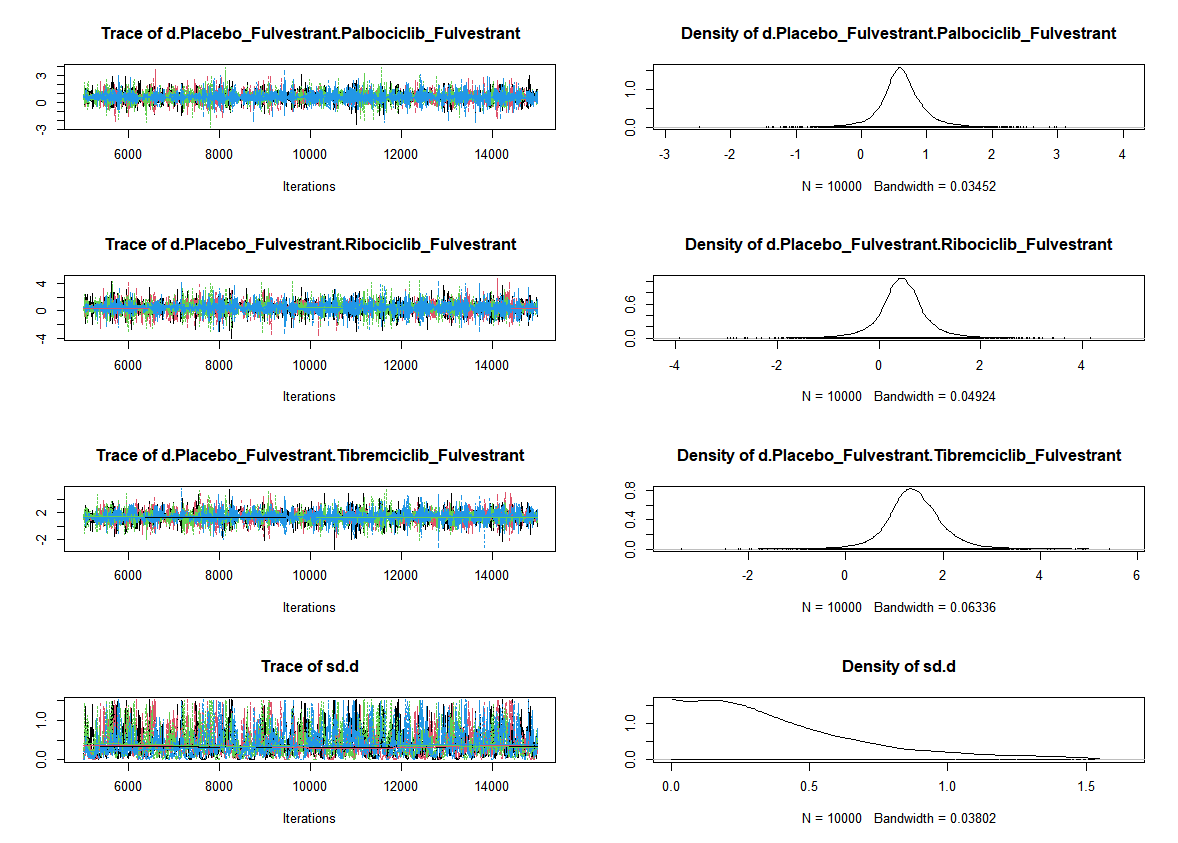
**

**Figure S4.4:** Density plots and Trajectory plots of **CBR**

**
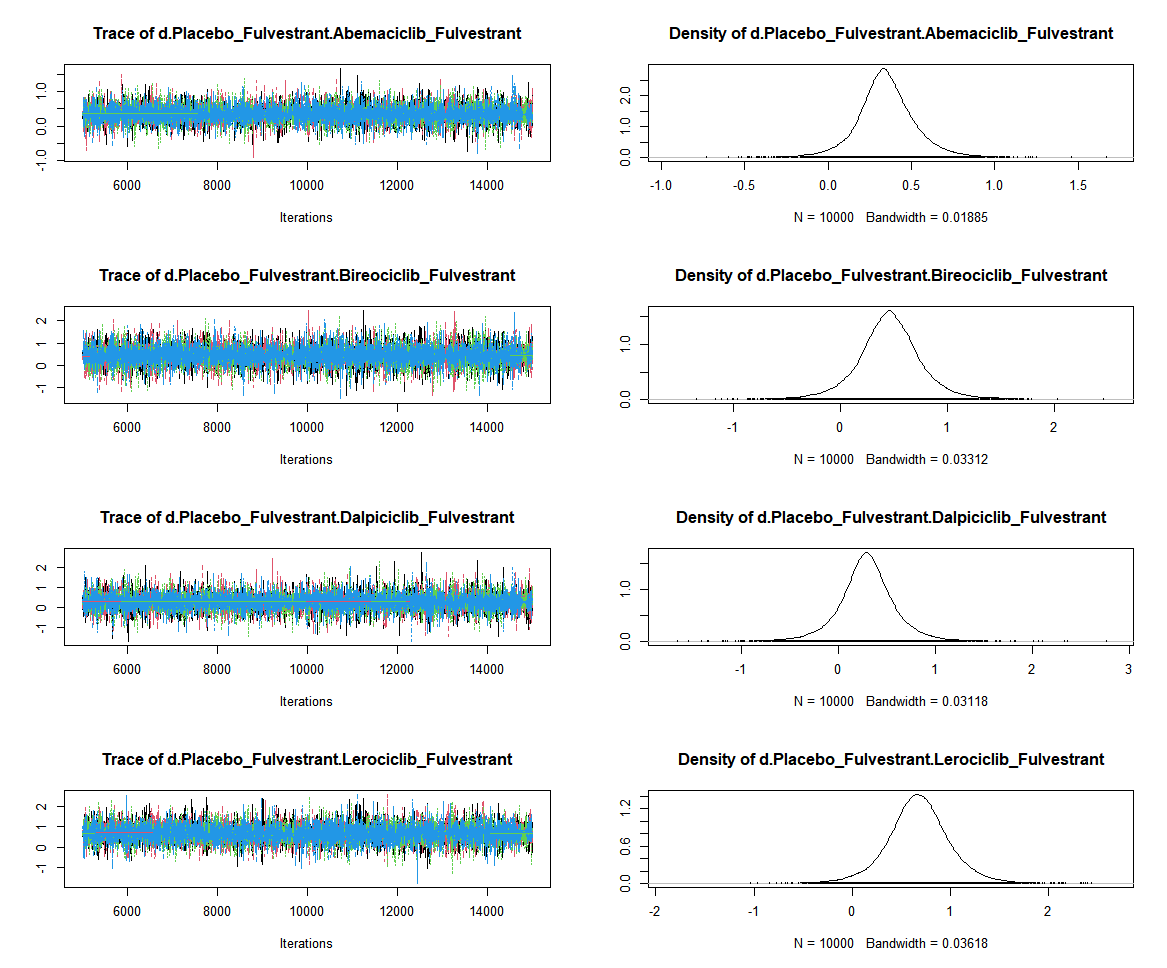
**

**
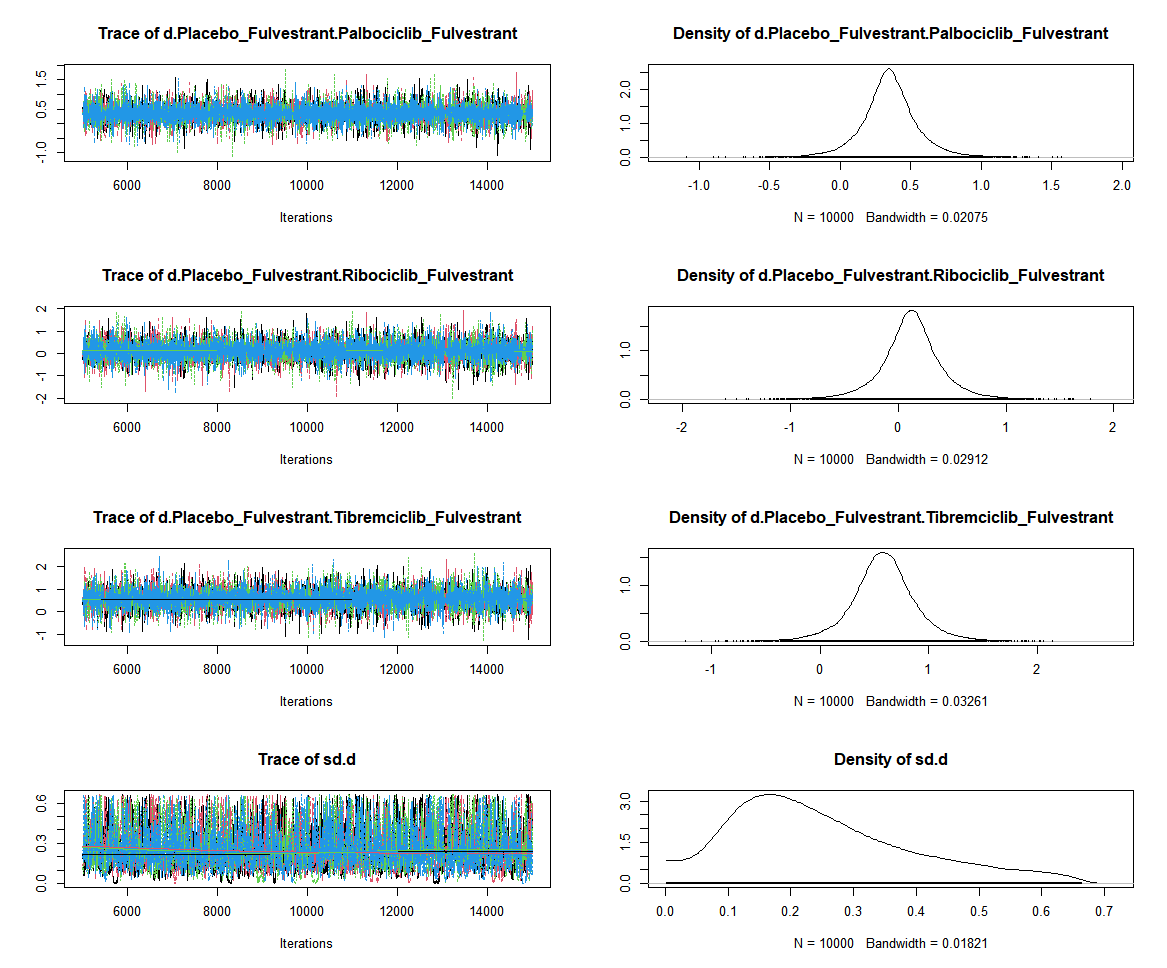
**

**Figure S4.5:** Density plots and Trajectory plots of **QoL**

**
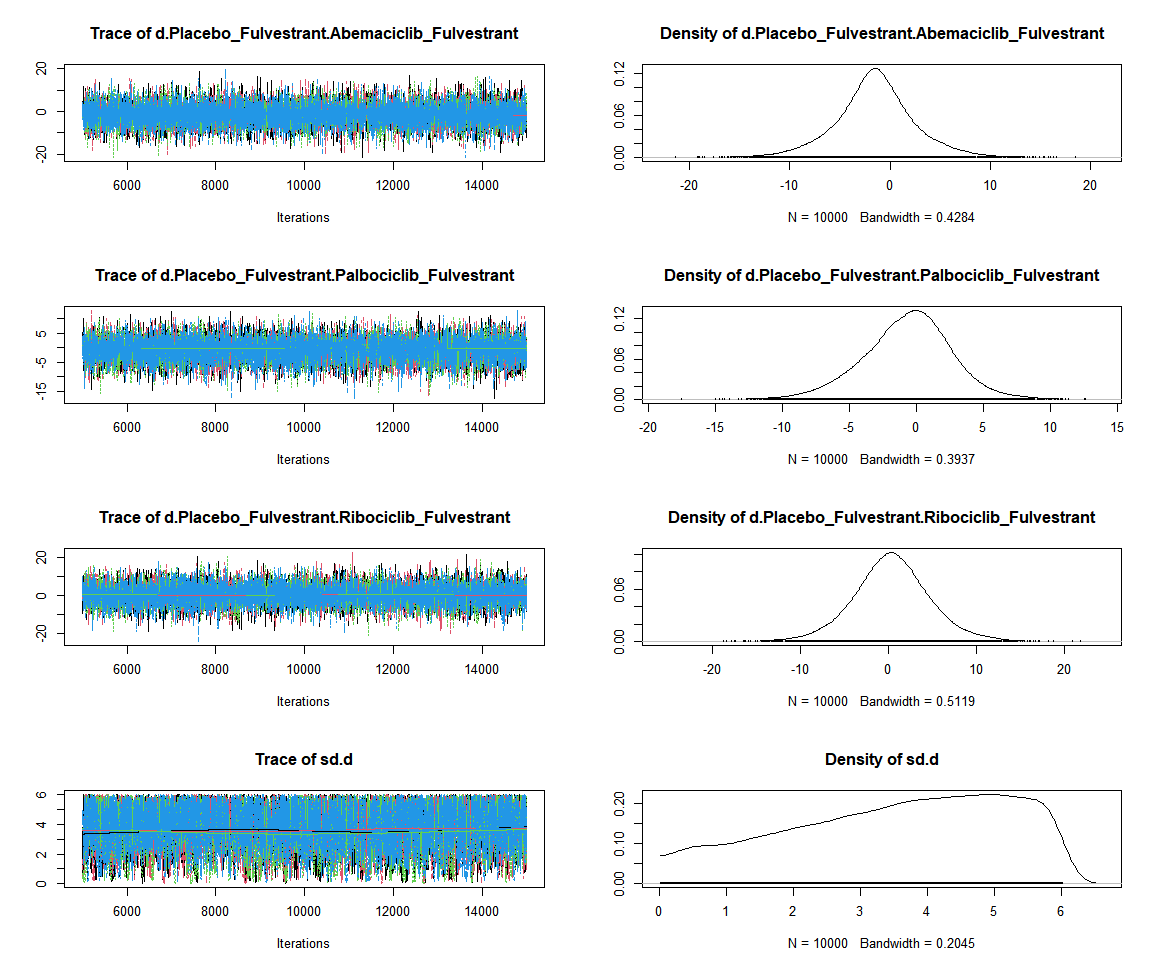
**

**Figure S4.6:** Density plots and Trajectory plots of **AEs**

**
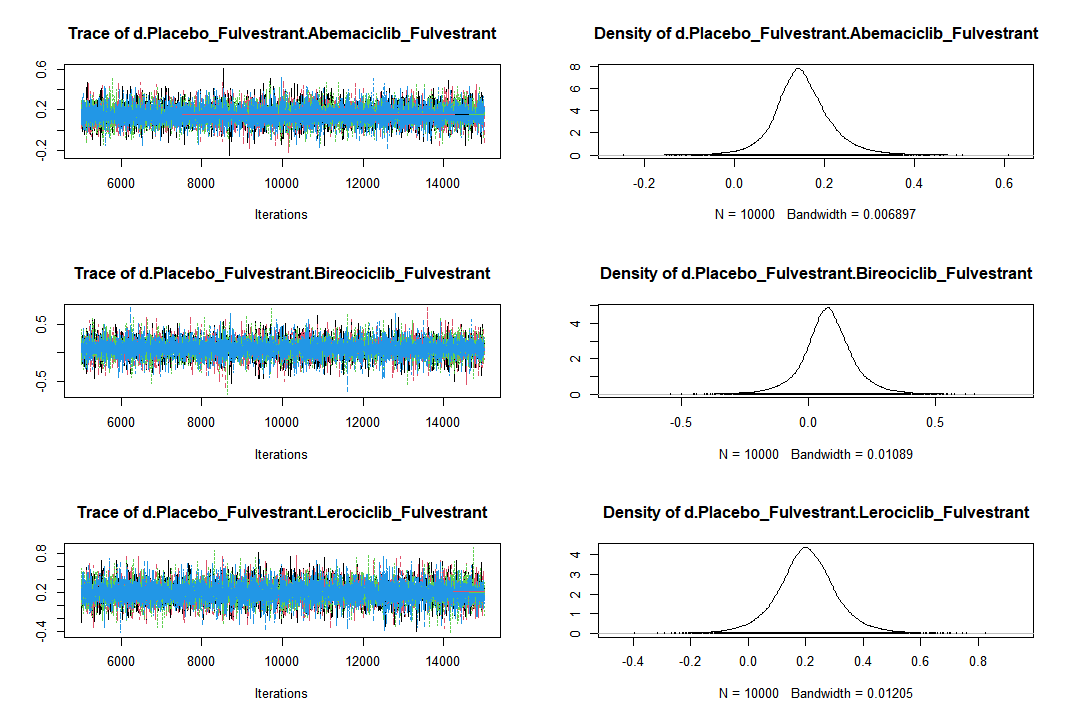
**

**
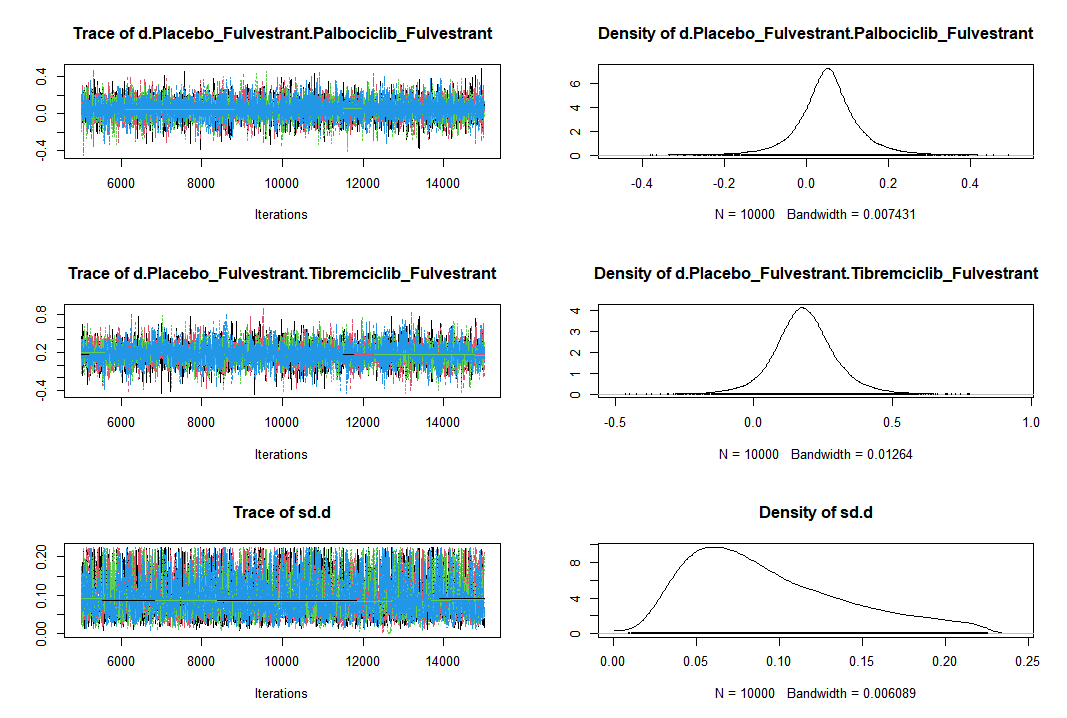
**

**Figure S4.7:** Density plots and Trajectory plots of **Grade 3–4 AEs**

**
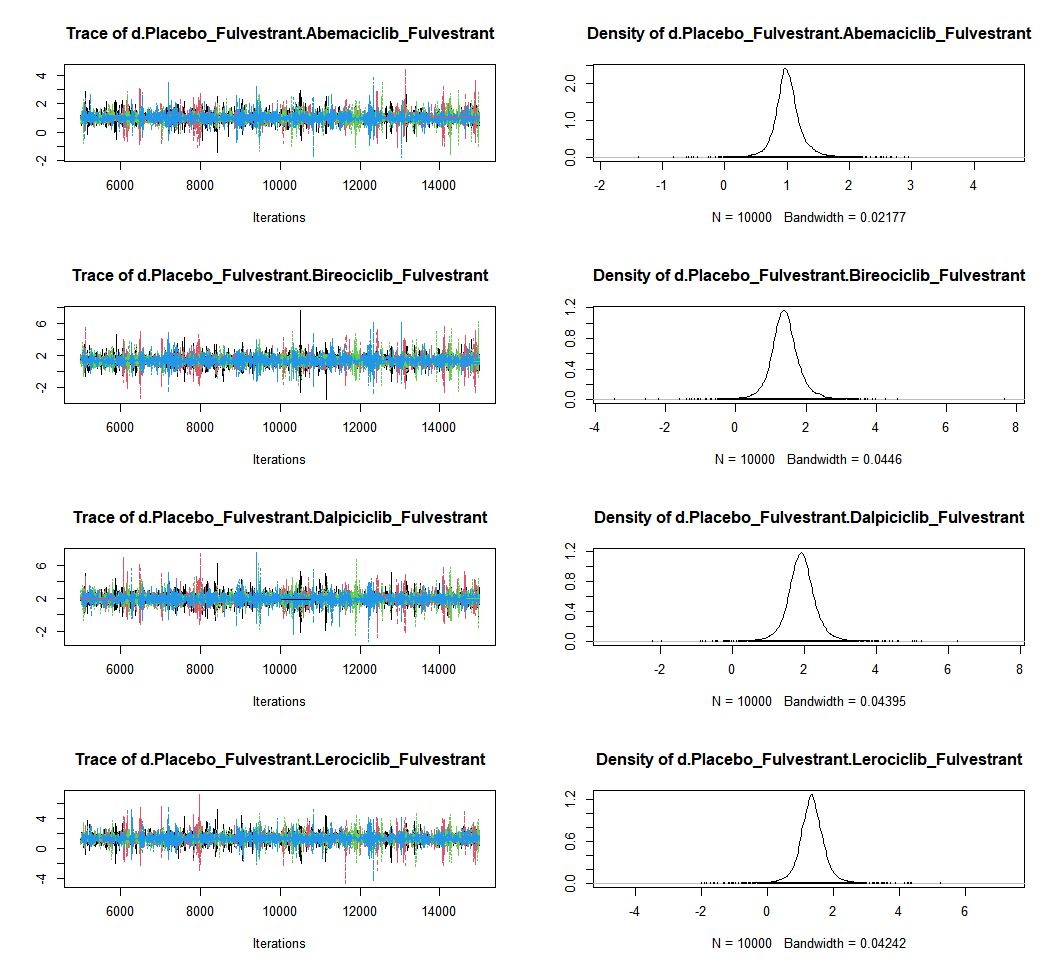
**

**
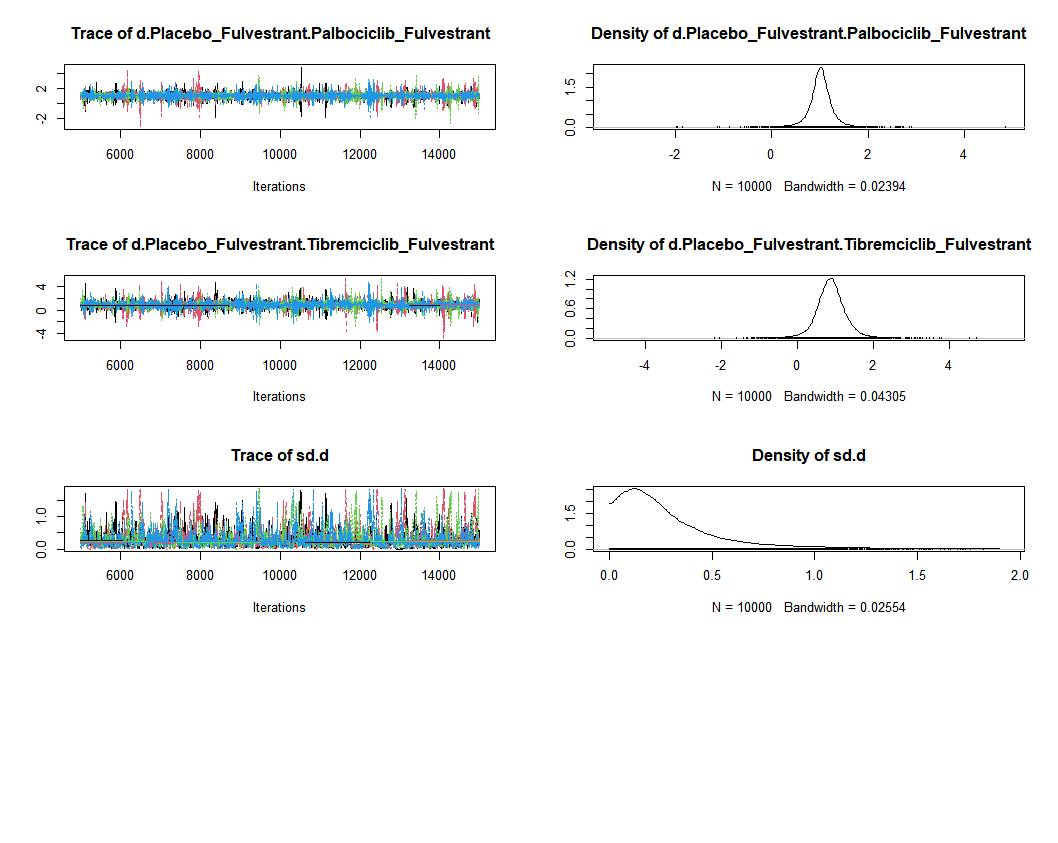
**

**Figure S4.8:** Density plots and Trajectory plots of **SAEs**

**
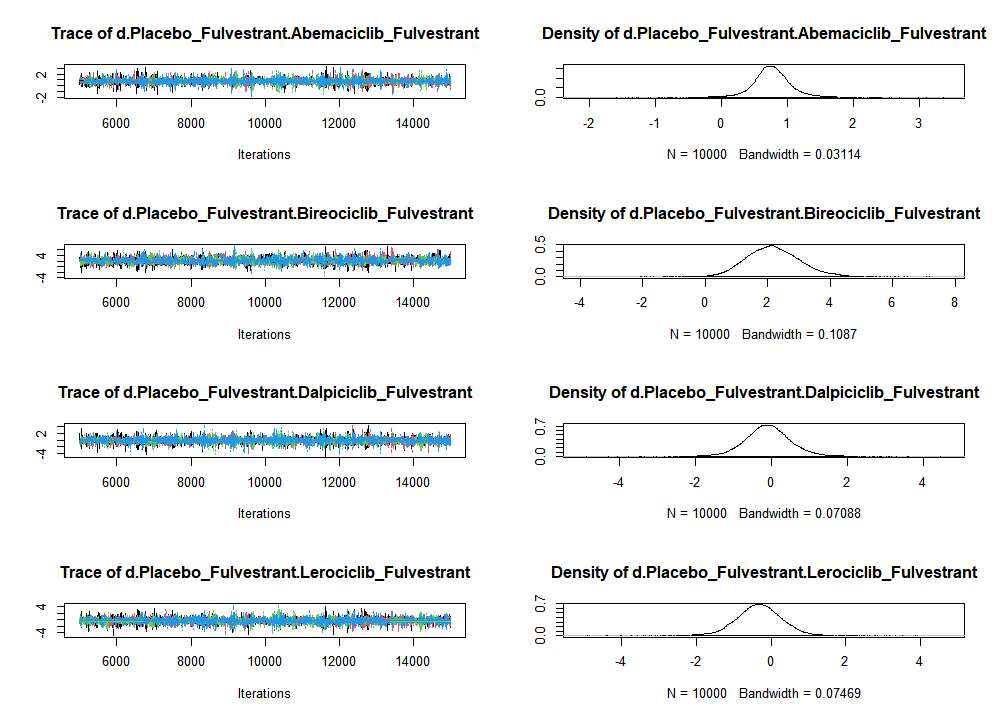
**

**
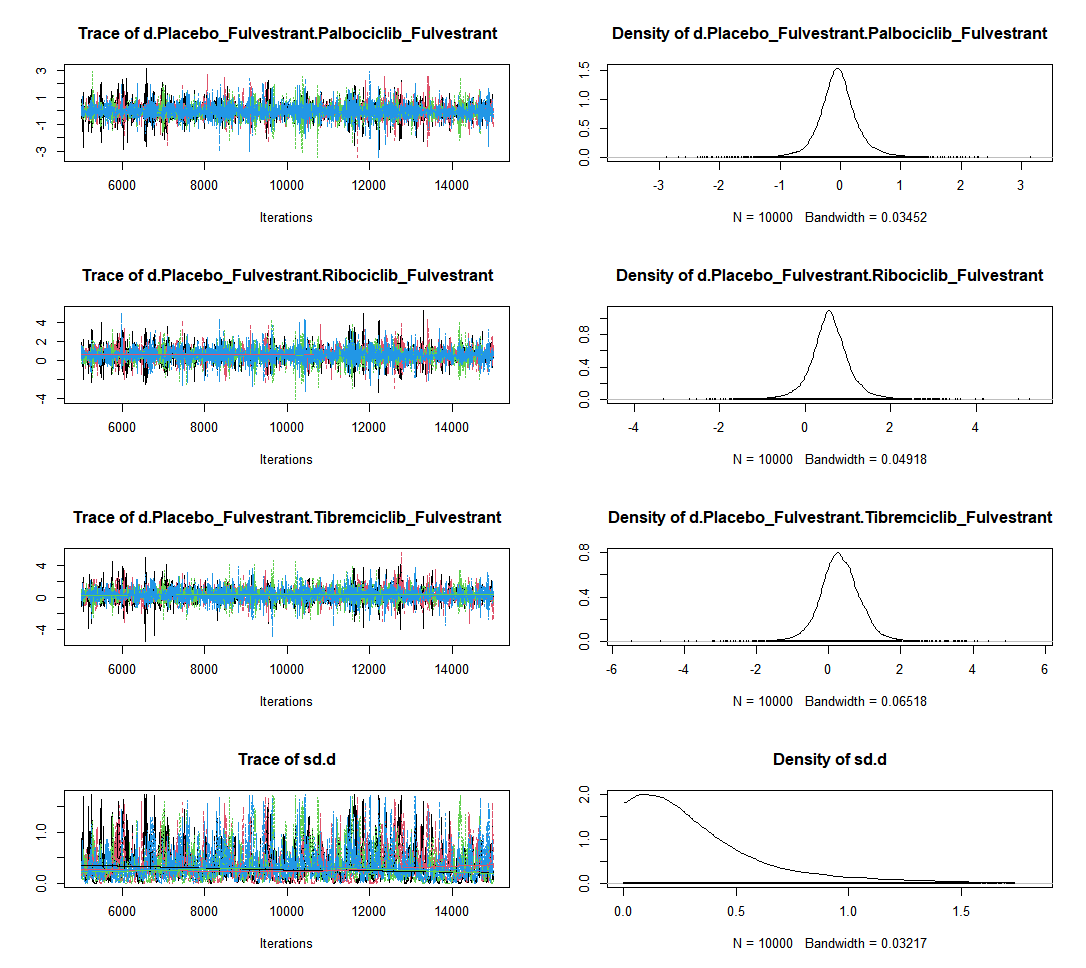
**

**Figure S4.9:** Density plots and Trajectory plots of **Treatment discontinuation due to AEs**

**
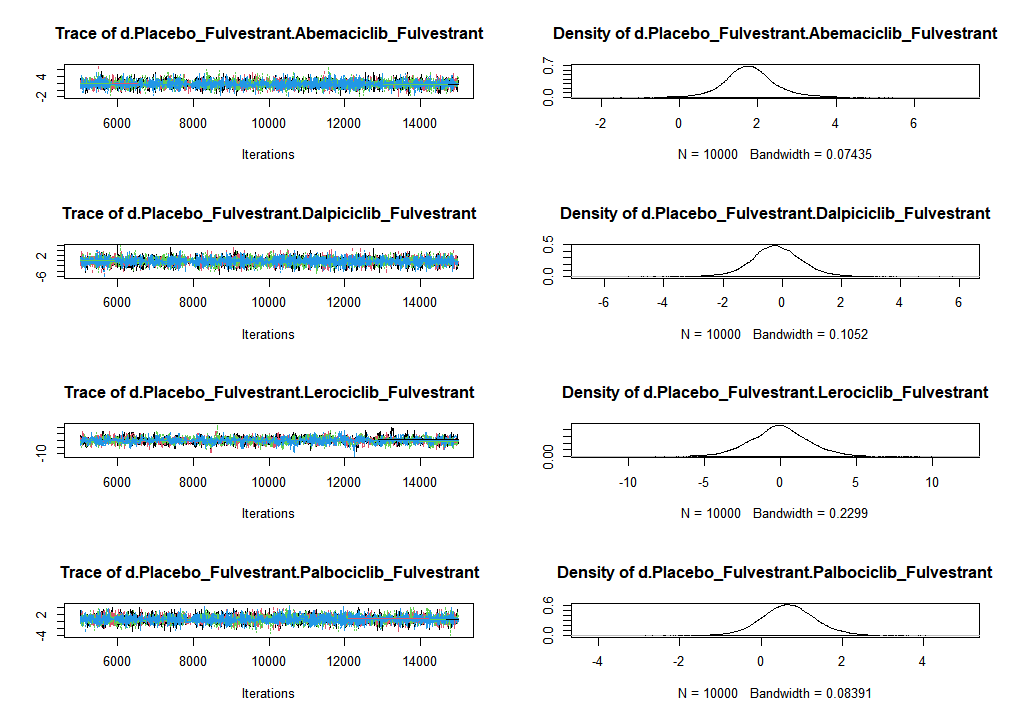
**

**
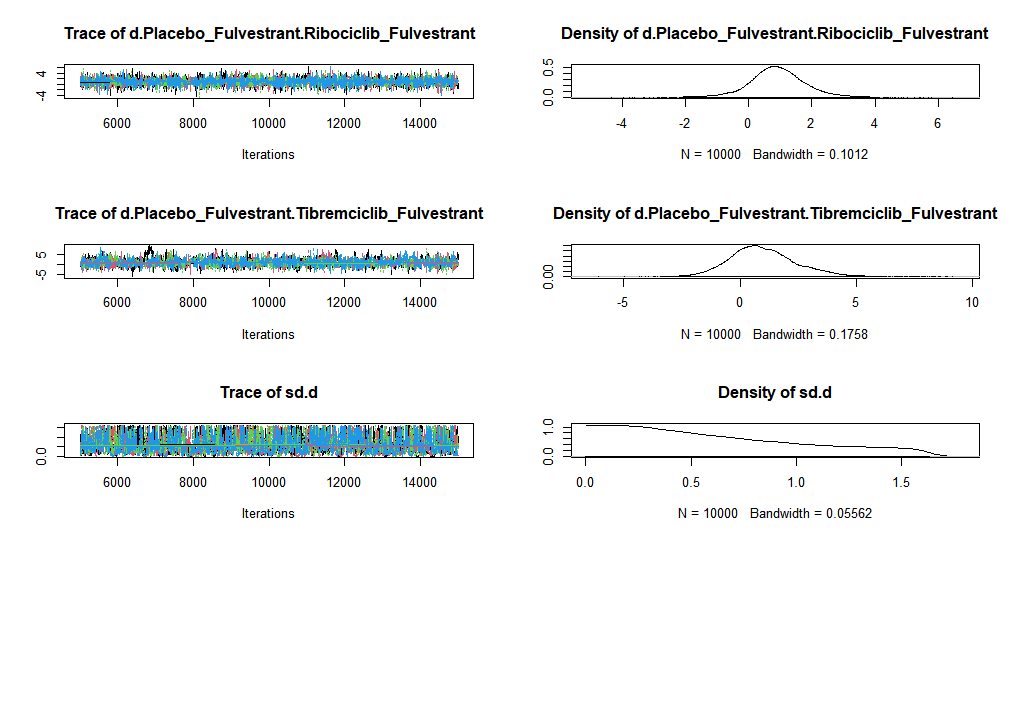
**

**Appendix 5: Convergence Diagnostic Plot of comparisons of each outcome**

**Figure S5.1:** Convergence Diagnostic Plot of **PFS**

**
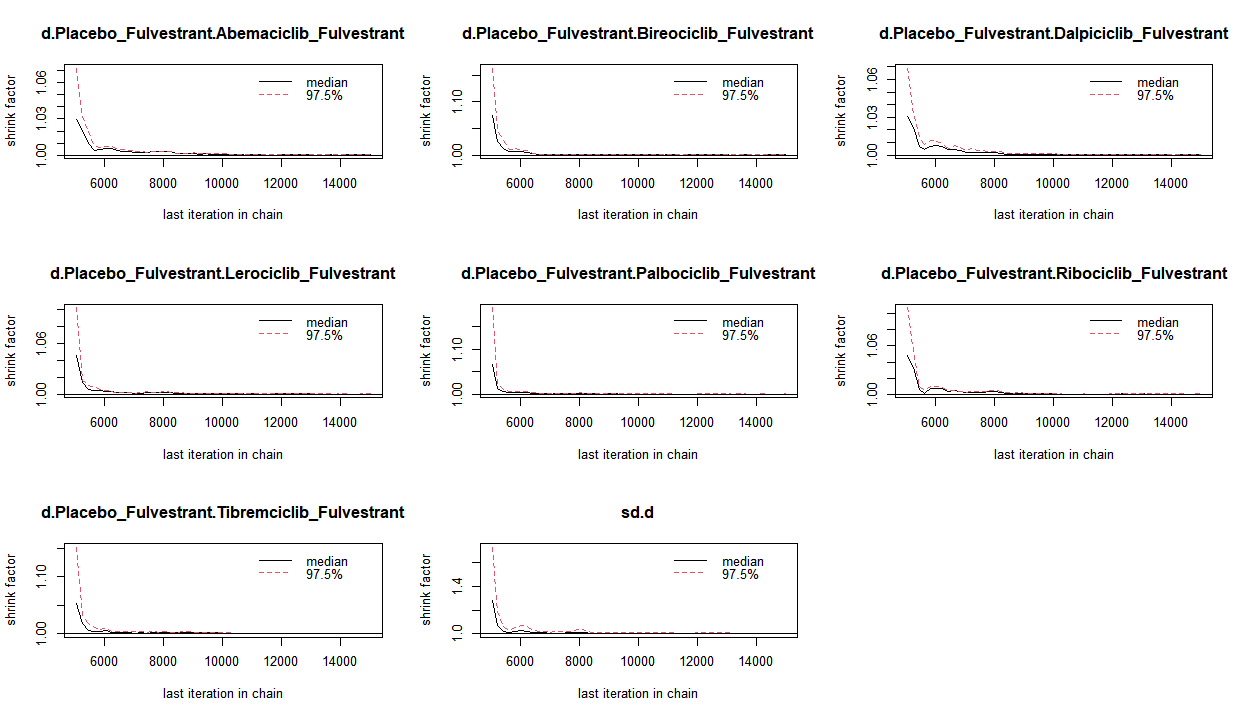
**

**Figure S5.2:** Convergence Diagnostic Plot of **OS**

**
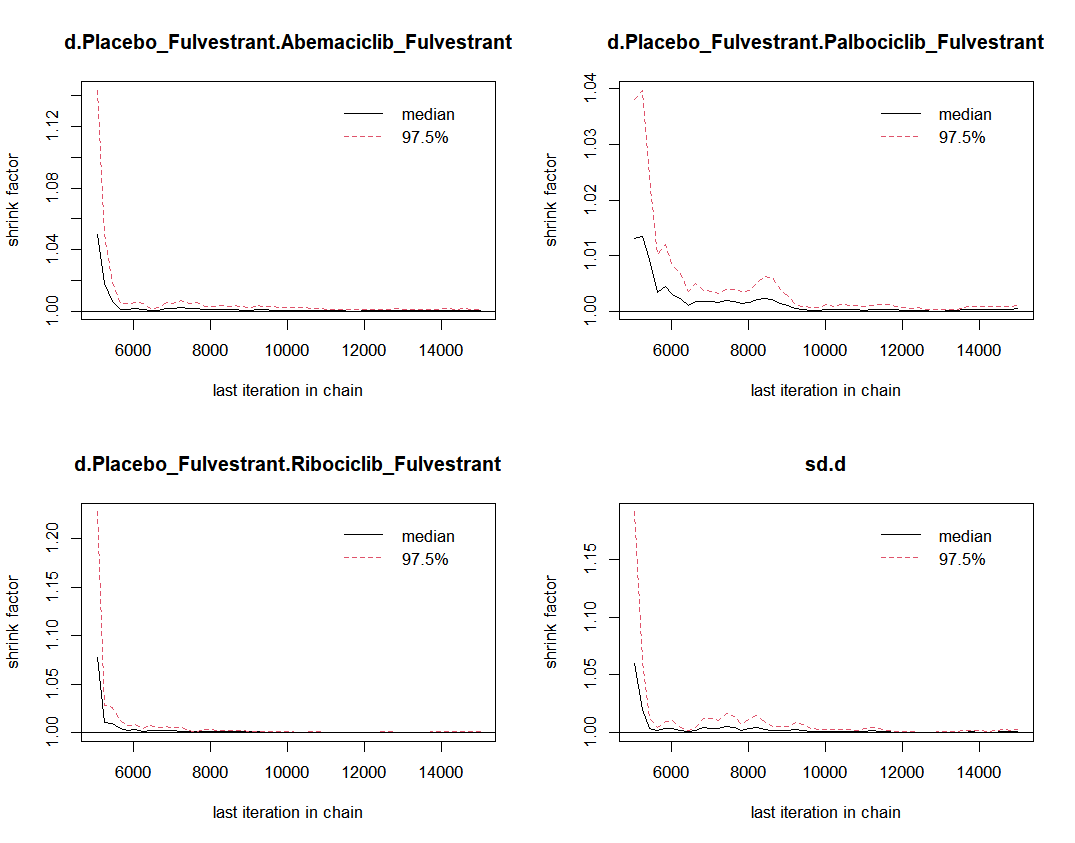
Figure S5.3:** Convergence Diagnostic Plot of **ORR**

**
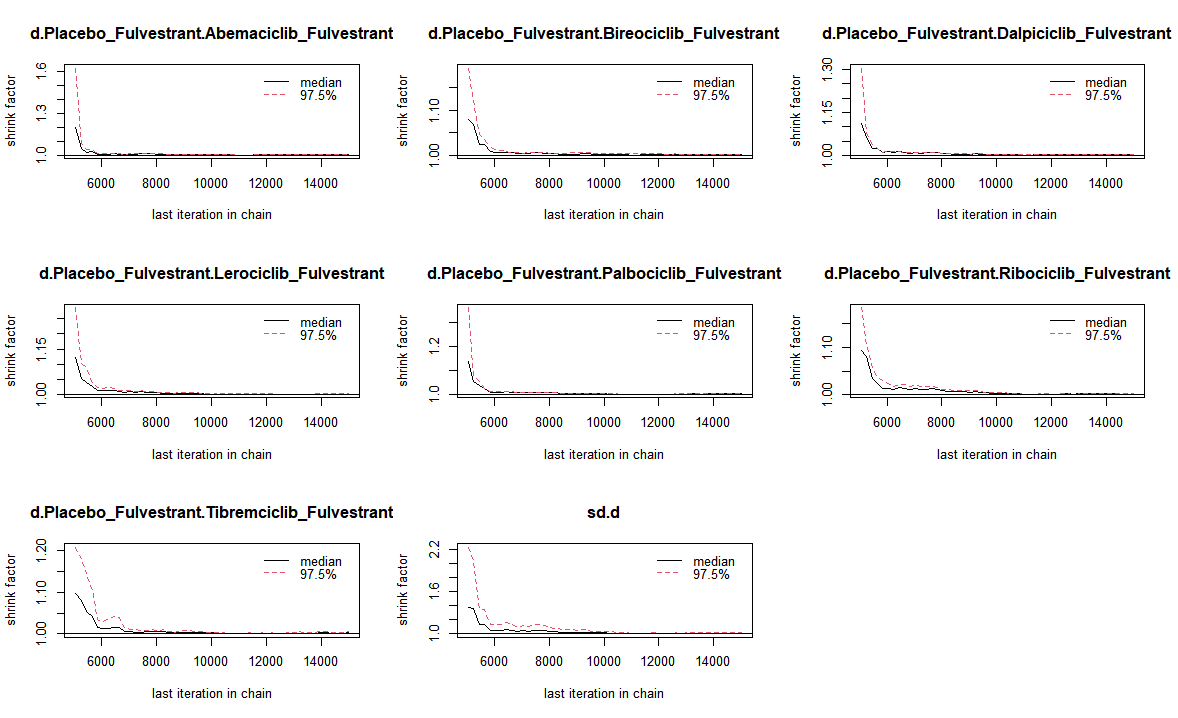
Figure S5.4:** Convergence Diagnostic Plot of **CBR**

**
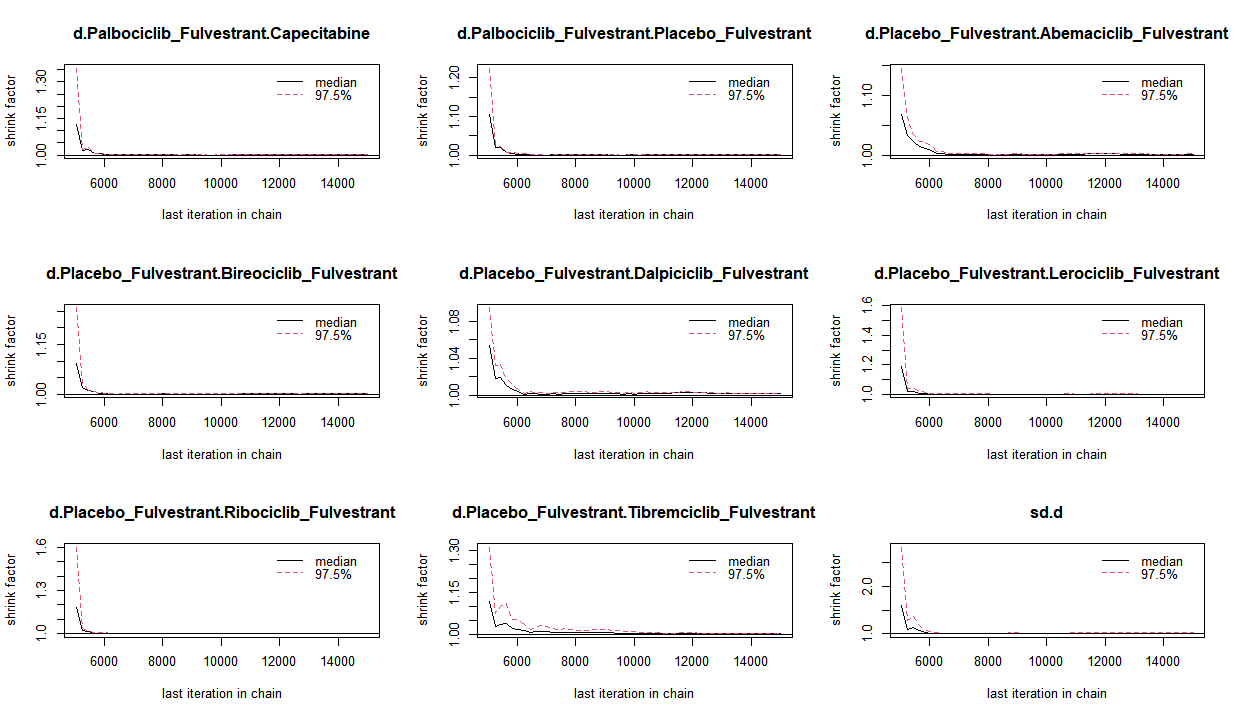
**

**Figure S5.5:** Convergence Diagnostic Plot of **QoL**

**
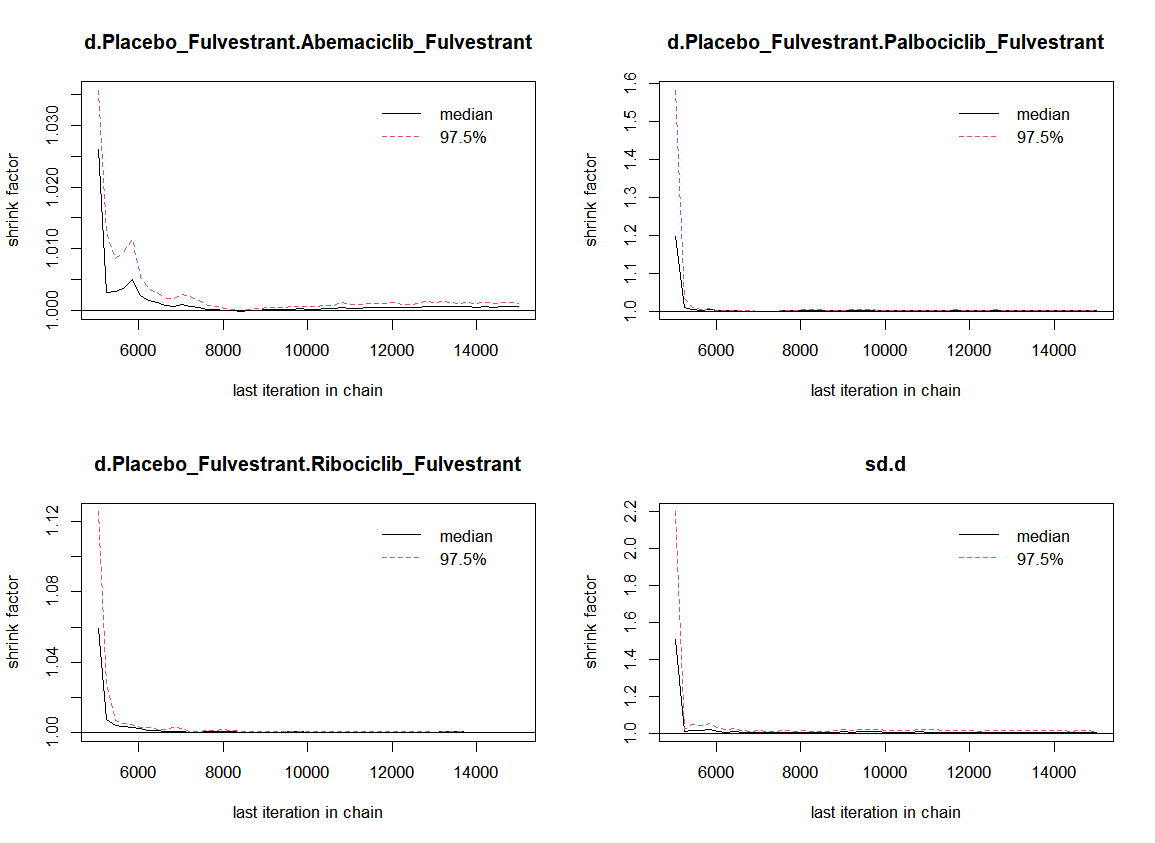
**

**Figure S5.6:** Convergence Diagnostic Plot of **AEs**

**
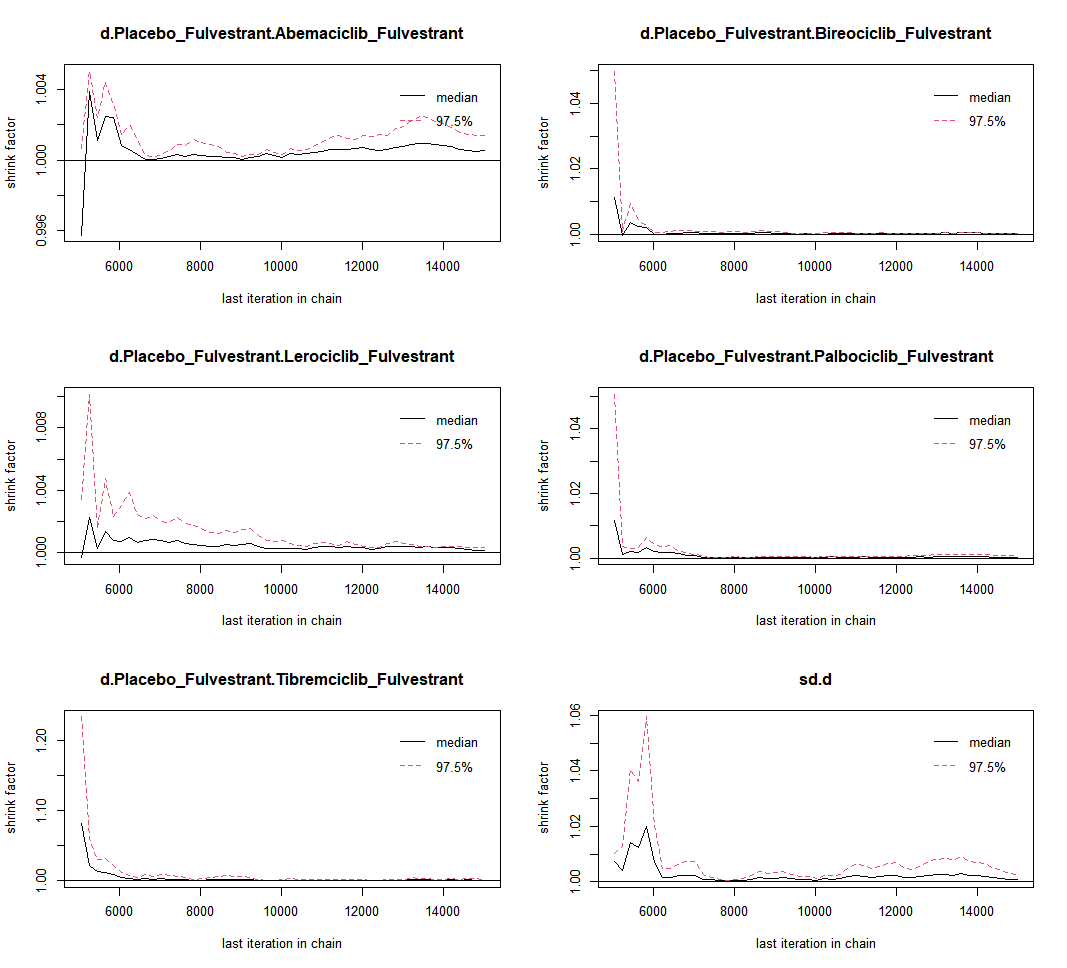
**

**Figure S5.7:** Convergence Diagnostic Plot of **Grade 3–4 AEs**

**
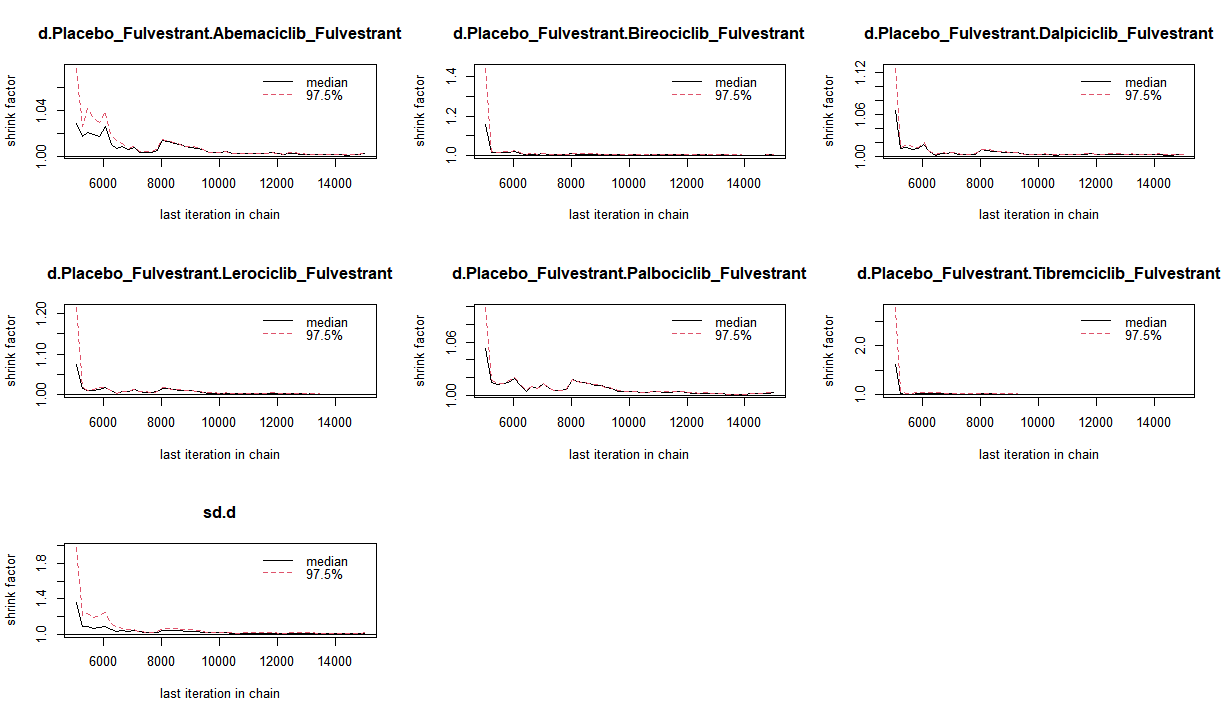
**

**Figure S5.8:** Convergence Diagnostic Plot of **SAEs**

**
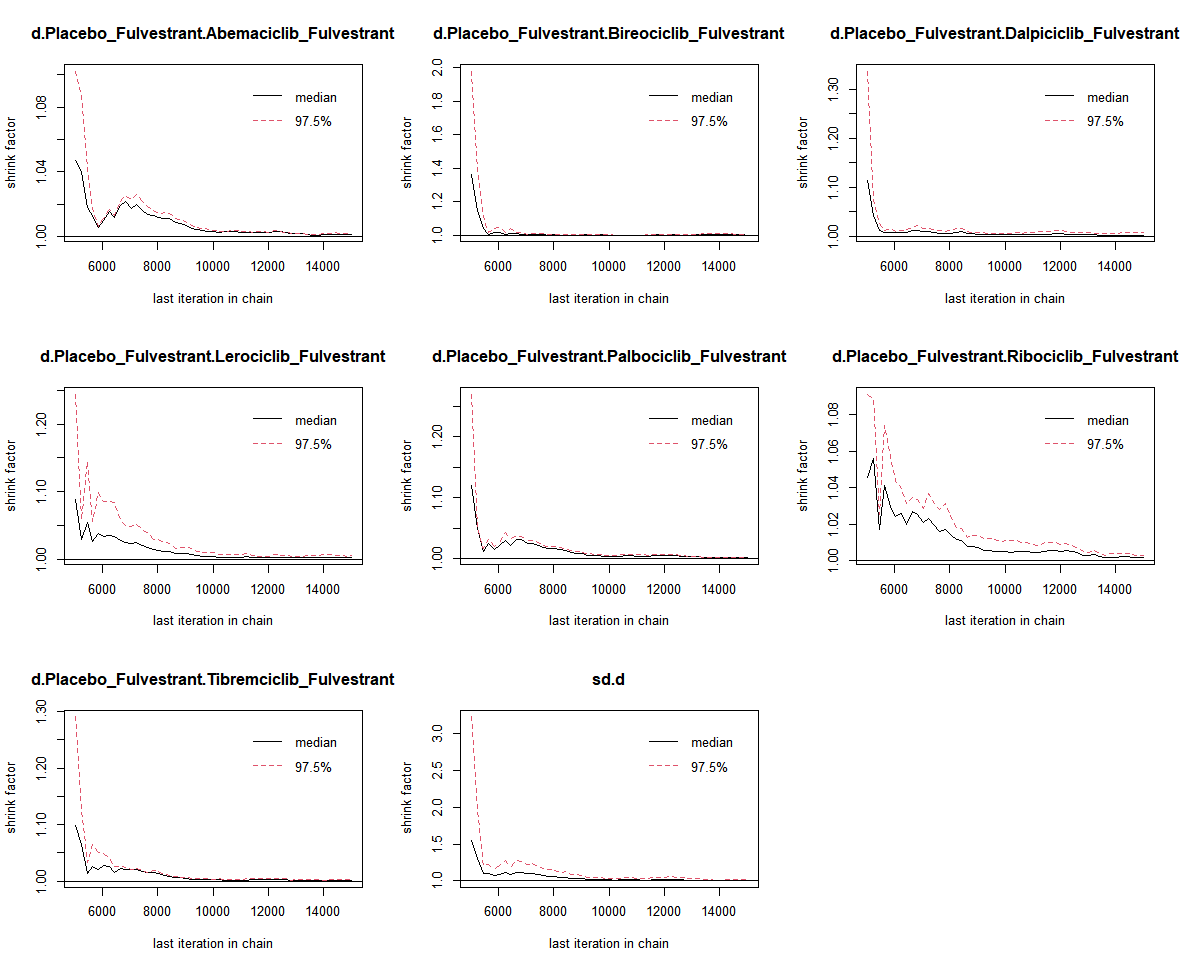
**

**Figure S5.9:** Convergence Diagnostic Plot of **Treatment discontinuation due to AEs**

**
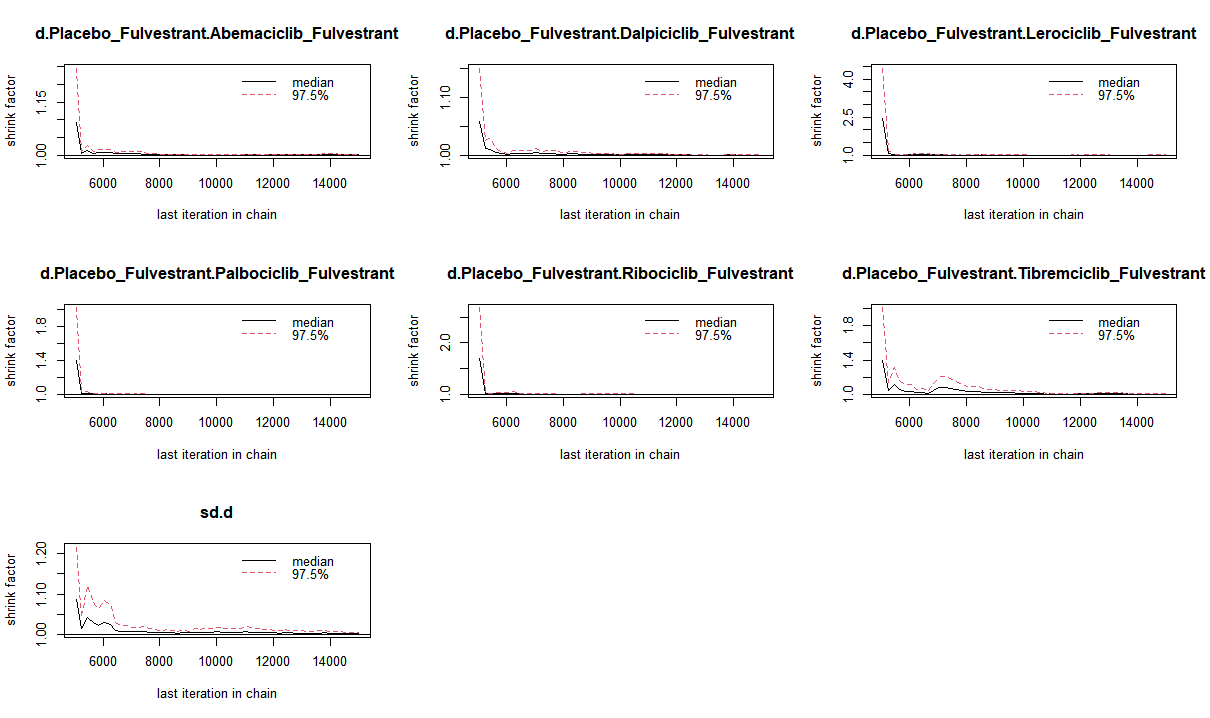
**

**Appendix 6: Network maps and forest plots of secondary outcomes**

**Figure S6.1**: Network map and forest plot of the effect on **AEs**

**
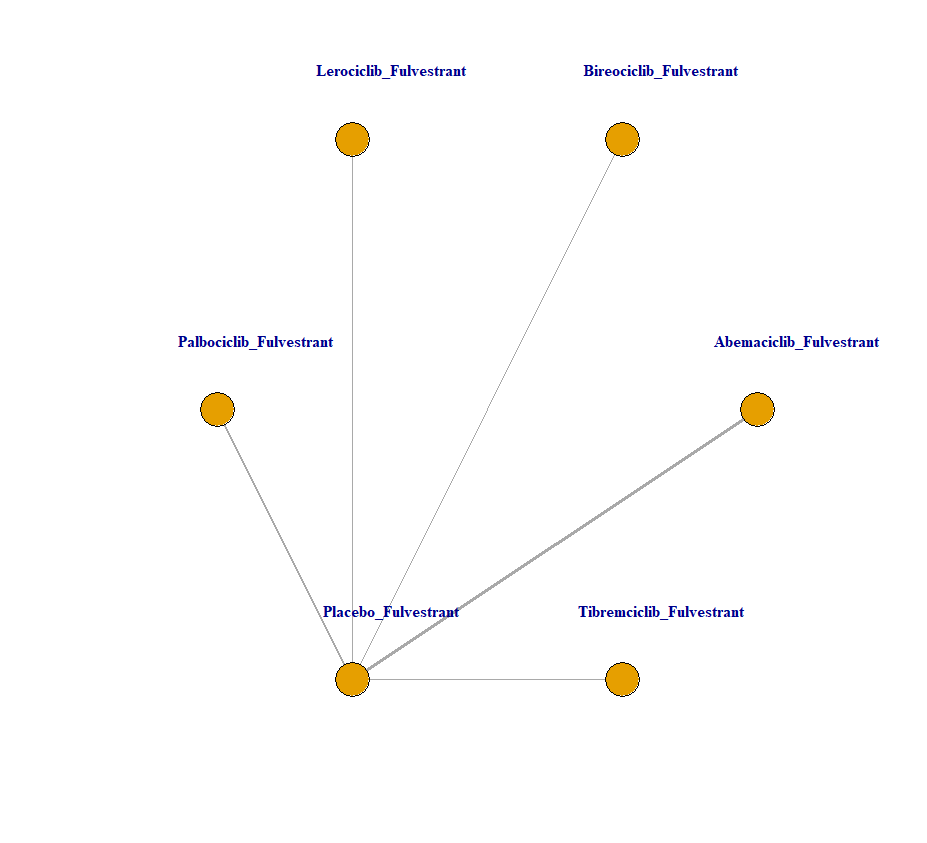
**

**Figure S6.2**: Network map and forest plot of the effect on **Grade 3–4 AEs**

**
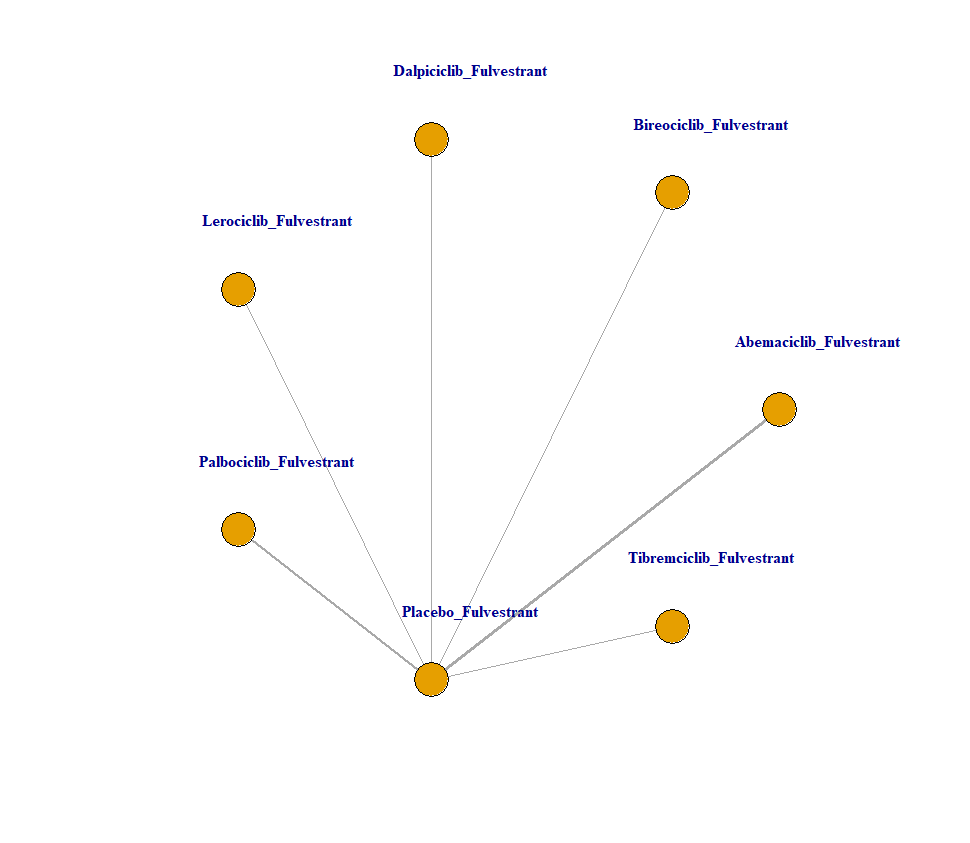
**

**Figure S6.3**: Network map and forest plot of the effect on **SAEs**

**
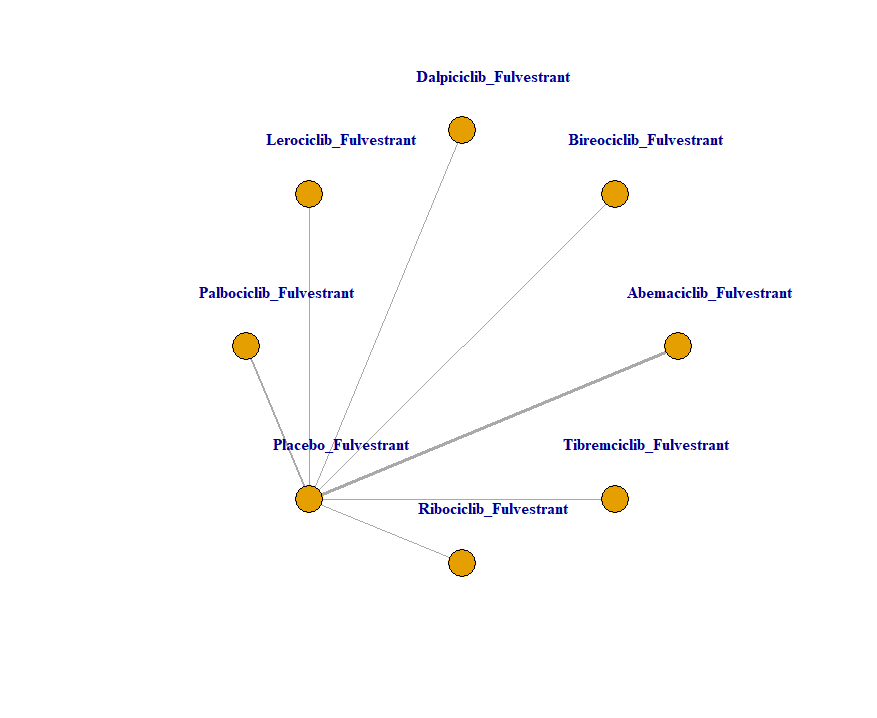
**

**Figure S6.4**: Network map and forest plot of the effect on **Treatment discontinuation due to AEs**

**
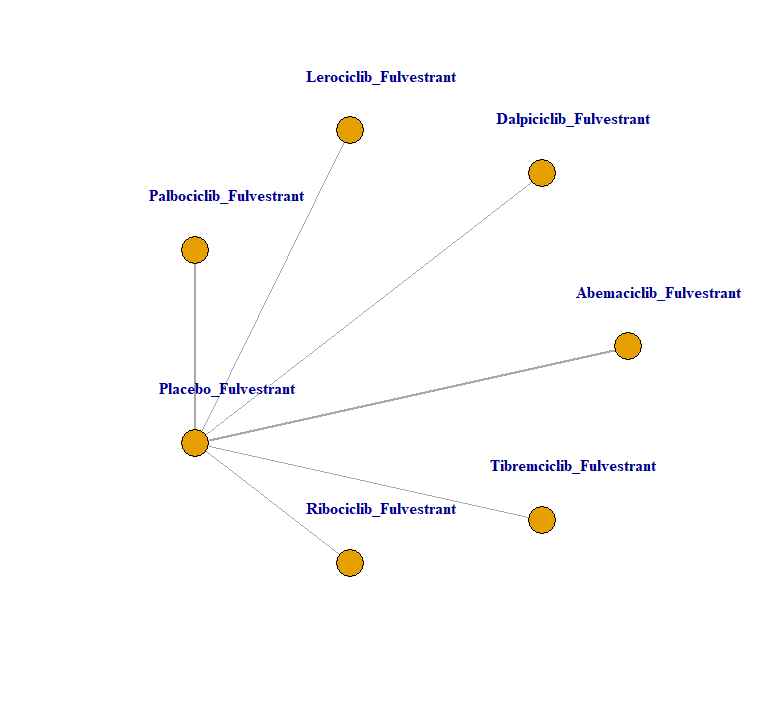
**

**Appendix 7: SUCRA and cumulative probability plots**

**Figure 7.1**: Cumulative ranking curve plots of CDK4/6 inhibitors for **PFS** in range network. Higher surface under the curve reflects lower probability of association with **PFS**.

**
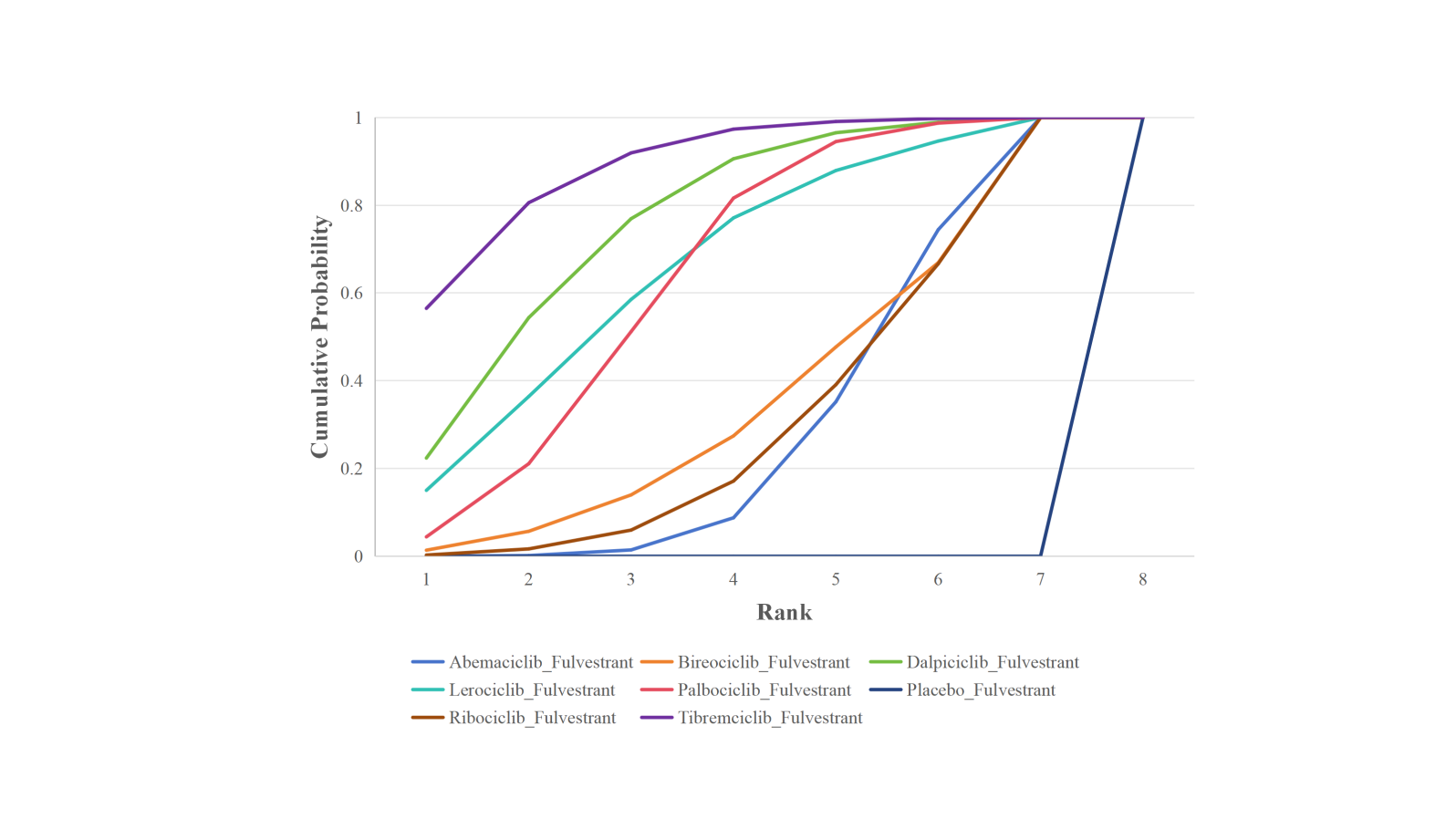
**

| **Intervention** | **SUCRA** |
| --- | --- |
| Tibremciclib_Fulvestrant | 89.26 |
| Dalpiciclib_Fulvestrant | 77.10 |
| Lerociclib_Fulvestrant | 67.26 |
| Palbociclib_Fulvestrant | 64.44 |
| Bireociclib_Fulvestrant | 37.50 |
| Ribociclib_Fulvestrant | 32.94 |
| Abemaciclib_Fulvestrant | 31.50 |
| Placebo_Fulvestrant | 0.01 |

**Figure 7.2**: Cumulative ranking curve plots of CDK4/6 inhibitors for **OS** in range network. Higher surface under the curve reflects lower probability of association with **OS** .


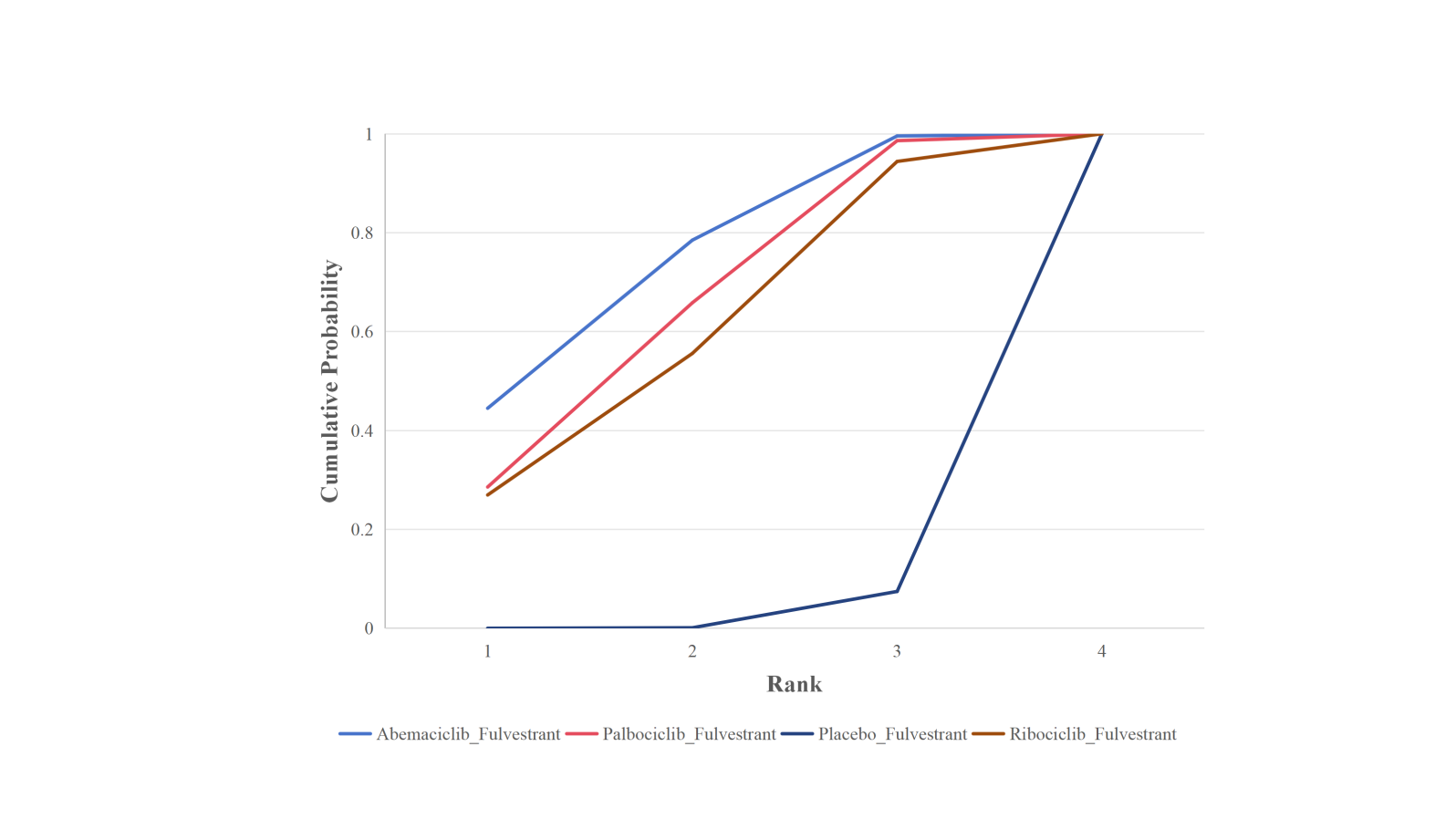


| **Intervention** | **SUCRA** |
| --- | --- |
| Abemaciclib_Fulvestrant | 74.24 |
| Palbociclib_Fulvestrant | 64.26 |
| Ribociclib_Fulvestrant | 58.87 |
| Placebo_Fulvestrant | 2.62 |

**Figure 7.3**: Cumulative ranking curve plots of CDK4/6 inhibitors for **ORR** in range network. Higher surface under the curve reflects lower probability of association with **ORR**.


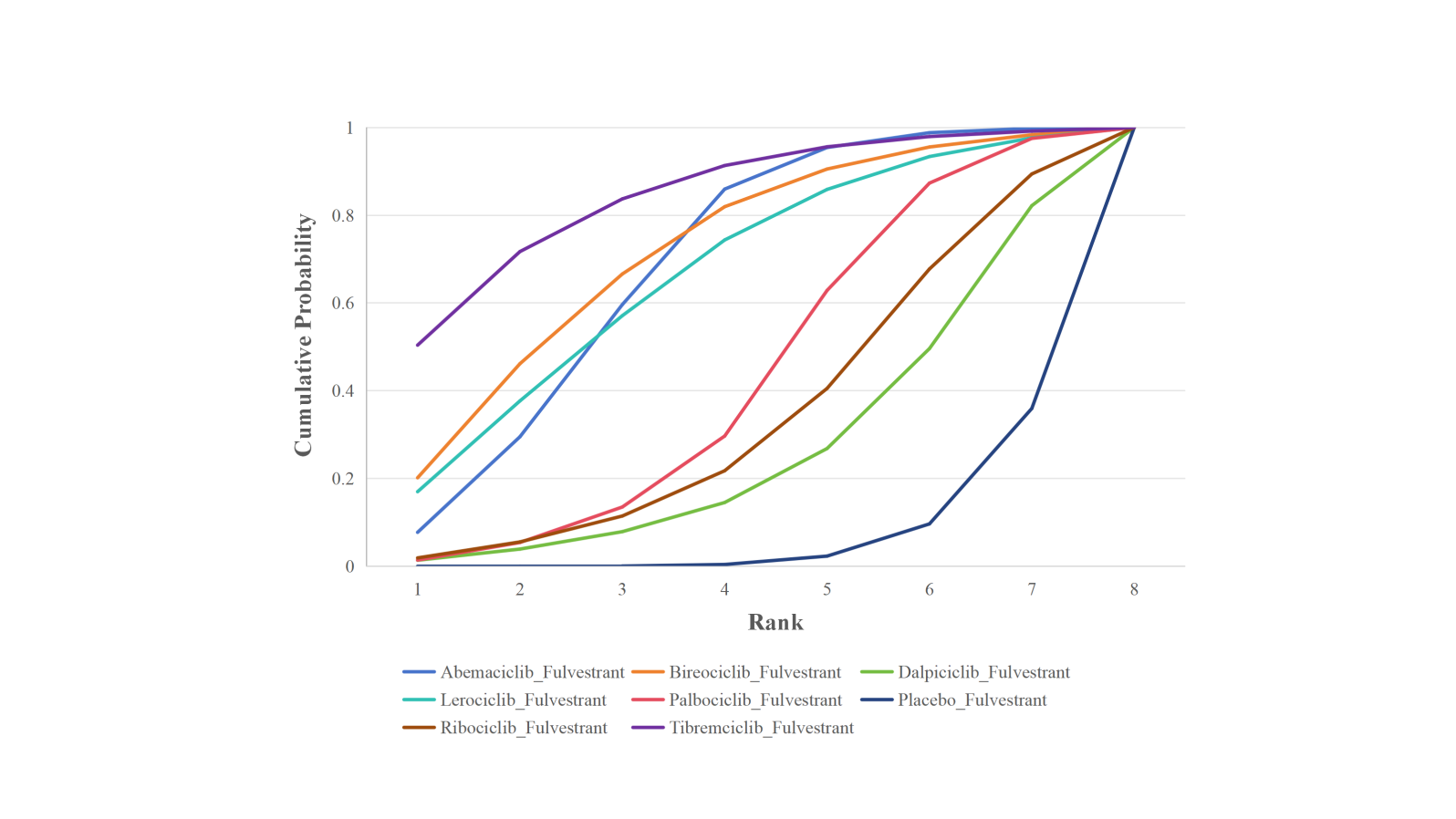


| **Intervention** | **SUCRA** |
| --- | --- |
| Tibremciclib_Fulvestrant | 84.27 |
| Bireociclib_Fulvestrant | 71.33 |
| Abemaciclib_Fulvestrant | 68.13 |
| Lerociclib_Fulvestrant | 66.15 |
| Palbociclib_Fulvestrant | 42.53 |
| Ribociclib_Fulvestrant | 34.06 |
| Dalpiciclib_Fulvestrant | 26.62 |
| Placebo_Fulvestrant | 6.92 |

**Figure 7.4**: Cumulative ranking curve plots of CDK4/6 inhibitors for **CBR** in range network. Higher surface under the curve reflects lower probability of association with **CBR**.


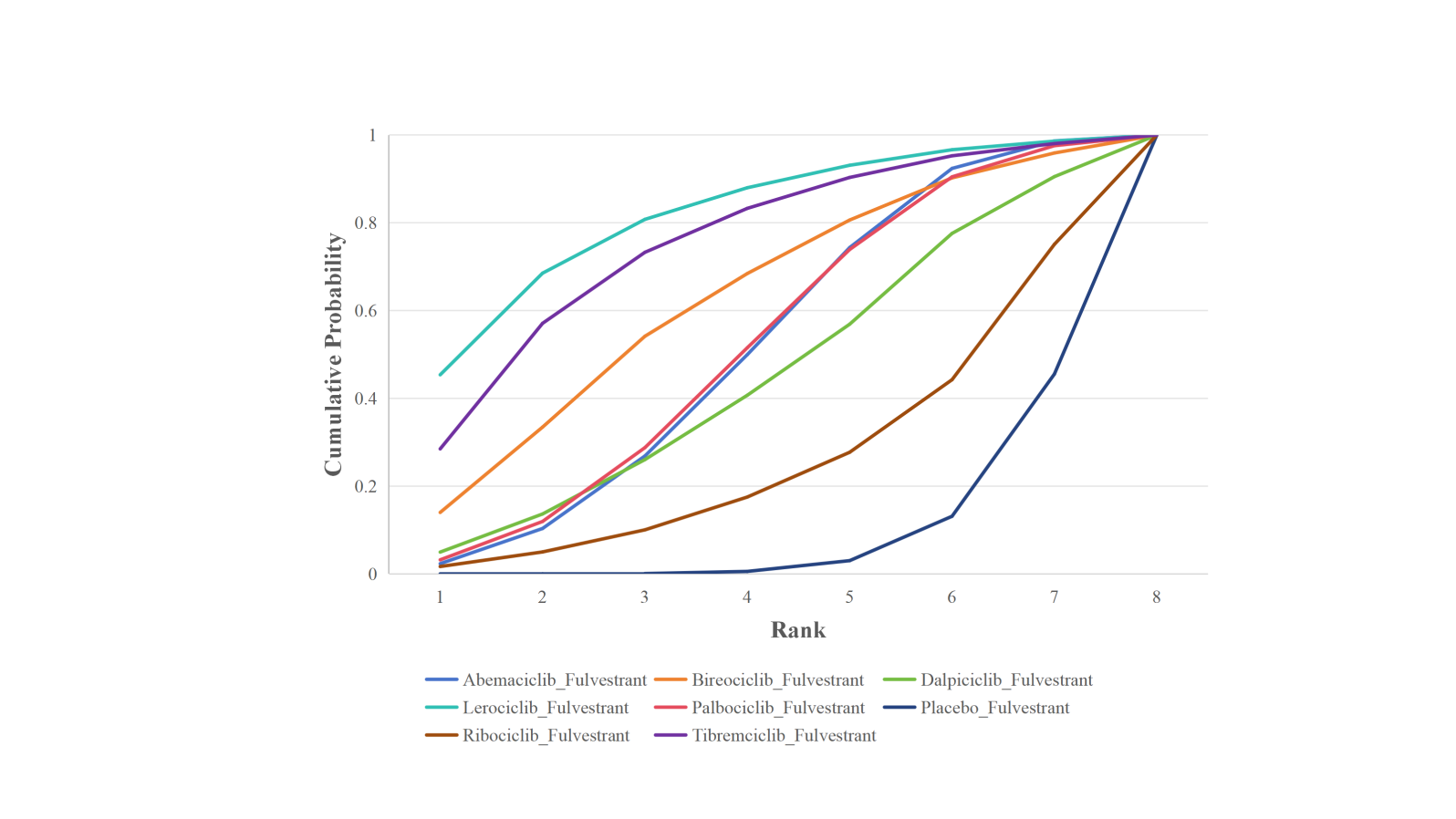


| **Intervention** | **SUCRA** |
| --- | --- |
| Lerociclib_Fulvestrant | 81.58 |
| Tibremciclib_Fulvestrant | 75.12 |
| Bireociclib_Fulvestrant | 62.40 |
| Palbociclib_Fulvestrant | 51.06 |
| Abemaciclib_Fulvestrant | 50.70 |
| Dalpiciclib_Fulvestrant | 44.34 |
| Ribociclib_Fulvestrant | 25.90 |
| Placebo_Fulvestrant | 8.90 |

**Figure 7.5**: Cumulative ranking curve plots of CDK4/6 inhibitors for **QoL** in range network. Higher surface under the curve reflects lower probability of association with **QoL**.


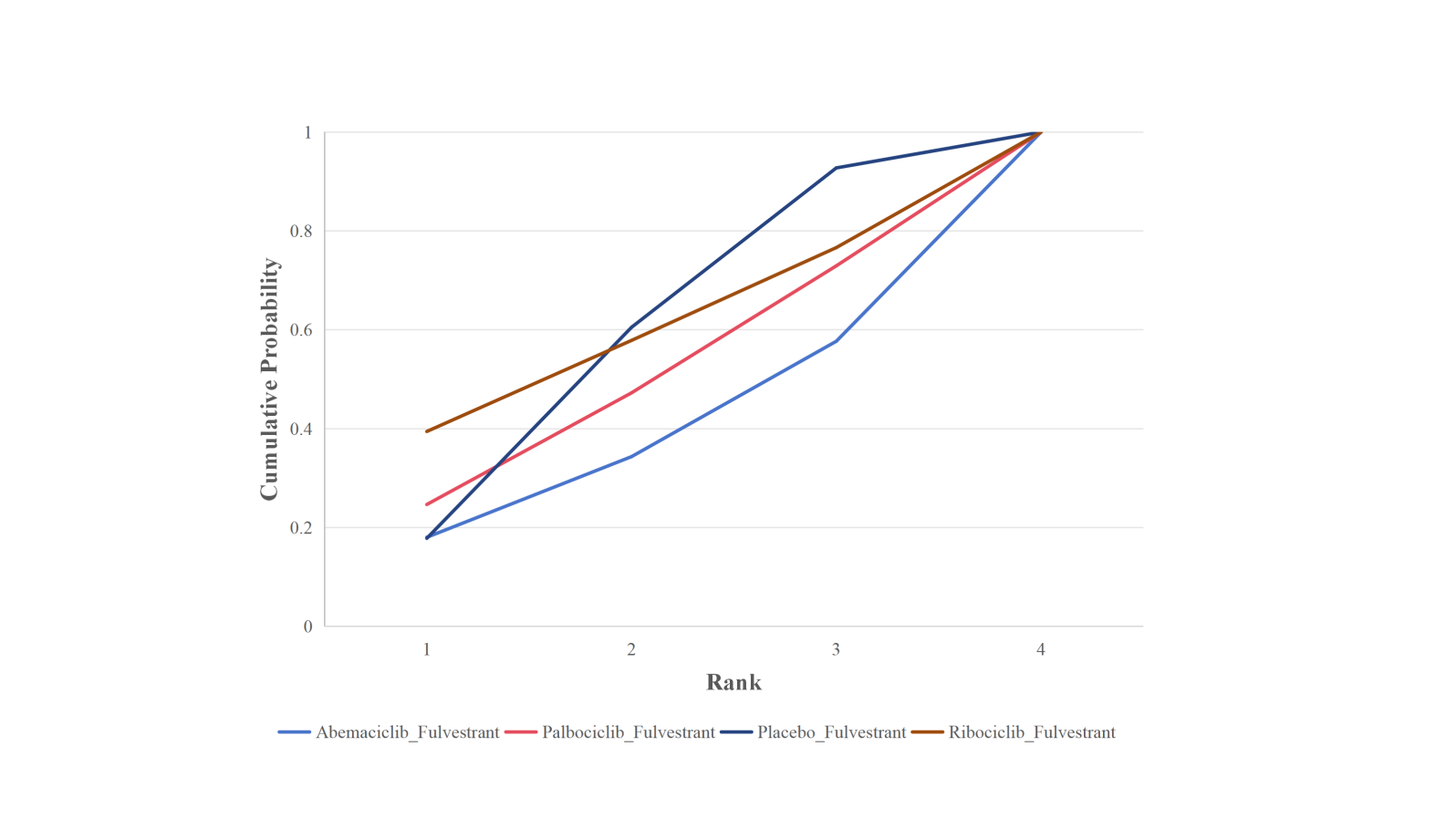


| **Intervention** | **SUCRA** |
| --- | --- |
| Ribociclib_Fulvestrant | 57.98 |
| Placebo_Fulvestrant | 57.03 |
| Palbociclib_Fulvestrant | 48.29 |
| Abemaciclib_Fulvestrant | 36.70 |

**Figure 7.6**: Cumulative ranking curve plots of CDK4/6 inhibitors for **AEs** in range network. Higher surface under the curve reflects lower probability of association with **AEs**.


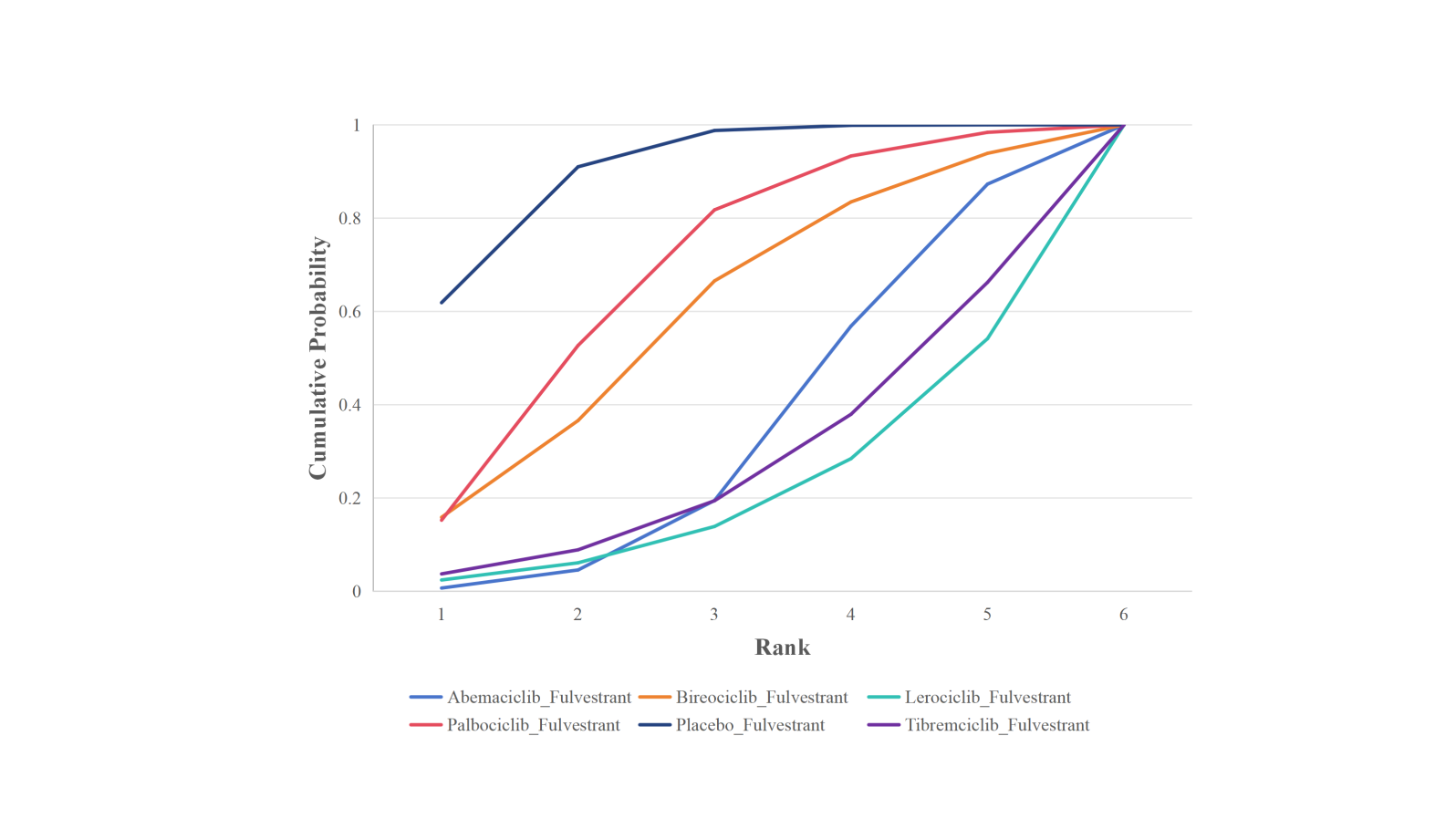


| **Intervention** | **SUCRA** |
| --- | --- |
| Placebo_Fulvestrant | 90.31 |
| Palbociclib_Fulvestrant | 68.29 |
| Bireociclib_Fulvestrant | 59.29 |
| Abemaciclib_Fulvestrant | 33.80 |
| Tibremciclib_Fulvestrant | 27.27 |
| Lerociclib_Fulvestrant | 21.03 |

**Figure 7.7**: Cumulative ranking curve plots of CDK4/6 inhibitors for **Grade 3–4 AEs** in range network. Higher surface under the curve reflects lower probability of association with **Grade 3–4 AEs**.


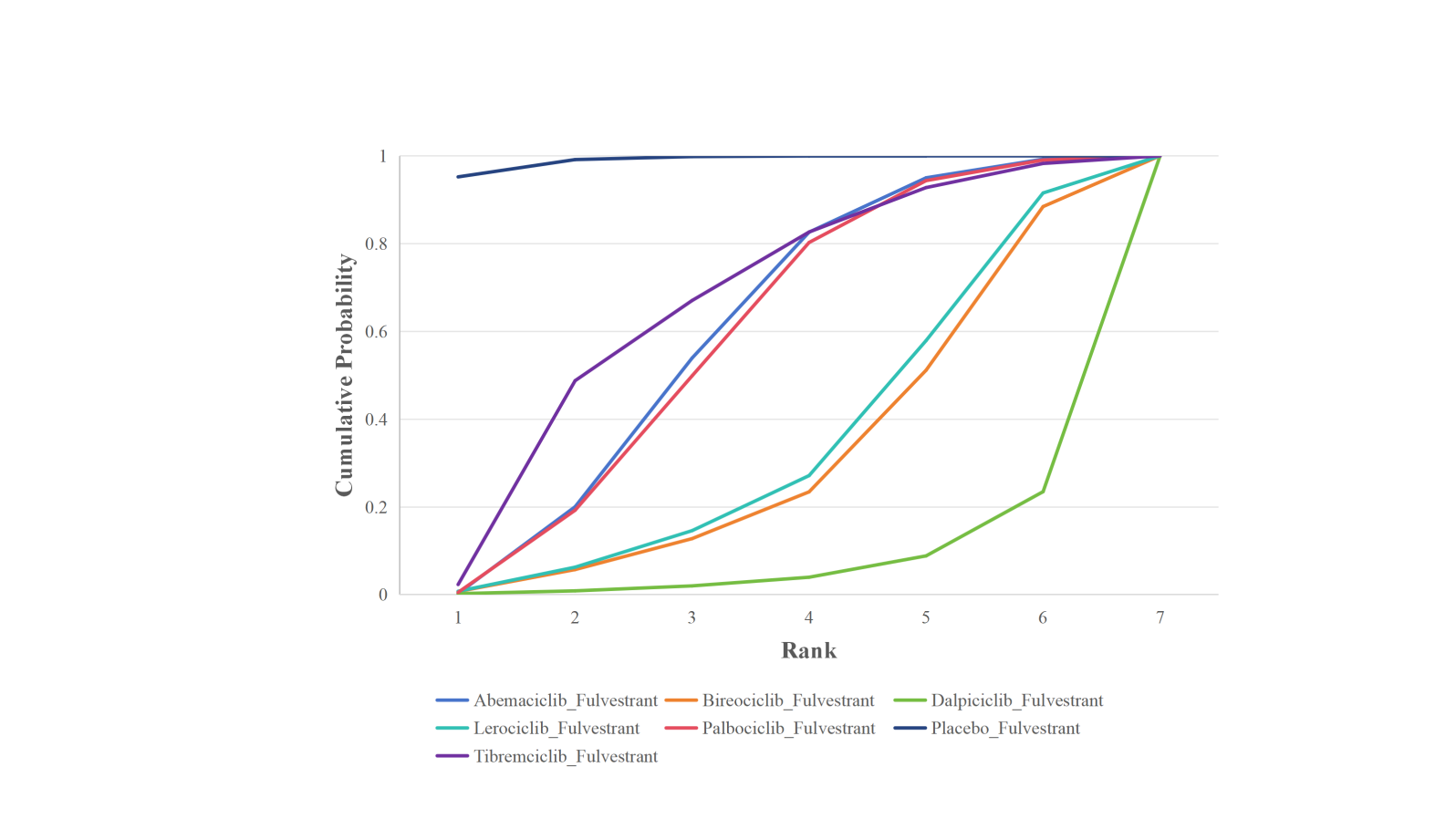


| **Intervention** | **SUCRA** |
| --- | --- |
| Placebo_Fulvestrant | 99.02 |
| Tibremciclib_Fulvestrant | 65.30 |
| Abemaciclib_Fulvestrant | 58.51 |
| Palbociclib_Fulvestrant | 57.21 |
| Lerociclib_Fulvestrant | 33.03 |
| Bireociclib_Fulvestrant | 30.37 |
| Dalpiciclib_Fulvestrant | 6.56 |

**Figure 7.8**: Cumulative ranking curve plots of CDK4/6 inhibitors for **SAEs** in range network. Higher surface under the curve reflects lower probability of association with **SAEs**.


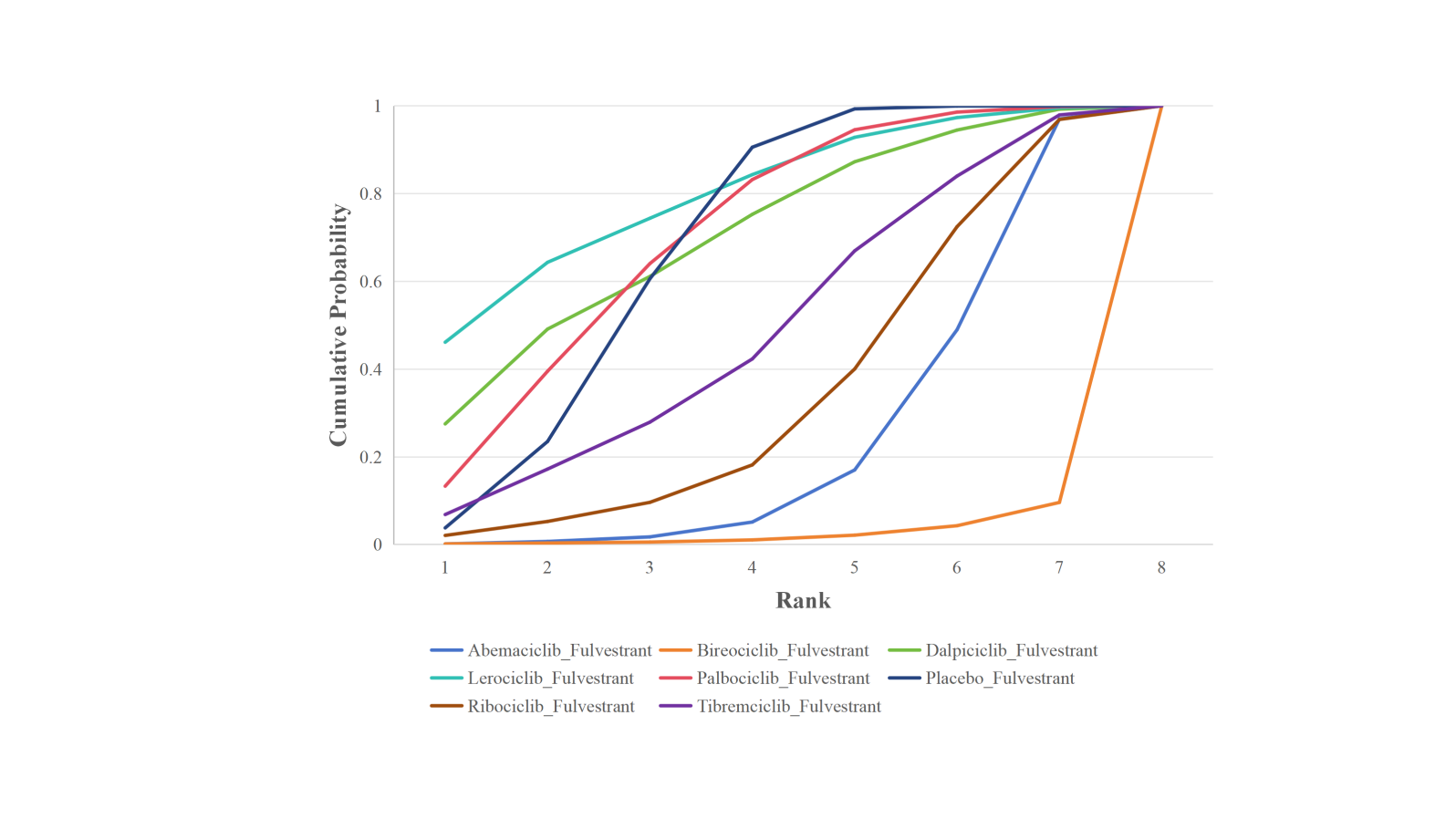


| **Intervention** | **SUCRA** |
| --- | --- |
| Lerociclib_Fulvestrant | 79.83 |
| Dalpiciclib_Fulvestrant | 70.56 |
| Palbociclib_Fulvestrant | 70.43 |
| Placebo_Fulvestrant | 68.24 |
| Tibremciclib_Fulvestrant | 49.02 |
| Ribociclib_Fulvestrant | 34.93 |
| Abemaciclib_Fulvestrant | 24.39 |
| Bireociclib_Fulvestrant | 2.60 |

**Figure 7.9**: Cumulative ranking curve plots of CDK4/6 inhibitors for **Treatment discontinuation** in range network. Higher surface under the curve reflects lower probability of association with **Treatment discontinuation**.


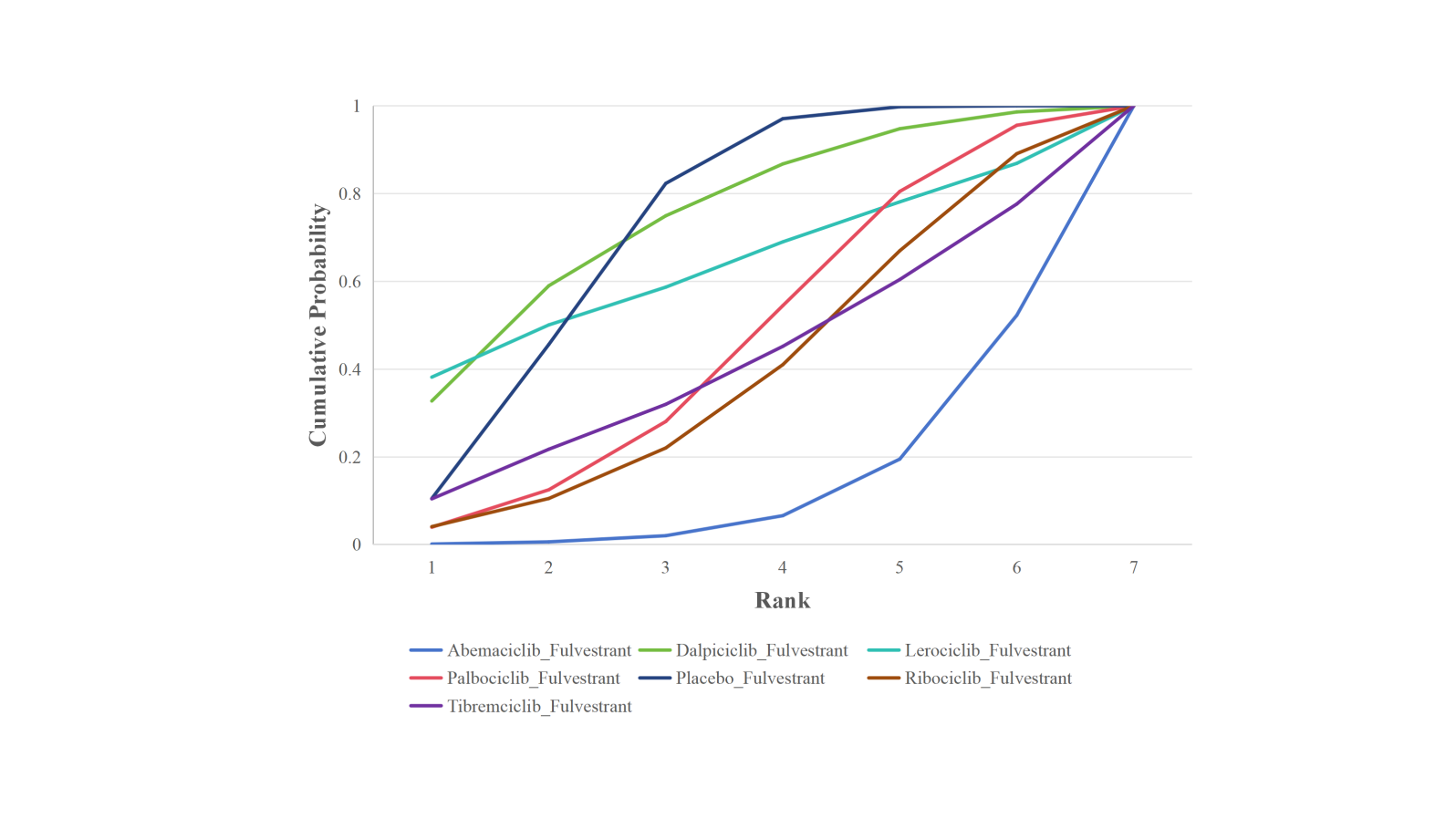


| **Intervention** | **SUCRA** |
| --- | --- |
| Dalpiciclib_Fulvestrant | 74.46 |
| Placebo_Fulvestrant | 72.54 |
| Lerociclib_Fulvestrant | 63.48 |
| Palbociclib_Fulvestrant | 45.83 |
| Tibremciclib_Fulvestrant | 41.22 |
| Ribociclib_Fulvestrant | 38.95 |
| Abemaciclib_Fulvestrant | 13.52 |

**Appendix 8: League table of Summary Estimates for CDK4/6 inhibitors on HR+/HER2– Advanced or Metastatic Breast Cancer Derived from Network Meta-analysis of 10 Trials**

**Table S8.1: PFS**

The columns represent the comparison of the row drug class to the column drug class. The rows represent the comparison of the row drug class to the column drug class. The effect estimates are expressed as HR and 95% CrI. For example, the HR in **PFS** for **ABE+FUL** compared to **BIR+FUL** is **1.02(0.7, 1.49)**. HR <1 favors the drug in the column, and HR >1 favors the drug in the row.

| **ABE+FUL** |  |  |  |  |  |  |  |
| --- | --- | --- | --- | --- | --- | --- | --- |
| 1.02 (0.7, 1.49) | **BIR+FUL** |  |  |  |  |  |  |
| 1.36 (0.97, 1.92) | 1.34 (0.83, 2.14) | **DAL+FUL** |  |  |  |  |  |
| 1.27 (0.85, 1.89) | 1.25 (0.75, 2.08) | 0.93 (0.57, 1.52) | **LERO+FUL** |  |  |  |  |
| 1.23 (0.95, 1.59) | 1.2 (0.8, 1.82) | 0.9 (0.62, 1.32) | 0.97 (0.63, 1.49) | **PAL+FUL** |  |  |  |
| 0.57 (0.5, 0.65) | 0.56 (0.39, 0.8) | 0.42 (0.31, 0.57) | 0.45 (0.31, 0.65) | 0.46 (0.37, 0.58) | **PLA+FUL** |  |  |
| 1 (0.75, 1.33) | 0.98 (0.64, 1.51) | 0.73 (0.49, 1.1) | 0.79 (0.5, 1.24) | 0.81 (0.58, 1.14) | 1.75 (1.36, 2.26) | **RIB+FUL** |  |
| 1.53 (1.07, 2.2) | 1.51 (0.93, 2.44) | 1.13 (0.71, 1.78) | 1.21 (0.73, 1.99) | 1.25 (0.84, 1.86) | 2.69 (1.92, 3.76) | 1.54 (1.01, 2.34) | **TIB+FUL** |

Note: PAL, palbociclib; RIB, ribociclib; ABE, abemaciclib; DAL, dalpiciclib; BIR, bireociclib; LERO, lerociclib; TIB, tibremciclib; PLA,placebo; FUL, fulvestrant.

**Table S8.2: OS**

The columns represent the comparison of the row drug class to the column drug class. The rows represent the comparison of the row drug class to the column drug class. The effect estimates are expressed as HR and 95% CrI. For example, the HR in **OS** for **ABE+FUL** compared to **PAL+FUL** is **0.96 (0.71, 1.3)**. HR <1 favors the drug in the column, and HR >1 favors the drug in the row.

| **ABE+FUL** |  |  |  |
| --- | --- | --- | --- |
| 0.96 (0.71, 1.3) | **PAL+FUL** |  |  |
| 0.76 (0.61, 0.94) | 0.79 (0.63, 0.98) | **PLA+FUL** |  |
| 0.94 (0.66, 1.34) | 0.98 (0.69, 1.39) | 1.25 (0.95, 1.64) | **RIB+FUL** |

Note: PAL, palbociclib; RIB, ribociclib; ABE, abemaciclib; PLA, placebo; FUL, fulvestrant.

**Table S8.3: ORR**

The columns represent the comparison of the row drug class to the column drug class. The rows represent the comparison of the row drug class to the column drug class. The effect estimates are expressed as RR and 95% CI. For example, the RR in **ORR** for **ABE+FUL** compared to **BIR+FUL** is **0.9 (0.25, 3.99)**. RR < 1 favors the drug in the row, and RR >1 favors the drug in the column.

| **ABE+FUL** |  |  |  |  |  |  |  |
| --- | --- | --- | --- | --- | --- | --- | --- |
| 0.9 (0.25, 3.99) | **BIR+FUL** |  |  |  |  |  |  |
| 1.94 (0.56, 8.72) | 2.17 (0.41, 11.55) | **DAL+FUL** |  |  |  |  |  |
| 0.99 (0.25, 4.4) | 1.09 (0.19, 5.77) | 0.5 (0.09, 2.66) | **LERO+FUL** |  |  |  |  |
| 1.47 (0.53, 4.78) | 1.63 (0.37, 6.68) | 0.75 (0.18, 3.01) | 1.5 (0.34, 6.48) | **PAL+FUL** |  |  |  |
| 2.69 (1.45, 6.08) | 2.99 (0.92, 9.79) | 1.37 (0.42, 4.37) | 2.75 (0.82, 9.6) | 1.82 (0.83, 4.33) | **PLA+FUL** |  |  |
| 1.71 (0.5, 7.51) | 1.9 (0.36, 10.14) | 0.87 (0.17, 4.4) | 1.75 (0.34, 9.55) | 1.16 (0.29, 4.94) | 0.64 (0.2, 2.06) | **RIB+FUL** |  |
| 0.67 (0.17, 2.96) | 0.74 (0.13, 4) | 0.34 (0.06, 1.84) | 0.67 (0.12, 3.79) | 0.45 (0.1, 2.02) | 0.25 (0.07, 0.82) | 0.39 (0.07, 2.06) | **TIB+FUL** |

Note: PAL, palbociclib; RIB, ribociclib; ABE, abemaciclib; DAL, dalpiciclib; BIR, bireociclib; LERO, lerociclib; TIB, tibremciclib; PLA,placebo; FUL, fulvestrant.

**Table S8.4: CBR**

The columns represent the comparison of the row drug class to the column drug class. The rows represent the comparison of the row drug class to the column drug class. The effect estimates are expressed as RR and 95% CI. For example, the RR in **CBR** for **ABE+FUL** compared to **BIR+FUL** is **0.89 (0.42, 1.96)**. RR < 1 favors the drug in the row, and RR >1 favors the drug in the column.

| **ABE+FUL** |  |  |  |  |  |  |  |
| --- | --- | --- | --- | --- | --- | --- | --- |
| 0.89 (0.42, 1.96) | **BIR+FUL** |  |  |  |  |  |  |
| 1.05 (0.51, 2.26) | 1.17 (0.47, 2.95) | **DAL+FUL** |  |  |  |  |  |
| 0.72 (0.34, 1.59) | 0.8 (0.31, 2.1) | 0.68 (0.27, 1.73) | **LERO+FUL** |  |  |  |  |
| 1 (0.56, 1.82) | 1.11 (0.49, 2.46) | 0.95 (0.43, 2.08) | 1.38 (0.61, 3.12) | **PAL+FUL** |  |  |  |
| 1.41 (0.98, 2.1) | 1.58 (0.81, 3.04) | 1.34 (0.7, 2.56) | 1.96 (0.99, 3.87) | 1.42 (0.9, 2.24) | **PLA+FUL** |  |  |
| 1.24 (0.6, 2.68) | 1.39 (0.56, 3.5) | 1.18 (0.48, 2.92) | 1.73 (0.69, 4.35) | 1.25 (0.58, 2.75) | 0.88 (0.47, 1.66) | **RIB+FUL** |  |
| 0.79 (0.38, 1.7) | 0.88 (0.35, 2.23) | 0.75 (0.3, 1.89) | 1.09 (0.43, 2.85) | 0.79 (0.36, 1.77) | 0.56 (0.29, 1.07) | 0.63 (0.26, 1.56) | **TIB+FUL** |

Note: PAL, palbociclib; RIB, ribociclib; ABE, abemaciclib; DAL, dalpiciclib; BIR, bireociclib; LERO, lerociclib; TIB, tibremciclib; PLA,placebo; FUL, fulvestrant.

**Table S8.5: QoL**

The columns represent the comparison of the row drug class to the column drug class. The rows represent the comparison of the row drug class to the column drug class. The effect estimates are expressed as MD and 95% CI. For example, the MD in **QoL** for **ABE+FUL** compared to **PAL+FUL** is -**0.97 (-11.27, 10.49)**. MD < 0 favors the drug in the row, and MD >0 favors the drug in the column.

| **ABE+FUL** |  |  |  |
| --- | --- | --- | --- |
| -0.97 (-11.27, 10.49) | **PAL+FUL** |  |  |
| -1.47 (-10.05, 7.14) | -0.55 (-7.77, 5.76) | **PLA+FUL** |  |
| -1.83 (-14.63, 10.56) | -0.96 (-12.98, 9.96) | -0.37 (-9.65, 8.74) | **RIB+FUL** |

Note: PAL, palbociclib; RIB, ribociclib; ABE, abemaciclib; PLA,placebo; FUL, fulvestrant.

**Table S8.6: AEs**

The columns represent the comparison of the row drug class to the column drug class. The rows represent the comparison of the row drug class to the column drug class. The effect estimates are expressed as RR and 95% CI. For example, the RR in **AEs** for **ABE+FUL** compared to **BIR+FUL** is **1.07 (0.83, 1.42)**. RR < 1 favors the drug in the column, and RR >1 favors the drug in the row.

| **ABE+FUL** |  |  |  |  |  |
| --- | --- | --- | --- | --- | --- |
| 1.07 (0.83, 1.42) | **BIR+FUL** |  |  |  |  |
| 0.95 (0.73, 1.25) | 0.88 (0.63, 1.22) | **LERO+FUL** |  |  |  |
| 1.1 (0.9, 1.37) | 1.02 (0.77, 1.35) | 1.16 (0.88, 1.54) | **PAL+FUL** |  |  |
| 1.16 (1.02, 1.34) | 1.08 (0.86, 1.35) | 1.22 (0.97, 1.55) | 1.05 (0.9, 1.24) | **PLA+FUL** |  |
| 0.97 (0.74, 1.28) | 0.9 (0.64, 1.25) | 1.02 (0.73, 1.43) | 0.88 (0.66, 1.17) | 0.84 (0.65, 1.06) | **TIB+FUL** |

Note: PAL, palbociclib; ABE, abemaciclib; BIR, bireociclib; LERO, lerociclib; TIB, tibremciclib; PLA,placebo; FUL, fulvestrant.

**Table S8.7: Grade 3-4 AEs**

The columns represent the comparison of the row drug class to the column drug class. The rows represent the comparison of the row drug class to the column drug class. The effect estimates are expressed as RR and 95% CI. For example, the RR in **AEs** for **ABE+FUL** compared to **BIR+FUL** is **0.68 (0.23, 2.06)**. RR < 1 favors the drug in the column, and RR >1 favors the drug in the row.

| **ABE+FUL** |  |  |  |  |  |  |
| --- | --- | --- | --- | --- | --- | --- |
| 0.68 (0.23, 2.06) | **BIR+FUL** |  |  |  |  |  |
| 0.4 (0.14, 1.2) | 0.59 (0.15, 2.21) | **DAL+FUL** |  |  |  |  |
| 0.71 (0.26, 2.12) | 1.04 (0.29, 4.1) | 1.78 (0.5, 6.61) | **LERO+FUL** |  |  |  |
| 0.98 (0.47, 2.32) | 1.45 (0.48, 4.58) | 2.48 (0.84, 7.54) | 1.39 (0.46, 4.14) | **PAL+FUL** |  |  |
| 2.72 (1.7, 4.8) | 3.99 (1.61, 10.76) | 6.82 (2.76, 17.45) | 3.82 (1.53, 9.64) | 2.76 (1.48, 5.02) | **PLA+FUL** |  |
| 1.13 (0.41, 3.34) | 1.65 (0.45, 6.32) | 2.8 (0.76, 10.27) | 1.58 (0.44, 5.69) | 1.14 (0.38, 3.4) | 0.41 (0.17, 1.01) | **TIB+FUL** |

Note: PAL, palbociclib; ABE, abemaciclib; DAL, dalpiciclib; BIR, bireociclib; LERO, lerociclib; TIB, tibremciclib; PLA,placebo; FUL, fulvestrant.

**Table S8.8: SAEs**

The columns represent the comparison of the row drug class to the column drug class. The rows represent the comparison of the row drug class to the column drug class. The effect estimates are expressed as RR and 95% CI. For example, the RR in **SAEs** for **ABE+FUL** compared to **BIR+FUL** is **0.25 (0.03, 1.42)**. RR < 1 favors the drug in the column, and RR >1 favors the drug in the row.

| **ABE+FUL** |  |  |  |  |  |  |  |
| --- | --- | --- | --- | --- | --- | --- | --- |
| 0.25 (0.03, 1.42) | **BIR+FUL** |  |  |  |  |  |  |
| 2.37 (0.57, 9.55) | 9.58 (1.19, 92.14) | **DAL+FUL** |  |  |  |  |  |
| 2.93 (0.73, 12.82) | 11.92 (1.48, 121.85) | 1.25 (0.21, 7.66) | **LERO+FUL** |  |  |  |  |
| 2.24 (0.81, 6.11) | 8.95 (1.55, 70.12) | 0.94 (0.22, 4.19) | 0.76 (0.16, 3.26) | **PAL+FUL** |  |  |  |
| 2.14 (1.14, 4.26) | 8.56 (1.76, 59.29) | 0.9 (0.26, 3.25) | 0.73 (0.19, 2.61) | 0.96 (0.46, 2.1) | **PLA+FUL** |  |  |
| 1.2 (0.35, 4.3) | 4.85 (0.71, 41.93) | 0.5 (0.1, 2.64) | 0.41 (0.08, 2.15) | 0.54 (0.15, 2.04) | 0.56 (0.19, 1.6) | **RIB+FUL** |  |
| 1.58 (0.4, 6.15) | 6.26 (0.84, 57.41) | 0.66 (0.12, 3.83) | 0.53 (0.09, 3.01) | 0.7 (0.17, 2.88) | 0.74 (0.22, 2.33) | 1.3 (0.26, 6.3) | **TIB+FUL** |

Note: PAL, palbociclib; RIB, ribociclib; ABE, abemaciclib; DAL, dalpiciclib; BIR, bireociclib; LERO, lerociclib; TIB, tibremciclib; PLA,placebo; FUL, fulvestrant.

**Table S8.9: Treatment discontinuation**

The columns represent the comparison of the row drug class to the column drug class. The rows represent the comparison of the row drug class to the column drug class. The effect estimates are expressed as RR and 95% CI. For example, the RR in **Treatment discontinuation** for **ABE+FUL** compared to **DAL+FUL** is **7.13(0.69, 89.37)**. RR < 1 favors the drug in the column, and RR >1 favors the drug in the row.

| **ABE+FUL** |  |  |  |  |  |  |
| --- | --- | --- | --- | --- | --- | --- |
| 7.13 (0.69, 89.37) | **DAL+FUL** |  |  |  |  |  |
| 6.41 (0.1, 379.83) | 0.86 (0.01, 63.97) | **LERO+FUL** |  |  |  |  |
| 3.04 (0.41, 24.5) | 0.42 (0.04, 4.74) | 0.5 (0.01, 29.71) | **PAL+FUL** |  |  |  |
| 5.92 (1.57, 27.84) | 0.82 (0.12, 5.79) | 0.96 (0.02, 46.42) | 1.95 (0.47, 8.58) | **PLA+FUL** |  |  |
| 2.43 (0.23, 29.25) | 0.33 (0.02, 5.27) | 0.38 (0.01, 29.97) | 0.79 (0.07, 8.83) | 0.41 (0.06, 2.7) | **RIB+FUL** |  |
| 2.46 (0.07, 47.9) | 0.34 (0.01, 8.7) | 0.36 (0, 40.3) | 0.8 (0.02, 15.5) | 0.42 (0.01, 5.21) | 1 (0.02, 24.89) | **TIB+FUL** |

Note: PAL, palbociclib; RIB, ribociclib; ABE, abemaciclib; DAL, dalpiciclib; LERO, lerociclib; TIB, tibremciclib; PLA,placebo; FUL, fulvestrant.
